# A systems pharmacology model for inflammatory bowel disease

**DOI:** 10.1371/journal.pone.0192949

**Published:** 2018-03-07

**Authors:** Violeta Balbas-Martinez, Leire Ruiz-Cerdá, Itziar Irurzun-Arana, Ignacio González-García, An Vermeulen, José David Gómez-Mantilla, Iñaki F. Trocóniz

**Affiliations:** 1 Pharmacometrics & Systems Pharmacology, Department of Pharmacy and Pharmaceutical Technology, School of Pharmacy and Nutrition, University of Navarra, Pamplona, Spain; 2 IdiSNA, Navarra Institute for Health Research, Pamplona, Spain; 3 Janssen Research and Development, a division of Janssen Pharmaceutical NV, Beerse, Belgium; 4 Laboratory of Medical Biochemistry and Clinical Analysis, Faculty of Pharmaceutical Sciences, Ghent, Belgium; National Cancer Institute, UNITED STATES

## Abstract

**Motivation:**

The literature on complex diseases is abundant but not always quantitative. This is particularly so for Inflammatory Bowel Disease (IBD), where many molecular pathways are qualitatively well described but this information cannot be used in traditional quantitative mathematical models employed in drug development. We propose the elaboration and validation of a logic network for IBD able to capture the information available in the literature that will facilitate the identification/validation of therapeutic targets.

**Results:**

In this article, we propose a logic model for Inflammatory Bowel Disease (IBD) which consists of 43 nodes and 298 qualitative interactions. The model presented is able to describe the pathogenic mechanisms of the disorder and qualitatively describes the characteristic chronic inflammation. A perturbation analysis performed on the IBD network indicates that the model is robust. Also, as described in clinical trials, a simulation of anti-TNFα, anti-IL2 and Granulocyte and Monocyte Apheresis showed a decrease in the Metalloproteinases node (MMPs), which means a decrease in tissue damage. In contrast, as clinical trials have demonstrated, a simulation of anti-IL17 and anti-IFNγ or IL10 overexpression therapy did not show any major change in MMPs expression, as corresponds to a failed therapy. The model proved to be a promising *in silico* tool for the evaluation of potential therapeutic targets, the identification of new IBD biomarkers, the integration of IBD polymorphisms to anticipate responders and non-responders and can be reduced and transformed in quantitative model/s.

## Introduction

Inflammatory bowel disease (IBD) is a complex gastrointestinal tract disorder characterized by a functional impairment of the gut wall affecting patients´ quality of life [1,2]. IBD includes ulcerative colitis (UC) and Crohn's disease (CD). The natural course of IBD is highly variable [3–6] and its etiology is still unknown. The incidence of IBD has dramatically increased worldwide over the past 50 years [7], reaching levels of 24.3 per 100,000 person-years in UC and 20.2 per 100,000 person-years in CD in the developed countries [8].

There is current evidence that Interleukin 6 (IL6), Tumour necrosis factor-alpha (TNFα), Interferon Gamma (IFNƔ), Interleukin 1 beta (IL1ß), Interleukin 22 (IL22), Interleukin 17 (IL17) and Natural Killer cells (NK), among other signalling pathways, play relevant roles in the pathogenesis of IBD, which is a reflection of the complexity of that physiological system [9–12]. That complexity indicates that a universal treatment for IBD may not be feasible for the vast majority of patients [13,14]. In fact, current biological approved treatments are only palliative with a high percentage of non-responders. For example, around 50% of IBD patients treated with the current standard of care, Infliximab (an anti-TNFα) or Vedolizumab (an anti-α4β7 integrin) do not respond satisfactorily to therapy [15,16]. One characteristic of the current IBD biological treatments is that approved therapies target just one signalling pathway, which might explain the high rate of non-responders and the long-term inefficiency of most treatments [15,17]. In addition, there is evidence to suggest that optimal treatment for IBD should involve a combination of different drugs [18,19]. Therefore, there is a need, especially for complex alterations such as immune-mediated diseases, to change the paradigm of drug development, considering the main aspects (targets, cross-talking between pathways, therapy combination) from an integrative and computational perspective.

Given the aforementioned biological complexity of immune-mediated diseases and the fact that current longitudinal data associated with the most relevant elements of the system are scarce, a full parameterization of IBD related systems based on a differential equation model does not yet seem feasible. However, some attempts have been made to describe quantitatively the IBD systems. For example, Wendelsdorf et al., [20] built a quantitative model based on ordinary differential equations. However, some key disease elements, such as cytokines and T cells, were incorporated non-specifically (i.e., all types of cytokine were grouped under the generic element active cytokines) in the model structure, limiting its use to explore potential therapeutic targets. More recently, Dwivendi et al., [21], based on the results of a clinical trial with the anti–IL6R antibody, Tocilizumab, have developed a multiscale systems model in Crohn’s disease, limited to the IL6–mediated immune regulation pathway.

Network analysis represents a promising alternative in such data limited circumstances [22–24]. As many molecular pathways in IBD are qualitatively well described, interaction networks may be a suitable approach for characterizing IBD. These networks are simplified representations of biological systems in which the components of the system such as genes, proteins or cells are represented by nodes and the interactions between them by edges [25]. Boolean network models, originally introduced by Kauffman [26,27], represent the simplest discrete dynamic models. These models only assume two discrete states for the nodes of a network, ON or OFF, corresponding to the logic values 1 (active) or 0 (not active, but not necessarily absent) [28]. A well-designed logic model could generate predictive outcomes given a set of initial conditions. Qualitative, logical frameworks have emerged as relevant approaches with different applications, as demonstrated by a growing number of published models [29]. Complementing these applications, several groups have provided various methods and tools to support the definition and analysis of logical models, as it can be seen by the recent achievements of the Consortium for Logical Models and Tools (CoLoMoTo) in logical modelling [30]. There are already several tools for Boolean modeling of regulatory networks in which it is possible to define direct activation-inhibition relationships between the components of the network, such as BoolNet R [31] or GINsim [32]. More recently, the R package SPIDDOR (Systems Pharmacology for effIcient Drug Development On R) among others, has implemented new types of regulatory interactions and perturbations within the system, such as positive and negative modulators and the polymorphism-like alterations, which lead to richer dynamics between the nodes [28].

In the specific case of IBD, there have been initial attempts to develop network models. The multi-state modeling tool published by Mei et al., [33,34] can be considered a proof of concept in the application of these types of networks in mucosal immune responses. However, the number of elements that this model considers and integrates is limited for IBD characterization, since only six different cytokine types are included in the inter-cellular scale.

The objective of the current manuscript is to present a Boolean based network model incorporating the main cellular and protein components known to play a key role in IBD development and progression. The model has been built on well-established experimental knowledge, mostly of human origin, and only including animal data when no other source of information was available. Our aim has been to build a model structure facilitating key aspects in the treatment of immune mediated disease, such as the selection of the most promising combination therapies and the study of the impact of polymorphisms on pathway regulation, thus allowing patient stratification and personalized medicine.

This study provides the scientific community with a (i) computational IBD model implemented in SPIDDOR R package [28], which allows translation of Boolean models (excluding models enclosing temporal operators) to a standard Markup language in Systems Biology for qualitative models (SBML qual [35]) which promotes model interoperability, and (ii) a repository with the main and updated information known of the immune system and IBD, which shows model transparency and allows model reusability. The proposed IBD model can be easily expanded in size and complexity to incorporate new knowledge, or other type of information such as proteomic data. The model presented hereafter is general enough to serve as a skeleton for other relevant immune diseases such as Rheumatoid Arthritis, Psoriasis or Multiple Sclerosis.

The manuscript is organized as follows: In the next section, Results regarding the structure of the model can be graphically visualized, and the ability of the model to recreate certain alterations that have been reported in IBD is demonstrated, as well as the model’s capability to reproduce the results from recent clinical trials performed in IBD patients from a high-level perspective. Applications of the model, including its advantages and limitations are then discussed together with ideas for future research. Finally, the Methods section provides a detailed technical description (with the aid of supplementary material) of the network and a description of how simulations, collection, and representation of results have been performed.

## Results

### Graphical representation, repository, and Boolean functions

**Fig 1:**
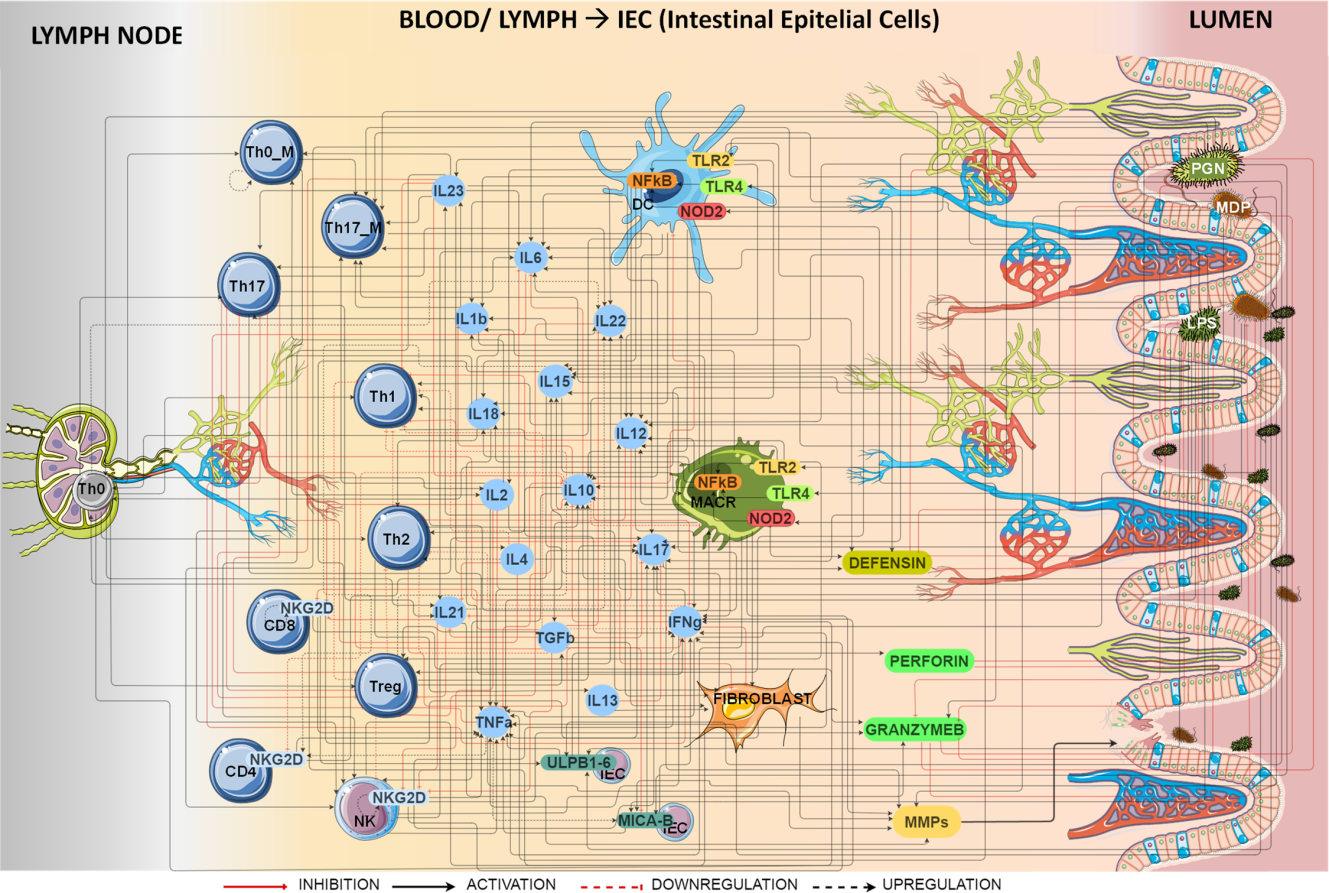
Graphical representation of IBD model. Nodes represent cells, proteins, bacterial antigens, receptors or ligands. Bacterial antigens trigger the IBD immune response through activation of different pattern recognition receptors (TLR2, TLR4 and NOD2) starting the innate and adaptive immune response. Reprinted from [36] under a CC BY license, with permission from the organizers of the 2016 International Conference on Systems Biology, original copyright 2016.

The graphical representation of the IBD network is shown in Fig 1. It consists of 43 nodes and 298 qualitative interactions located in three different physiological areas corresponding to (i) the lymph node, (ii) the blood and lymph circulatory system that irrigates the intestinal epithelial cells and (iii) the gut lumen.

Definition of all nodes and the full documented regulatory interactions conforming the model structure can be found in supporting information S1 Table and S2 Table, respectively. The S2 Table is fundamental to understand the rationale for the selection and implementation of the Boolean functions (BF). It was organized to provide a comprehensive summary of the 301 manuscripts (published over the last three decades) used to build the model, highlighting for example whether (i) a specific pathway was reported to be altered in IBD, or (ii) information was supported by human (more than the 80% of the network structure) or animal data.

The Boolean operators used to define the network model of IBD were: the NOT operator which is noted as “!”, the AND operator which is noted as “&” and the OR operator which is noted as “|”. Recent and innovative modulators and threshold operators previously described by Irurzun-Arana et al., 2017 [28] were also part of the arsenal of Boolean elements used in the model proposed (see S1 File for a detailed description of those additional Boolean elements).

Regarding the input selection, as it is assumed that IBD is caused by intestinal dysbiosis, an environment of different bacteria was recreated selecting three different antigens which are components of most Bacterial Gram positive and Gram negative. Therefore, during the development of the proposed model the following assumptions were made: First, there is a chronic exposure to bacterial antigens: Peptidoglycan (PGN), Lipopolysaccharide (LPS) and Muramyl dipeptide (MDP). PGN is a component of the cell wall of all bacteria, but in particular of gram-positive bacteria, LPS is a component of the outer membrane of Gram-negative bacteria [37], and MDP is a constituent of both Gram-positive and Gram-negative bacteria [38]. All three elicit strong immune responses and seem to play a critical role in the development and pathophysiology of IBD, as it has been hypothesized that the onset or relapse of IBD is triggered by an imbalance in self-microbiota composition than cannot be controlled by immune system [39]. Table 1 lists the initial conditions expressed by the corresponding BF, and shows that the nodes representing antigens are chronically expressed unless the natural antimicrobial peptides perforin (PERFOR), granzyme B (GRANZB) or defensins (DEF) become active.

**Table 1:** Boolean functions (BF) of the IBD model to simulate the initial conditions.

INITIAL CONDITIONS: CHRONIC EXPOSURE
$PGN=!\;({⋂}_{i=1}^{AG_{-}elim=6}{PERFOR}^{t-i}\;|{⋂}_{i=1}^{AG_{-}elim=6}{GRANZB}^{t-i}\;|\;{⋂}_{i=1}^{AG_{-}elim=6}{DEF}^{t-i})$
$MDP=!\;({⋂}_{i=1}^{AG_{-}elim=6}{PERFOR}^{t-i}\;|{⋂}_{i=1}^{AG_{-}elim=6}{GRANZB}^{t-i}\;|\;{⋂}_{i=1}^{AG_{-}elim=6}{DEF}^{t-i})$
$LPS=!\;({⋂}_{i=1}^{AG_{-}elim=6}{PERFOR}^{t-i}\;|{⋂}_{i=1}^{AG_{-}elim=6}{GRANZB}^{t-i}\;|\;{⋂}_{i=1}^{AG_{-}elim=6}{DEF}^{t-i})$

Second, there is an impairment in antigen elimination in IBD patients [1,40,41], simulated with the threshold operator Ag_elim = 6. The threshold operator means that PERFOR, GRANZB, or DEF inhibit antigen activation when any of these three nodes have been activated for at least 6 consecutive iterations (see Table 1).

Third, the final readout of the network model is the average expression of the output node, Metalloproteinases (MMPs). There is solid evidence that this group of proteins is directly associated with intestinal fibrosis and tissue damage in IBD [42–46] supporting their use as a relevant biomarker in clinical practice as proposed by O'Sullivan et al. [47]. As it can be seen in Table 2, the nodes that directly activate MMPs are the nodes that have relevant roles in the pathogenesis of IBD [9–12,42–44,46,48].

**Table 2:** Boolean functions (BF) of the IBD model for the internal and the output nodes.

**INTERNAL NODES**
*TLR*2 = *PGN*
*TLR*4 = *LPS*
*NOD*2 = *MDP*
*NFkB* = *TLR*2 | *NOD*2 | *TLR*4
*IL*6 = (***MACR*** & ***PGN***) | (*DC* & (*LPS* | *PGN*)) | (*Th*17 & *IL*23) | (*NFkB* &! (*IL*4 | *IL*10))
TNFa=((NFkB&LPS)|(MACR&(IL2|(IFNg&LPS)|PGN))|(NK&(MDP|PGN|LPS)&((IL2| IL12)&(IL2|IL15))|(FIBROBLAST&IFNg)|((CD4_NKG2D|CD8_NKG2D|NK_NKG2D)&(IEC_MICA_B| $IEC\_ULPB1\_6)))\&!\;({⋂}_{i=1}^{downreg_{cyt}=4}{IL10}^{t-i}\&\left({⋂}_{i=1}^{downreg_{cyt}=4}{TLR2}^{t-i}|{⋂}_{i=1}^{downreg_{cyt}=4}{TLR4}^{t-i}\right)\&TNFa)$
*TGFb* = (*Treg* | *MACR*)
$Th0={⋂}_{i=1}^{THR\_Th0=3}{LPS}^{t-i}|{⋂}_{i=1}^{THR\_Th0=3}{MDP}^{t-i}|{⋂}_{i=1}^{THR\_Th0=3}{PGN}^{t-i}$
*Th*0_*M* = (*Th*0 & (*IL*23 | *IL*12)) | *Th*0_*M*
*IL*18 = ((*MACR* | *DC*) & *LPS*) & *NFkB*
$IL1b=((MACR\;|\;DC)\&LPS\&NFkB)\&!\;(IL1b\&{⋃}_{i=1}^{downreg\_cyt=4}{IL10}^{t-i})$
IFNg=((NK&(PGN|LPS|MDP|&(IL23|(IL12&(IL2|IL15|IL18))))|(Th0_M&(LPS|MDP|PGN) &(IL12|IL23))|Th1|((CD8_NKG2D|NK_NKG2D)&(IEC_MICA_B|IEC_ULPB1_6))|(Th17&(PGN|LPS| $MDP))\;|\;((MACR\;|\;Th0)\&IL18\&IL12))\&!\;((IFNg\&({⋂}_{i=1}^{downreg\_cyt=4}{TGFb}^{t-i}\;|{⋂}_{i=1}^{downreg_{cyt}=4}{IL10}^{t-i}\;|\;Th2)$
*IL*23 = (*MACR* & *IL*1*b*) | ***DC***
$IL22=Th17|(NK\&((IL18\&IL12)\;|\;IL23))|CD4\_NKG2D|(((IL22\&Th0\&IL21)\&!({⋂}_{i=1}^{upreg\_cyt=3}{IL22}^{t-i}\&$ ${⋂}_{i=1}^{upreg\_cyt=3}{Th0}^{t-i}\&{⋂}_{i=1}^{upreg\_cyt=3}{IL21}^{t-i}))\&!\;TGFb)$
*IL*21 = ***Th*17** | ((***Th*0** & ***IL*6**) &! (***IL*4** | ***IFNg*** | ***TGFb***))
IL17=(Th17|(Th17_M&(LPS|MDP|PGN))|(CD4_NKG2D&(IEC_MICA_B|IEC_ULPB1_6)))&! $\left(({⋂}_{i=1}^{downreg\_cyt=4}{TGFb}^{t-i}\;|\;{⋂}_{i=1}^{downreg\_cyt=4}{IL13}^{t-i})\&IL17\right)$
*IL*10 = *Treg*|(*Th*2 &! *IL*23)|((*TLR*2 & *NFkB*) &! (*MACR* & *IFNg*)) | ((*MACR* & *LPS*) &! *IL*4) | (*DC* & *LPS*)
$Th17=((Th0\&(IL1b\;|\;IL23\;|\;IL6))\;|\;((Th17\&IL23)\&!({⋂}_{i=1}^{upreg\_cell=2}{Th17}^{t-i}\&{⋂}_{i=1}^{upreg\_cell=2}{Il23}^{t-i})))\&!$ $\left(({⋃}_{i=1}^{downreg\_cell=2}{TGFb}^{t-i}|\;{⋃}_{i=1}^{downreg\_cell=2}{IL12}^{t-i}\;|{⋃}_{i=1}^{downreg\_cell=2}{IL4}^{t-i}|{⋃}_{i=1}^{downreg\_cell=2}{IFNg}^{t-i}\;\right|$ ${⋃}_{i=1}^{downreg\_cell=2}{Treg}^{t-i})\&Th17)$
*Th*17_*M* = ((*Th*0_*M* & (*PGN* | *MDP* | *LPS*)) & ((*IL*1*b* & *IL*6) | *IL*23 | *IL*2)) | *Th*17_*M*
$Th1=(Th0\&((IL12\;|\;IFNg\;|\;IL18)\;|\;(DC\&IL12\&IL23\&LPS)))\&!\;((({⋃}_{i=1}^{downreg\_cell=2}{IL17}^{t-i}\&$ ${⋃}_{i=1}^{downreg\_cell=2}{IL12}^{t-i})\;|\;({⋃}_{i=1}^{downreg\_cell=2}{Treg}^{t-i}|\;{⋃}_{i=1}^{downreg\_cell=2}{Th2}^{t-i}|\;{⋃}_{i=1}^{downreg\_cell=2}{TGFb}^{t-i}\;|$ ${⋃}_{i=1}^{downreg\_cell=2}{IL10}^{t-i}|{⋃}_{i=1}^{downreg\_cell=2}{IL4}^{t-i}))\&Th1)$
$Th2=(Th0\&(IL10\;|((IL18\&IL4)\&!IL12))|\;((Th2\&IL4)\&!({⋂}_{i=1}^{upreg\_cell=2}{Th2}^{t-i}\&{⋂}_{i=1}^{upreg\_cell=2}{IL4}^{t-i})))\&!$ $\left(({⋃}_{i=1}^{downreg\_cell=2}{Treg}^{t-i}|\;{⋃}_{i=1}^{downreg\_cell=2}{IFNg}^{t-i}|\;{⋃}_{i=1}^{downreg\_cell=2}{TGFb}^{t-i})\&Th2\right)$
*IL*4 = *Th*2
*IL*15 = (*FIBROBLAST* & (*MDP* | *LPS* | *PGN*)) | (*MACR* & (*LPS* | *IFNg*))
$IL12=((((MACR\;|\;DC)\&(LPS\;|PGN)\&IFNg)\&!(IL12\&{⋃}_{i=1}^{downreg\_cyt=4}{TNFa}^{t-i}))\;|\;(DC\&IL1b)\;|$ $\left(IL12\&(IL13\;|\;IL4)))\&!(({⋃}_{i=1}^{downreg\_cyt=4}{TGFb}^{t-i}\;|\;{⋃}_{i=1}^{downreg\_cyt=4}{IL10}^{t-i})\&IL12\right)$
*IL*13 = *Th*2
$Treg=({⋂}_{i=1}^{{THR\_Th0\_Treg}_{=3}}{Th0}^{t-i}\&(TGFb\;|\;TLR2))\&!\;(({⋃}_{i=1}^{downreg\_cell=2}{IL6}^{t-i}|{⋃}_{i=1}^{downreg\_cell=2}{IL21}^{t-i}|$ ${⋃}_{i=1}^{downreg_{\_cell}=2}{IL23}^{t-i}|{⋃}_{i=1}^{downreg\_cell=2}{Th17}^{t-i}|\;{⋃}_{i=1}^{downreg\_cell=2}{IL22}^{t-i}|{⋃}_{i=1}^{downreg\_cell=2}{TNFa}^{t-i})\&Treg)$
$NK=(IL15\;|IL2\;|\;IL12\;|IL23|\;(IL18\&IL10))\&!\;({⋃}_{i=1}^{downreg_{cell}=2}{Treg}^{t-i}\&NK)$
$DEF=IL22\;|\;IL17\;|\;{⋂}_{i=1}^{THR\_NOD2\_DEF=3}{NOD2}^{t-i}$
*IL*2 = *Th*0 | (*Th*0_*M* & (*MDP* | *LPS* | *PGN*)) | *DC*
$MACR=(NFkB\;|\;((MACR\&(IFNg\;|IL15))\&!\;({⋂}_{i=1}^{upreg\_cell=2}{NFkB}^{t-i}\&({⋂}_{i=1}^{upreg\_cell=2}{IFNg}^{t-i}\;|$ ${⋂}_{i=1}^{upreg\_cell=2}{IL15}^{t-i}))))\&!\;({⋃}_{i=1}^{downreg\_cell=2}{IL10}^{t-i}\&MACR)$
$DC=NFkB\&!\;({⋃}_{i=1}^{downreg\_cell=2}{IL10}^{t-i}\&DC)$
$IEC\_MICA\_B=((LPS\;|\;MDP\;|\;PGN)\;|\;(IEC\_MICA\_B\&TNFa)\&!\;({⋂}_{i=1}^{upreg\_rec=2}{IEC\_MICA\_B}^{t-i}\&$ ${⋂}_{i=1}^{upreg\_rec=2}{TNFa}^{t-i}))\&!\;TGFb$
*IEC*_*ULPB*1_6 = *CD*8_*NKG*2*D* & (*LPS*|*MDP*|*PGN*)
$CD8\_NKG2D=(LPS\;|\;PGN\;|\;MDP)\&!(({⋂}_{i=1}^{THR\_LIGANDS\_NKG2D=3}{IEC\_MICA\_B}^{t-i}|{⋂}_{i=1}^{THR\_LIGANDS\_NKG2D=3}{IEC\_ULPB\_1\_6}^{t-i}$ $\left|\;({⋃}_{i=1}^{downreg\_cell=2}{IL21}^{t-i}\&{⋃}_{i=1}^{downreg\_cell=2}{IL2}^{t-i}))\&CD8\_NKG2D\right)$
$NK\_NKG2D=(LPS|PGN|MDP)\&!\;({⋃}_{i=1}^{downreg\_cell=2}{TGFb}^{t-i}|{⋂}_{i=1}^{TH{R\_}_{LIGANDS\_NKG2D}=3}{IEC\_MICA\_B}^{t-i}\;|$ ${⋂}_{i=1}^{THR\_LIGANDS\_NKG2D=3}{IEC\_ULPB\_1\_6}^{t-i}\;|({⋃}_{i=1}^{downreg\_cell=2}{IL21}^{t-i}\&{⋃}_{i=1}^{downreg\_cell=2}{IL12}^{t-i}))\&NK\_NKG2D)$
$CD4\_NKG2D=(LPS\;|\;PGN\;|\;MDP\;|\;(CD4\_NKG2D\&(IL15\;|\;TNFa))\&!\;({⋂}_{i=1}^{upreg\_rec=2}{CD4\_NKG2D}^{t-i}$ $\&({⋂}_{i=1}^{upreg\_rec=2}{IL15}^{t-i}|\;{⋂}_{i=1}^{upreg\_rec=2}{TNFa}^{t-i})))\&!\;(({⋃}_{i=1}^{downreg\_cell=2}{IL10}^{t-i}\;|$ ${⋂}_{i=1}^{TH{R\_}_{LIGANDS\_NKG2D}=3}{IEC\_MICA\_B}^{t-i}\;|\;{⋂}_{i=1}^{THR\_LIGANDS\_NKG2D=3}{IEC\_ULPB\_1\_6}^{t-i})\&CD4\_NKG2D))$
$FIBROBLAST=((MACR\&(IL4\;|\;IL13\;|\;TGFb))|IL2)\&!(({⋃}_{i=1}^{downreg\_cell=2}{IFNg}^{t-i}|{⋃}_{i=1}^{downreg\_cell=2}{IL12}^{t-i})$ &FIBROBLAST)
*PERFOR* = *NK* | *NK*_*NKG*2*D*
*GRANZB* = *CD*8_*NKG*2*D* | *NK* | *NK*_*NKG*2*D* | (*DC* &! (*LPS* | *PGN*))
**OUTPUT NODE**
*MMPs* = (*MACR* & *TNFa*) | (*FIBROBLAST* & (*IL*21 | *IL*17 | *IL*1*b* | *TNFa*))

Bold text within Boolean equations indicates that the information belongs to animal data

Table 2 contains the full set of BF that modulates the signal initialized by the antigens through the activation of different pattern recognition receptors (TLR2, TLR4 and NOD2 nodes) and the impact on the output node (MMPs) as the recipient of the antigen signal internal modulation. The nodes TNFα or IFNγ have the most complex pathways as can be seen in the corresponding Boolean equations (Table 2).

With the aim of making the network model more accessible to the community it has been uploaded to “The Cell Collective” [49,50] platform (https://www.cellcollective.org/#cb963d7f-75cb-4b2e-8987-0c7592a9c21d). In addition, the supporting information document S2 File provides the network model in text format ready for simulation in the R-based freely available package SPIDDOR [28] and an html tutorial as a guide to reproduce the results (S3 File).

### Perturbation analysis and clustering: Network robustness

**Fig 2:**
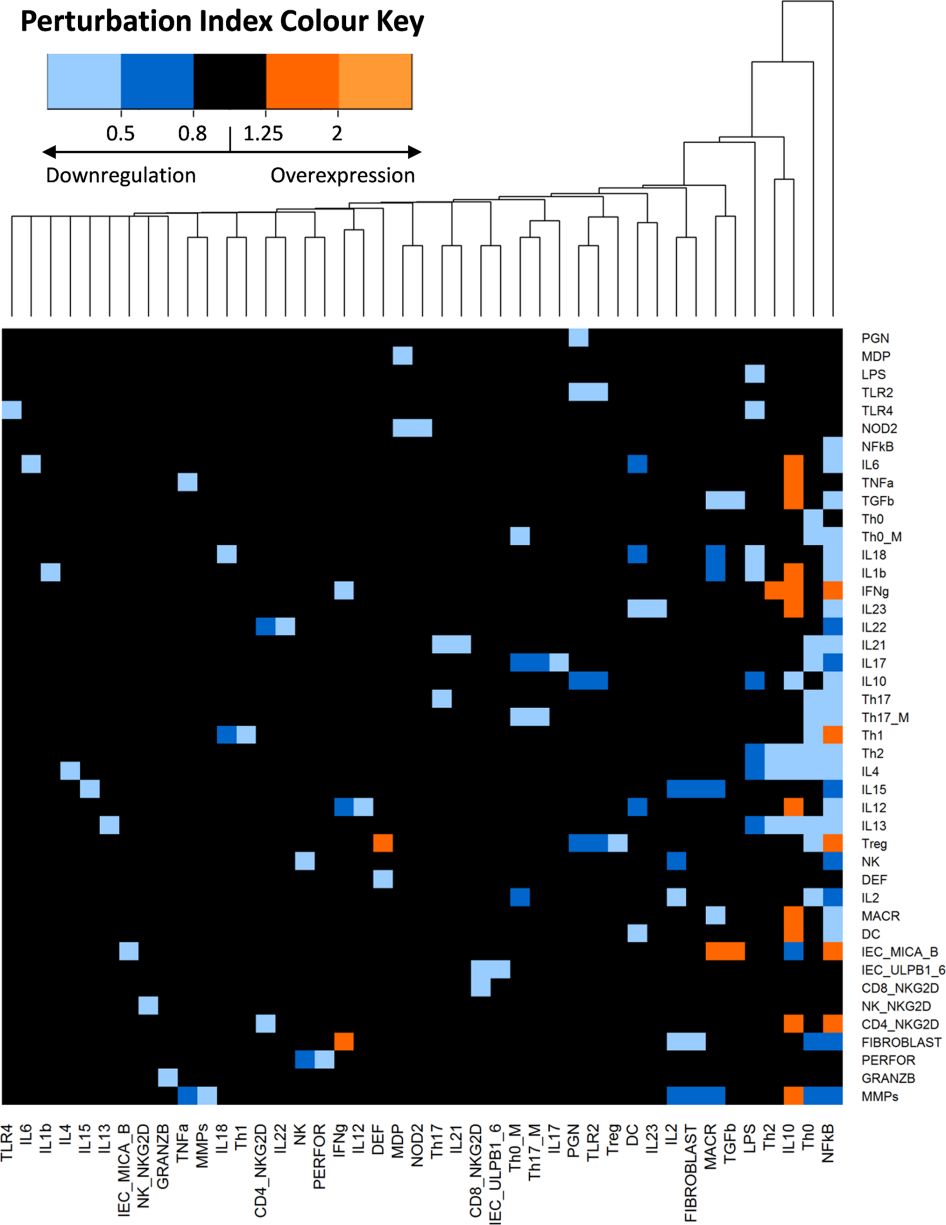
IBD network perturbation analysis and clustering. The heatmap indicates the effect of single blockage of each node (columns) in every network node (rows). The colour in each cell corresponds to the Perturbation Index (PI) of the nodes. When there is no change in the expression of the node, the cells of the heatmap would be black, having a value between 0.8 and 1.25 in their PIs. Otherwise, when the perturbation causes an overexpression in a node, the cell in the heatmap would be orange coloured, with PIs values greater than 1.25. On the contrary, a value of 0.8 or smaller, blue colour, indicates that the perturbation causes a downregulation of the node. The numeric scale in the legend represents different values of the nodes PI under different perturbations. Nodes that induce similar alterations are hierarchically clustered.

The results of the network perturbation analysis are presented in Fig 2. The heatmap shows the impact of a single blockage of each node in every network node. The results indicate that most node blockages did not trigger considerable changes, suggesting that the IBD network is robust [51]. Some perturbations led to a higher activation of the nodes, while down regulations were more common. The heatmap was combined with a hierarchical clustering grouping together the nodes that caused similar alterations. Knockout of the NFkß node appeared to be the most relevant alteration as it caused a reduction in expression of many of the nodes that were reported to be overexpressed in IBD patients. The knockout of the Th0 node (representing activated CD4+ T cells) also elicited a reduction in MMPs. The positive effects of the NFkß and Th0 node blockades on MMPs decreased expression, resembled some of the known mechanisms of action of glucocorticoids, inhibitors of T cell activation and proinflammatory cytokines, as well as potent suppressors of the effector function of monocyte-macrophage and fibroblastic activity, interfering with the NFκB inflammatory signal [52–54].

### Network accuracy and validation

#### Experimental and clinical information

**Fig 3:**
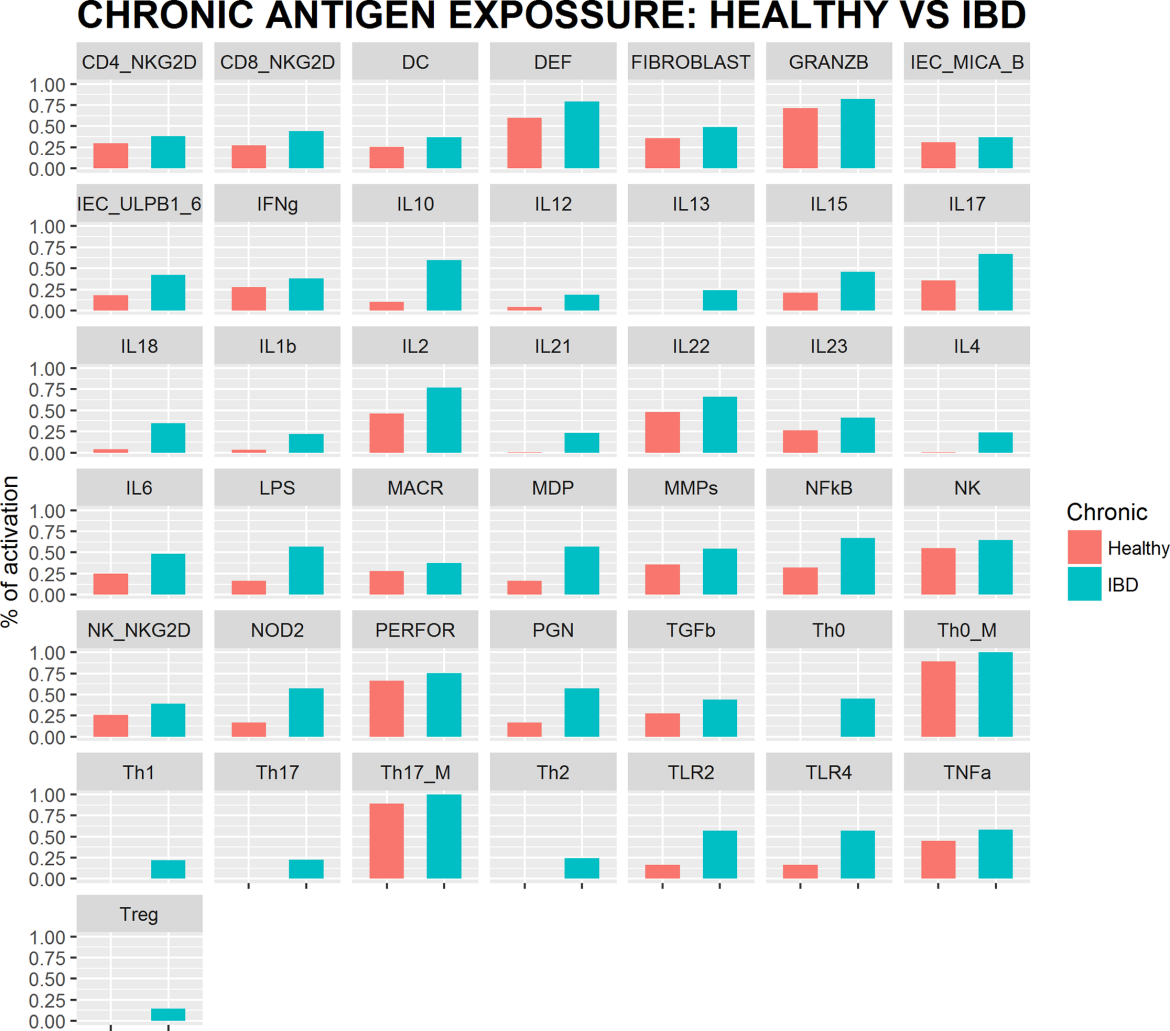
IBD network simulation results. Attractor state of every network node for healthy and IBD simulated individuals under chronic antigen exposure.

Simulations of chronic infection in IBD individuals show that the model reproduced satisfactorily experimental and clinical information (summarized in Table 3 and supporting information S3 Table). Fig 3 shows the results of the simulation for each network node after reaching the attractor state for virtual healthy and IBD subjects. In total, 31 upregulations in experimental studies were replicated with our simulations. Similarly, the 9 nodes reported as altered appeared upregulated in the simulations, and finally, the three nodes whose profiles were not known also proved to be upregulated.

**Table 3:** Expression of network nodes in IBD patients.

NODE	EXPRESSION	NODE	EXPRESSION	NODE	EXPRESSION	NODE	EXPRESSION
PGN MDP LPS	Altered	IL1b	Upregulated	Th2	Upregulated	DC	Downregulated in Blood-Upregulated in mucosa
TLR2	Upregulated	IFNg	Upregulated	IL4	Altered	IEC_MICA_B	Upregulated
TLR4	Upregulated	IL23	Upregulated	IL15	Upregulated	IEC_ULPB1_6	Upregulated
NOD2	Altered	IL22	Upregulated	IL12	Upregulated	CD8_NKG2D	Upregulated
NFkB	Altered	IL21	Upregulated	IL13	Upregulated	NK_NKG2D	Unknown
IL6 TNFa	Upregulated Upregulated	IL17	Upregulated	Treg	Downregulated in Blood-Upregulated in mucosa	CD4_NKG2D	Upregulated
TGFb	Upregulated	IL10	Upregulated	NK	Upregulated	FIBROBLAST	Upregulated
Th0	Unknown	Th17	Upregulated	DEF	Altered	MMPs	Upregulated
Th0_M	Upregulated	Th17_M	Upregulated	IL2	Upregulated	PERFOR	Altered
IL18	Upregulated	Th1	Altered	MACR	Unknown	GRANZB	Upregulated

A total of 31 nodes are reported as upregulated in IBD patients, 9 are reported to be altered (when different reports from literature are inconclusive or contradictory) and 3 nodes are unknown.

#### Clinical trials

**Fig 4:**
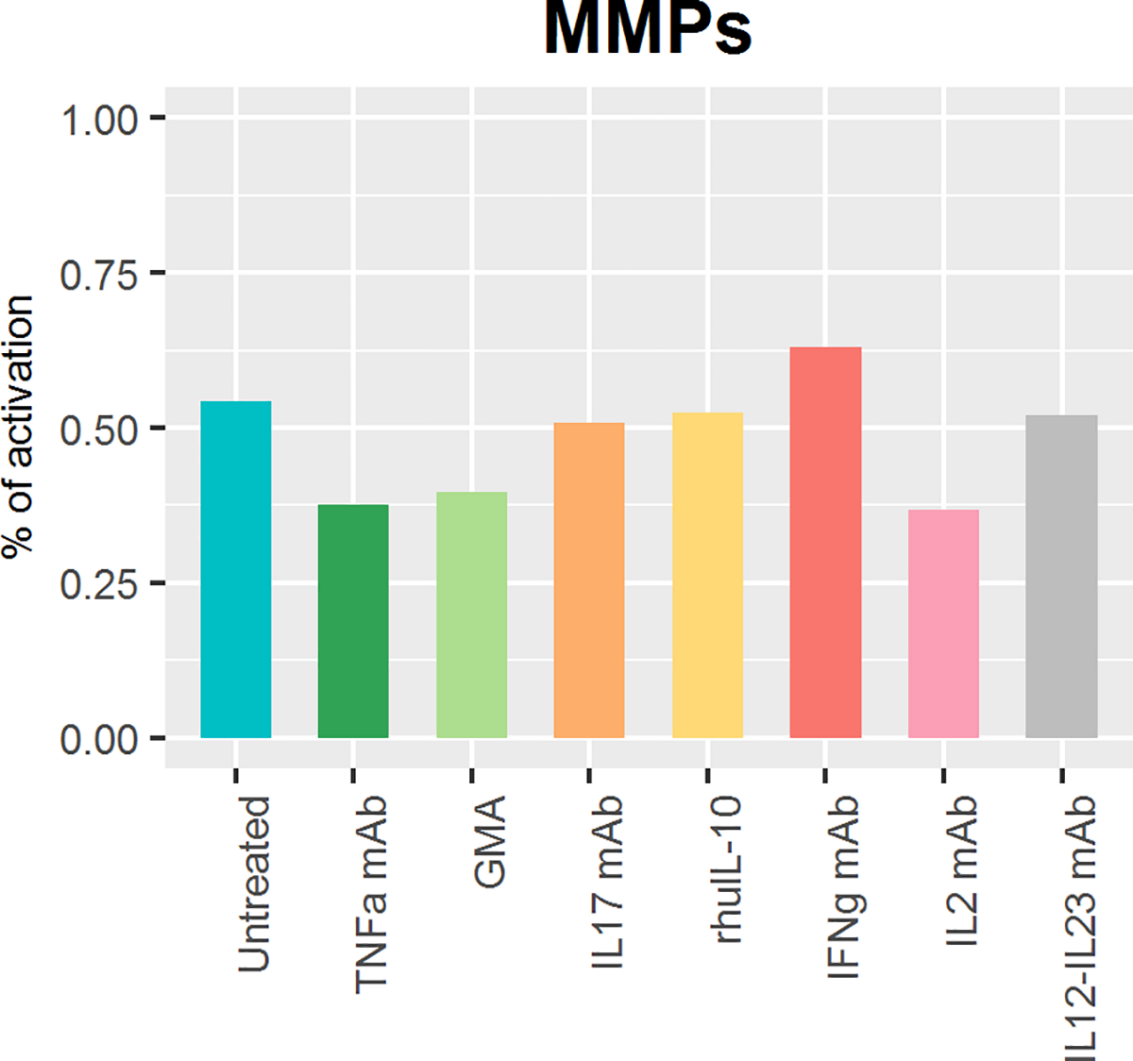
Comparison of MMPs expression after the simulation in IBD simulated individuals of different therapies. Simulated therapies: Anti-TNFα, GMA therapy (equivalent of knock out our MACR node), anti-IL17, human recombinant IL10 (rhulL-10), anti-IFNγ, anti-IL2 and anti-IL12-IL23. Comparing with untreated simulation, we can see a 30.7%, a 27.1%, a 31.9% and a 4.1% decrease in the MMPs expression simulating anti-TNFα, GMA therapy, anti-IL2 and anti-IL12-IL23 respectively. There is no major change in MMPs expression for the two which failed in clinical trials anti-IL17 (a 6.5% decrease) and human recombinant IL10 (a 3.2% decrease). Otherwise, anti-IFNγ therapy simulation shows an increase in MMPs expression of 16.0% compared to Untreated.

In our simulations, three drugs that have failed to prove clinical efficacy in clinical trials (anti-IL17, anti-IFNγ and rhuIL-10) also exhibited no benefit in the simulated surrogate for the disease score (Fig 4). Simulations with anti-TNFα, a biologic therapy approved for IBD, showed a decrease in the disease score. Simulations with anti-IL12-IL23, a recently approved therapy for IBD, showed a slight decrease in MMPs and anti-IL2 therapy simulation showed a decrease similar to anti-TNFα. In addition, the new promising therapy (GMA), equivalent to an anti-MACR in our model showed a decrease in MMPs similar to that for anti-TNFα.

## Discussion

In the current study, we present a Systems Pharmacology (SP) network model for IBD based on the main cells and proteins involved in the disease. Our analysis appears timely, as IBD has recently been attracting increasing attention [55–59]. We attempted to meet one of the major challenges in inflammatory bowel disease (IBD) which is the integration of IBD-related information to construct a predictive model. We are not the only ones following this line of research, as Lauren A Peters et al. have very recently performed a key driver analysis to identify the genes predicted to modulate network regulatory states associated with IBD [55]. Both analyses could be integrated in the future and inform our post-transcriptomic network with the key driver genes identified by Lauren A Peters et al. [55].

In comparison with the previous quantitative approaches for IBD [20,21,33,34], our model identified Naive CD4+ T Cells, Macrophages and Fibroblasts cells as relevant in IBD. Also, in addition to the six interleukins (TGFß, IL6, IL17, IL10, IL12 and IFNγ) considered by Mei et al. [33,34] our network involves 10 interleukins more which could represent possible IBD biomarkers [60]. The procedure to evaluate the potential role of the different components on the disease as plausible biomarkers, would be equal to the one described in section 4.5 (perturbation analysis and clustering), focussing on the changes in the output node.

In the validation of network models, robustness and practical applicability represent critical aspects. The fact that the information gathered from the literature was obtained under very different experimental designs/conditions/methodologies, represents a challenge with respect to validation. This led us to propose and adopt a novel strategy consisting of the comparison of the results of model-based virtual pathway simulations with those reported in the literature for IBD patients. Using this approach, we obtained a qualitative reproduction of IBD in which all the network elements that have been reported as upregulated in IBD patients appeared upregulated in our simulation results. The perturbation analysis of the network was performed by a single blockage in each node to analyse how that type of alteration propagates through the entire network reflecting the case of single polymorphisms, which represents the simplest case of IBD disease. Despite of the simplicity of this analysis, the results obtained from the model accuracy and validation procedures are encouraging. Results from the perturbation analysis indicate that the proposed network model is robust, as alteration in most nodes did not trigger considerable changes in the network [61].

Once validated and checked for robustness, the network was challenged to qualitatively reproduce the readouts of five different therapies reported in experimental and clinical studies. The outcome of this challenge was similar to the clinical output in IBD patients. By the simulation of TNFα or MACR knockout (simulating Granulocyte and Monocyte Apheresis), a decrease in MMPs node was observed, which is in line with therapy success in clinical practice by a decrease in Crohn's Disease Activity Index (CDAI) Score [42–46],[62–68]. On other hand, IL17 or IFNγ knockout or IL10 overexpression did not show major change in MMPs expression, suggested a failed therapy as was indeed found in clinical practice [69–72].

Surprisingly, the model shows that a knockout of IL2 leads to a reduction in MMPs similar to that of a knockout of TNFα, even when previous results of clinical trials with Basiliximab or Daclizumab (monoclonal antibodies that bind to the interleukin 2 receptor CD25) in Ulcerative Colitis have failed to show superiority to corticosteroids alone [73,74]. The mechanism of action of corticosteroids has not been fully described, yet it is known that corticosteroids cause diminished levels of IL2 mRNA [75,76]. Together with the rest of corticosteroid inhibitory mechanisms, this would be the reason why Basiliximab or Daclizumab do not show superiority to corticosteroids alone.

Among the potential applications the current network supports: (i) biomarker selection given that the cytokines TNFα, IL21, IL17 and IL1ß, which can be easily measured in peripheral plasma with different Enzyme-linked immunosorbent assay (ELISA) kits [77,78], are the model components directly related to MMPs activation, (ii) search for optimal combination therapy to overcome the high attrition rates in phase clinical trials with single therapies which are due mainly to lack of efficacy [79], and (iii) management of multiscale information such as the integration of proteomic gene expression data [55] accounting for IBD polymorphisms to anticipate responders and non-responders. With such a type of data able to correlate a genetic alteration with a decrease or an increase in protein expression, it would be possible to simulate specific genetic alteration by altering the protein expression. This would allow one of the limitations of the current network at the present time to be overcome with regard to the effects of Ustekinumab, a monoclonal antibody targeting free IL12 and IL23, which has been recently approved for moderately to severely active Crohn’s disease in adults who have failed to treatment with immunomodulators, or more than one TNFα blocker [80]. Simulation results based on the known mechanisms of Ustekinumab showed just a 4.1% decrease in tissue damage. On the other hand, when simulating TNFα blocker effects, tissue damage decreased by 30.6% even though a substantial percentage of patients showed poor control of the disease after treatment with anti-TNFα antibody [15,16].

We emphasize that the proposed network model is fully accessible which allows it to undergo immediate testing and further development. In that respect it should be noted that although our model intended to include information of human origin exclusively, some critical pathways had to be complemented with animal derived data (although in the current case the percentage of human supported pathways is greater than in previous computational models [20,81,82]), but we are aware of the wide differences in the immune system between species [83–85].

This study addresses the goals of systems pharmacology by effectively encompassing prior knowledge to generate a mechanistic and predictive understanding at the systems level for IBD. Semi-quantitative understanding at the network level is necessary prior to the generation of detailed quantitative models for within-host disease dynamics. The current IBD model and the companion literature summary archive will drive the development of a dynamic (i.e., ordinary differential equation driven) model involving meaningful parameters capable of simulating longitudinal data, and allowing model reduction as well the goal of parameter estimation during the clinical stages of the drug development process. In addition, our IBD network can be extended to other inflammatory diseases, as main pathways in the model are common to most inflammatory conditions [86,87], and the outputs of our nodes could also serve as inputs to broader-scale logic models; for example, incorporating structures from available logic models of some of our nodes such as fibroblast [61], IL1b or IL6 [88].

In summary, we present a network model for inflammatory bowel disease which is available and ready to be used and can cope with (multi-scale) model extensions. It is supported by a comprehensive repository summarizing the results of the most relevant literature in the field. This model proved to be promising for the *in silico* evaluation of potential therapeutic targets, the search for pathway specific biomarkers, the integration of polymorphisms for patient stratification, and can be reduced and transformed in quantitative model/s.

## Methods

### Literature search and data selection

The network model is based on an exhaustive bibliographic review focusing on the essential components of IBD, as previously performed by Ruiz-Cerdá et al., in their systems pharmacology approach for lupus erythematosus [23]. Our review included around 620 papers published between October 1984 and September 2017, yet the most common reviewed articles were from 2007 or later (76%). The search of the relevant literature was made through Medical Subject Headings (MeSH) terms using different search engines such as PubMed, clinicaltrials.gov or google scholar. MeSH terms were focused on the combination of keywords and free words including: (i) relevant network components (ej.”IL6”) involved in the pathogenesis of IBD, (ii) nodes that have been reported to be altered in IBD (ej. “IL6 AND IBD”) and (iii) nodes directly affecting the expression of the nodes selected in (i) and (ii) (ej. “DC AND IL6”). The internal nodes selection was made according to the reported upregulated components in IBD patients together with the nodes (immune system cells) which are necessary to link the upregulated nodes, which were established as internal nodes. Only original papers with a clear description of experimental conditions were considered to identify the relationships between the components of the biological network. Due to the reported differences between animal and human immunology [83–85], in only few cases were animal data considered to connect nodes of critical pathways when no human data were available.

### Annotation and system representation

Annotation was crucial to organize the available literature according to its relevance. S2 Table from supplementary information shows the way the information was organized for building the network. S2 Table includes every node definition and the relationships between the nodes. Annotation included the identification of the main elements (antigens, cytokines, cells, proteins, membrane receptors and ligands) of IBD disease.

The IBD model will be freely accessible to the public through the “The Cell Collective” repository https://cellcollective.org/#cb963d7f-75cb-4b2e-8987-0c7592a9c21d.

### Boolean network building and r implementation

The collection of qualitative relationships extracted from the literature was transformed into a logical model as described before by Ruiz-Cerdá et al. [23]. Logic networks capture the dynamics of their components, called nodes, after selected stimuli or initial conditions [89,90]https://paperpile.com/c/XvtklO/p0BRz+YiQ4q. In these models the relationships of activation or inhibition between nodes are described as combinations of the logic operators: AND, OR and NOT condensed in a mathematical expression called a Boolean function for each node. Positive and negative modulators, and thresholds as previously described by Ruiz-Cerdá et al.[23] and Irurzun-Arana et al. [28] were also considered to resemble better the biological system. Boolean network building and R implementation from S1 File gives a more detailed explanation of the modulators used in the model.

### Simulations

The set of combined Boolean functions for the IBD model was implemented SPIDDOR [28], using RStudio Version 0.99.442. Simulations with 25 repetitions over 5000 iterations were performed. According to preliminary experiments, these simulation conditions were required to achieve the steady state of the network called attractor [91–93]. An attractor can be a fixed-point if it composed of one state, a simple cycle if consists of more than one state that oscillates in a cycle or a complex attractor if a set of steady-states oscillate irregularly. In each simulation, a node can show two possible values in each iteration: 0 (deactivated) or 1 (activated). The percentage of activation of the output node (MMPs) calculated at the attractor state was used as the readout summary of the simulation exercises, as this group of proteins are directly associated with intestinal fibrosis and tissue damage in IBD [42–46].

Each node was updated asynchronously [94–96] according to its Boolean function that defines the dynamics of the system. Initial conditions are explained in detail in “Simulations” from S1 File.

### Perturbation analysis and clustering

Robustness can be defined as the system’s ability to function normally under stochastic perturbations [96]. The investigation of robustness in Boolean networks generally focuses on the dependence between robustness and network connectivity [97]. We performed a perturbation analysis in our IBD model to study robustness by simulating the effect of the single blockage of each node on every other node of the network [51]. This simulation was performed by using the *KO_matrix*.*f* function from SPIDDOR package with 24 repetitions over 999 iterations under asynchronous updating.

Results from the simulations described above were represented as heatmaps with dendrograms in which the number of rows and columns is equal to the number of nodes in the network (Fig 2). The colour in each cell of the heatmap corresponds to the Perturbation Index(PI) of the nodes, which is the probability ratio between the perturbed and the normal conditions as described by Irurzun-Arana et al. [28]. A hierarchical clustering method [98] was applied to further study which nodes cause similar alterations in the system.

### Network accuracy and validation

Accuracy was evaluated comparing the alterations reported in the literature for IBD patients with the simulations of chronic antigen exposure for IBD or healthy individuals.

A literature search of every node expression in IBD patients was performed, and the gathered information is condensed in S3 Table including three categories: up-, down-regulated, or altered, whether the levels in CD, UC or both (IBD) with respect to healthy volunteers are higher, lower, or inconclusive and/or contradictory, respectively.

For validation purposes, model simulations were compared against available results from clinical trials performed in IBD, CD or UC until the beginning of 2017 in https://www.clinicaltrials.gov/. All the molecules tested in clinical trials, whose mechanism of action is known and whose target were included in our network, were tested with the model. The network was evaluated comparing simulations and reported outcomes from clinical trials for six investigated molecules: anti-TNFα [62–65] and anti-IL12-IL23 [80], two monoclonal antibodies (mAb) approved for IBD disease, anti-IFNγ [69,70], anti-IL17 [72], anti-IL2 [73,74] and human recombinant IL10 (rhuIL-10) [71] which failed in clinical trials. Also a new promising therapy: Granulocyte and Monocyte Apheresis (GMA) [66–68] was tested. The reported CDAI (Crohn Disease Activity Index) was compared with the average expression of the MMPs output node in the attractor state.

## Supporting information

S1 Table Abbreviations. List of abbreviations.
(PDF)

S2 Table IBD Network Repository. Table of nodes and interactions supported by references.
(PDF)

S3 Table IBD_validation. Table of alterations in patients of IBD network nodes supported by references.
(PDF)

S1 File Supporting_Information_Methods. Document with detailed description of the methodology.
(DOCX)

S2 File IBD.txt. Text document with the Boolean functions written in SPIDDOR nomenclature for iBD simulation.
(TXT)

S3 File User_Guide_SPIDDOR_IBD.html. Html tutorial about how to reproduce the results from the present manuscript with the SPIDDOR package.
(HTML)
