# Supplementary material for: A systems pharmacology model for inflammatory bowel disease
Source: PLoS One. 2018 Mar 7;13(3):e0192949. doi: 10.1371/journal.pone.0192949 (PMC5841748; doi:10.1371/journal.pone.0192949)
Supplement: S1 Table — List of abbreviations. (PDF) [file pone.0192949.s001.pdf]

Table_S1: Abbreviations

ABBREVIATION	MEANING
BF	Boolean Function
CD	Crohn's disease
CD4_NKG2D	NKG2D receptor of CD4+ T cells
CD8_NKG2D	NKG2D receptor of CD8+ T cells
DC	Dendritic cell
DEF	Defensin
FIBROBLAST	Fibroblast
GRANZB	Granzyme B
IBD	Inflammatory Bowel Disease
IEC_MICA_B	MHC class I-chain related gene A and B of intestinal epithelial cells
IEC_ULPB1_6	NKG2D ligands
IFNg	Interferon gamma (IFNγ)
IL1b	Interleukin 1 beta
IL10	Interleukin 10
IL12	Interleukin 12
IL13	Interleukin 13
IL15	Interleukin 15
IL18	Interleukin 18
IL17	Interleukin 17
IL21	Interleukin 2
IL22	Interleukin 22
IL23	Interleukin 23
IL2	Interleukin 2
IL4	Interleukin 4
IL6	Interleukin 6
LPS	Lipopolysaccharide
MACR	Macrophage or Monocyte
MDP	Muramyl-dipeptide

MMPs	Matrix Metalloproteinases
NFκB	Nuclear factor Kb
NOD2	Nucleotide-binding oligomerization domain
NK	Natural killer cells
NK_NKG2D	NKG2D receptor of Natural Killer cells
PERFOR	Perforin
PGN	Peptidoglycan
SP	Systems Pharmacology
TGFb	Transforming growth factor beta
Th0	Activated CD4+ T cell
Th0_M	Activated CD4+ MemoryT cell
Th1	CD4+ T helper 1 cell
Th17	CD4+ T helper 17 cell
Th17_M	CD4+ T Memory helper 17 cell
Th2	CD4+ T helper 2 cell
TLR2	Toll-Like Receptor 2
TLR4	Toll-Like Receptor 4
TNFa	Tumor necrosis factor-alpha (TNFα)
Treg	T regulatory cell
UC	Ulcerative colitis
