# Supplementary material for: A systems pharmacology model for inflammatory bowel disease
Source: PLoS One. 2018 Mar 7;13(3):e0192949. doi: 10.1371/journal.pone.0192949 (PMC5841748; doi:10.1371/journal.pone.0192949)
Supplement: S2 Table — Table of nodes and interactions supported by references. (PDF) [file pone.0192949.s002.pdf]

S2 Table. IBD network repository. The selected nodes are reported in the column named "Receptor Node". When the "Receptor node" cell is pink coloured, it means that the particular node is reported as altered in IBD. The description of the functional relationship between receptor nodes and the nodes interacting with them is listed in the columns named "Effector Node" and "Action" respectively, summarizing the information with a brief fragment or a graph from the literature. The explanation is followed by the "Reference" column. A code colour for the references is used to remark the quality of the reference. On the one hand, green colour is used for trustable, clear and supported by experiments references. On the other hand, yellow colour is used for reviews or articles containing information from animal data. When a node/relation is in cell with orange colour, means that they are not included in final model.

1	PGN	23	Th1
2	MDP	24	Th2
3	LPS	25	IL4
4	TLR2	26	IL15
5	TLR4	27	IL12
6	NOD2	28	IL13
7	NFκB	29	Treg
8	IL6	30	DEF
9	TNFa	31	NK
10	TGFb	32	IL2
11	Th0	33	MACR
12	Th0_M	34	DC
13	IL18	35	IEC_MICA-B
14	IL1b	36	IEC_ULPB1-6
15	IFNg	37	CD8_NKG2D
16	IL23	38	NK_NKG2D
17	IL22	39	CD4_NKG2D
18	IL21	40	FIBROBLAST
19	IL17	41	MMPs
20	IL10	42	PERFOR
21	Th17	43	GRANZB
22	Th17_M		

COLOUR	MEANING
(pink)	Altered node in IBD
(orange)	Not included in the function
(green)	Reference is clear and it is supported by experiments
(yellow)	Reference is a review or is supported by experiments performed in mouse

Nº	Receptor Node	Effector Node	Action	Reference
1-3	PGN<br><br>MDP<br><br>LPS	NOT (PERFOR OR GRANZ)	"**NK cells kill their target cells** through two major pathways, both requiring close contact between NK cells and the target cells. In the first pathway, **cytoplasmic granule toxins including perforins and granzymes are secreted by exocytosis and together induce apoptosis of the target cells**. The second pathway involves the **engagement of death receptors in target cells by their cognate ligands in NK cells**, resulting in classical caspase-dependent apoptosis [8]."<br><br>"**Intestinal NK cells are phenotypically distinct** from their counterparts in the blood and resemble "helper" NK cells, which have potentially important functions both in **promoting anti pathogen response**s and in the **maintenance of intestinal epithelium** [13]"<br><br>**"Relative reduction in CD56+ NK cells** may conversely **have an impact on intestinal epithelial repair processes in the gastrointestinal tract** [18, 19]**.**"<br>"These data indicate that NKp46+CD3− NK cells in **gut mucosa** are **distinct from conventional NK cells characteristic of IFN-γ production and cytotoxicity** and may be involved in intestinal epithelial homeostasis and prevention of intestinal inflammation"	[1]
			[8] "we show in mice…"	[2]
			[13] Previous studies have reported NK cell-mediated natural cytotoxicity in lamina propria lymphocyte preparations (20–22)<br>• **NKp46+ RORgd− intestinal NK cells: NK cells→** Produce IFNg, GranzB, PERF. Cytolytic<br>• **IL-22-producing NKp46+ RORgd+ LTi-derived cells: LTiNKcells→** Produce IL22. No cytolytic	[3]
			[19]: "While **one subset** of **lamina propria NK cells** may act to promote pathogen-specific immune responses to enhance the T helper type 1 response via the **production of IFN-g, another subset may be involved in innate immunity and have a 'repair' function by regenerating the intestinal epithelial barrier.** The distinct subset of CD56+ CD11c- cells described here may be involved in the latter function, to limit the already established inflammation in IBD."	[4]
			"Membrane fusion and degranulation results in the release of **effector proteins (e.g. perforin and granzymes)** into the intracellular synaptic space and the appearance of secretory lysosome associated membrane proteins on the cell surface [21, 27, 28]"	[5]

			“**NK cells** and supposedly is associated with the same lysosomal compartment that is used for storage and **release of perforin and granzymes** [8, 15, 29].”	
			“Truncated BID (tBID) translocates to the mitochondrial membrane and plays a critical role during the induction of apoptosis [45]. Thus, **granzyme B might be considered a major cytotoxic effector released from secretory lysosomes**.”<br><br>**“perforin forms pores similar to the terminal complement components to then enable the protease granzyme B to enter the target cell and to initiate apoptosis** [49,55,57–60]”<br>**“perforin at sublytic concentrations did not form pores but still enabled granzyme B to enter the target cell** [61].”<br><br>“**binding of granzyme B** to **target cell membranes** is **charge-dependent but perforin-independent**” granzyme is transported to an endosomal compartment within the target cell, again in a perforin-independent manner. Interestingly, also in this scenario, perforin acts as a cofactor for the induction of apoptosis by an unknown mechanism”	[5]
			“I**BD-related inflammation is marked by mucosa**l **accumulation of cytotoxic, GrB**-expressing CD19+ and IgA+ cells, **suggesting a role for these cells in IBD-associated epithelial damage”**<br><br>**“GrB-induced target cell death** by NK cells **and CD8+ lymphocytes is strictly dependent on perforin (34, 35).** However, it has been demonstrated that **GrB** may enter the target cell by **a perforin-independent pathway (36)“**<br><br>**“**The spectrum of functions exhibited by GrB is much more diverse than originally thought and includes **cytokine-like effects, immunosuppressive effects, ag processing, matrix degradation, and cleavage of autoantigens.”**	[6]
			“Cytotoxic lymphocytes include cytotoxic T lymphocytes (CTLs) and natural killer **(NK) cells**. Despite substantial differences in how these two cell types are activated and how they recognize their targets, the key pathways that mediate target cell death are conserved. **Once conjugated to a target cell**, the **cytotoxic secretory granules traffic to the immunological synapse and release** a cargo of deadly proteins **including perforin granzymes** (FIG. 1) and granulysin."	[7]
		NOT DEF	“Our group was able to show that patients with ileal CD have a re**duced expression of TCF4 and also a reduced expression of HD5 and HD6 (***a defensins*)*,* resulting in a lower antibacterial activity of intestinal mucosal extracts from patients with ileal CD, implicating a functional relevance of these observations.”	[8]

			columnar cells produce several **antimicrobial peptides, which are known as defensins** [33].<br><br>**defensins as broad spectrum of microbicidal activity** against Gram-negative and Gram-positive bacteria, fungi, viruses and protozoa [34].They are capable of forming pores in the microbial membranes leading to disruption of the bacteria [35].	[9]
			“**The alpha-defensins are lacking owing to several Paneth cell defects**. In **colonic CD**, the **expression of beta-defensins is inadequate**. This may be related to downregulation of the transcription factor peroxisome proliferator-activated receptor-gamma and in some cohorts is associated with a reduced HBD2 gene”<br>“The **lack of alpha-defensins in ileal CD was confirmed by two other research groups** [44, 45].<br><br>“This negatively charged biochemical and physical barrier lines the entire gastrointestinal tract to prevent pathogens from coming too close to the epithelium [39]. Thus, **the entire intestinal epithelium, covered by a shield of cationic defensins which are fixed in the negatively charged mucus layer [40], forms the innate barrier of the gut”**<br>**“In IBD, defects in this intestinal shield enable the luminal microorganisms to attack the epithelium”**<br><br>**UC:** “The mucus layer, which protects the host from the enormous amounts of luminal microbes, is defective. This is accompanied by an insufficient differentiation from intestinal stem cells towards goblet cells. All these disturbances in the gut barrier shift the balance from epithelial defence towards bacterial offence. “<br><br>**“Beta-defensins and other defensin-like peptides** are sufficiently induced in UC under inflammatory conditions, and moreover, the **antimicrobial activity of the inflamed UC mucosa is significantly higher compared with the inflamed CD mucosa** [61, 62, 68]. **Therefore, it seems that defensin synthesis and activity are not disturbed in UC**.”<br><br>“Overall, defective goblet cell differentiation might explain the deficient mucus layer in UC. **The sufficiently secreted defensins are not retained by the defective mucus layer, thus allowing bacteria to pass through the epithelium and induce inflammation** (Fig.3) [51]”	[9]
		**Alteration:**<br>“Local changes in tissue integrity associated with focal areas of inflammation may result in the selection of a dysbiotic bacterial community associated with the propagation of a disease phenotype.”<br><br>“A health-associated microbiota plays an important role in the defense against infectious pathogens, and mechanisms associated with colonization resistance		[10]

facilitate pathogen clearance from the intestinal tract. Phenomena that alter microbiota composition, such as host inflammation or antibiotic treatment, can be exploited by obligate or opportunistic pathogens, including **Salmonella enterica serovar Typhimurium and Clostridium difficile**. (Keeney and Finlay, 2011; Ng et al., 2013), **Enterococcus ssp**. (Kinnebrew et al., 2010; Ocvirk et al., 2015) and **Escherichia ssp**. (Patwa et al., 2011; Tchaptchet et al., 2013), which benefit from the resulting pertur-bation of the microbiota (Lupp et al., 2007; Stecher et al., 2007; Hoffmann et al., 2009; Hajishengallis et al., 2012; Lawley et al., 2012). Once dysbiosis is established, pathogens can rapidly out-compete commensals due to factors in their genomes (e.g. those encoding bacterial toxins, antimicrobial resistance, adhesion fac-tors) that confer greater resistance to host defense mechanisms (e.g. antimicrobial peptides, reactive oxygen species and phago-cyte killing), and better utilization of the gut nutrient environment (Raffatellu et al., 2009; Winter et al., 2010; Keeney and Finlay, 2011; Rivera-Chavez and Baumler, 2015)."

"It has been shown that **infectious bacteria, such as adherent-invasive Escherichia coli (AIEC) and Mycobacterium avium subsp. paratuberculosis,** are fre-quently associated with the pathogenesis of inflammation in CD patients (Rhodes, 2007; Behr and Kapur, 2008; Barnich et al., 2013; Chassaing et al., 2014, 2015; Vazeille et al., 2016). **This observation leads to the tempting speculation that transient infection with pathogens cause changes in the microbial environment of the sus-ceptible host, and thereby triggers the development of chronic inflammation once the pathogen has been cleared**. However, it is still debated whether bacterial infection is primary or secondary to the underlying immune dysregulation in CD patients (Glasser et al., 2001)."

NK IBD:

- **NKp46+ RORgd– intestinal NK cells: NK cells→** Produce IFNg, GRANZB, PERFOR. Cytolytic
- **IL-22-producing NKp46+ RORgd+ LTi-derived cells: LTiNKcells→** Produce IL22. Are not cytolytic.

DEF **CD**: The **expression of beta-defensins is inadequate**. This may be related to downregulation of the transcription factor peroxisome proliferator-activated...// The **lack of alpha-defensins in ileal CD was confirmed by two other research groups**

DEF **UC: It seems that defensin synthesis and activity are not disturbed in UC.** The sufficiently secreted defensins are not retained by the defective mucus layer, thus allowing bacteria to pass through the epithelium and induce inflammation.

- alpha-defensins is reduced in ileal CD
- beta-defensins is decreased in colonic CD
- mucus layer is deficient in UC.

- ACUTE INFECTION:
  - **PGN** = PGN **AND NOT** (PERFOR **OR** GRANZB **OR** DEF)
  - **MDP** = PGN **AND NOT** (PERFOR **OR** GRANZB **OR** DEF)
  - **LPS** = PGN **AND NOT** (PERFOR **OR** GRANZB **OR** DEF)

- CHRONIC INFECTION:
  - **PGN** = **NOT** (PERFOR **OR** GRANZB **OR** DEF)
  - **MDP** = **NOT** (PERFOR **OR** GRANZB **OR** DEF)
  - **LPS** = **NOT** (PERFOR **OR** GRANZB **OR** DEF)

<table>
<tr><td rowspan="3">4</td><td rowspan="3">TLR2

Overexpressed in UC and CD [11,12]</td><td rowspan="2">PGN</td><td>Muramyl dipeptide was recognized by NOD2 but not by TLR2
(Do not recognize MDP)</td><td>[13]</td></tr>
<tr><td>“TLR2, one member of the TLR family, recognizes conserved molecular patterns associated with both Gram-negative and Gram-positive bacteria, including lipopeptides / lipoproteins, lipoteichoic acid, zymosan, and components of **peptidoglycan”**

“As examples; **TLR2 recognizes peptidoglycan** (a bacterial cell wall component) and constituents of gram-negative bacteria, mycobacteria and fungi“</td><td>[14–16]</td></tr>
<tr><td colspan="2">“the expression of total TLR2 and sTLR2 in intestinal mucosa is increased in patients with **UC**.”
“patients with a higher activity score have the lowest sTLR2 levels”</td><td>[11]</td></tr>
<tr><td colspan="5">**TLR2** = PGN</td></tr>
<tr><td rowspan="2">5</td><td rowspan="2">TLR4

upregulated in CD and UC. [17]</td><td rowspan="2">LPS</td><td>“**TLR4** interacts with **lipopolysaccharide (LPS),** resulting in the recruitment of the adapter molecule MyD88, phosphorylation of the IL-1 receptor-associated kinase (IRAK) followed by tumor necro-receptor-associated kinase (IRAK) followed by tumor necrosis factor (TNF) receptor-a”</td><td>[17]</td></tr>
<tr><td>“**TLR4** recognizes bacterial lipopolysaccharides **(LPS)**”</td><td>[16]</td></tr>
<tr><td colspan="5">**TLR4** = LPS</td></tr>
<tr><td rowspan="2">6</td><td rowspan="2">NOD2

Altered
[18]
[8]
[19][20]</td><td colspan="2">NOD2 ARE PART OF THE INFLAMMASOME:

Inflammasomes are formed by:
● an **adaptor protein**
● an **inflammatory caspase.**
● a **sensor protein**. Which are:
○ **PRRs** that act as scaffolds. Could be a member of the NLR:
■ NLR family include:
● the NOD (NOD1, **NOD2**, NOD3/NLRC3, NOD4/NLRC5, NOD5/NLRX1, CIITA), NLRP or NALP (NLRP 1–14) and IPAF (NLRC4 and NAIP) subfamilies.
■ or a pyrin (PYD) and HIN domain-containing protein **(PYHIN) family.**</td><td>[21]</td></tr>
<tr><td>MDP</td><td>“Peptidoglycan **(PGN), as a major component of bacterial cell walls in both Gram-positive and Gram-negative bacteria,** 5 is known **as a potent immunostimulator**. Nod1 and **Nod2**, founding members of the NLR protein family, **have been shown to recognize bacterial PGN fragments** (muro-peptides) and regulate innate and adaptive immune responses by activating transcription factors including NF-κB.**6**”</td><td>[22]</td></tr>
</table>

			“**The minimum ligand for Nod2, on the other hand, is MDP** (muramyl dipeptide: MurNAc-L-Ala-D-isoGln).8,9”	
			6 “NOD1 and NOD2 sense intracellular PGN fragments from bacteria.”<br><br>“Additional work revealed that **muramyl dipeptide (MDP), a PGN motif widely distributed among both Gram+ve and Gram−ve bacteria, is sufficient to trigger NOD2 activity** (Girardin et al., 2003b; Inohara et al., 2003).”	[23]
			“**Muramyl dipeptide** was recognized by **NOD2** but not by TLR2”	[13]
			“**NOD2 signalin**g is intimately entwined with TLR signaling such that **NOD2 recognition of MDP** tolerizes cells to further TLR2 or TLR4 activation”	[24]
		**Comment:** NOD2: Is in DC, stromal cells, MACR<br><br>“**NOD2 plays a crucial role in regulating intestinal homeostasis.** By **sensing microbiota-derived PGN fragments**, NOD2 activates NF-κB, which, in turn, leads to the production of antimicrobial peptides (AMPs) in Paneth cells (PC) that provide a barrier between the microorganisms and the epithelial layer. **Activation of NOD2 in dendritic cells (DCs)** leads to production of the interleukin-23 (IL-23) thus promoting an early mucosal T helper 17 (Th17) cell response that enhances barrier protection by inducing IL-22 and regenerating islet-derived protein IIIγ (REGIIIγ). **NOD2 activation in stromal cells** also promotes CC-ligand 2 (CCL2)-mediated recruitment of inflammatory monocytes (Mo) to the intestine. **Interaction between NOD2 and ATG16L1 promotes autophagosome formation in intestinal epithelial cells (IEC) and intraepithelial bacterial clearance. Crohn’s disease-associated NOD2 variants perturb many aspects of immune homeostasis including reduced MDP sensing in both macrophages (Mac) and DCs, impaired anti-microbial responses in Paneth cells, and altered autophagy leading to defective barrier function and/or bacterial clearance**. **These alterations along with the development of dysbiosis may lead to enhanced mucosal adherence and translocation of bacteria**.”<br><br>“**the activation of NOD2 by intracellular pathogens induces the formation of an autophagosome,** which is mediated by ATG16L1.”<br><br>CARD is altered in IBD [25]). CARD<br><br>“**Presence of a CARD15 mutation** -which has been shown to be associated with increased Crohn’s disease susceptibility- imbalances TLR2-mediated NFkB activation in antigen presenting cells, thus leading to mucosal inflammation through exaggerated interferon-g , IL-12, and IL-23 production, 29 which in turn **may further suppress mucosal TLR2-mediated anti-inflammatory IL-10 production via paracrine loops.** 30” [14]<br><br>**Comment:** MDP belongs to PGN. NOD2 apparently recognizes MDP. In some		[23]

<table>
<tr><td></td><td></td><td colspan="2">references [22] is reported that NOD2 recognizes PGN as well. We assume that is because both (PGN and MDP) are part of the same structure in G+ and G-, and NOD2 recognizes just MDP. But to initiate a simulation, we should not simulate PGN and MDP separately.</td><td></td></tr>
<tr><td colspan="5">NOD2 = MDP</td></tr>
<tr><td rowspan="4"></td><td rowspan="4">MAPK</td><td rowspan="2">TLR2 OR TLR4</td><td>“As an example, TLR4 interacts with lipopolysaccharide (LPS), resulting in the recruitment of the adapter molecule MyD88, phosphorylation of the IL-1 receptor-associated kinase (IRAK) followed by tumor necrosis factor (TNF) receptor-a associated factor 6 (TRAF6). Most TLRs use MyD88 as an adapter molecule, although other adapter molecules such as TRIF are utilized by TLR3. Recruitment of TRAF6 leads to the phosphorylation of Ikb- kinases and release of NFkB as well as activation of the mitogen activated protein kinase (MAPK) pathway. “</td><td>[26] Not well explained</td></tr>
<tr><td>“TLR4 moves from the plasma membrane to the endosomes in order to switch signalling from MYD88 to TRIF. Engagement of the signalling adaptor molecules stimulates downstream signalling pathways that involve interactions between IL-1R-associated kinases (IRAKs) and the adaptor molecules TNF receptor-associated factors (TRAFs), and that lead to the activation of the mitogen-activated protein kinases (MAPKs)”</td><td>[27]</td></tr>
<tr><td>IL22</td><td>“IL-22 activates ERK and SAPK/JNK MAP kinases in IEC”</td><td>[28]</td></tr>
<tr><td>NOD2</td><td>“NOD2 interacts directly with intracellular bacterial PGN fragments containing the MDP motif. Ligand recognition relieves intramolecular autoinhibitory interactions, leading to NOD oligomerization. Recruitment of the downstream serine/threonine kinase (S/T kinase) RIPK2 occurs through CARD -CARDinteractions. Subsequent activation of the NF-kB and MAPK pathways results in the transcriptional upregulation of proinflammatory and host-defense genes.”</td><td>[23,26]</td></tr>
<tr><td colspan="5">MAPK = TLR2 OR NOD2 OR TLR4 OR IL22</td></tr>
<tr><td>7</td><td>NFkB

TRAF3IP2 is altered in IBD: [25]</td><td colspan="2">“Nuclear factor kappa-light-chain-enhancer of activated B cells (NF-kB) is a family of transcription factors implicated in diverse biological processes, including inflammation, apoptosis, proliferation, and development. Based on the components of the signaling cascade, the NF-kB signaling pathway can be categorized as either a canonical or noncanonical pathway.”

● canonical NF-kB signaling pathway is regarded as the central regulator of the inflammatory response in normal physiology.
● Noncanonical NF-kB signaling is regulated at multiple levels. Under normal conditions, NF-kB-inducing kinase (NIK) proteins are kept at low levels and, therefore, NIK stabilization is a critical step for the activation of the pathway.</td><td>[29]</td></tr>
</table>

		TLR2 OR TLR4	"As an example, **TLR4** interacts with lipopolysaccharide **(LPS)**, resulting in the recruitment of the adapter molecule **MyD88**, phosphorylation of the IL-1 receptor-associated kinase **(IRAK)** followed by tumor necrosis factor (TNF) receptor-a associated factor 6 **(TRAF6)**. Most TLRs use MyD88 as an adapter molecule, although other adapter molecules such as TRIF are utilized by TLR3. Recruitment of TRAF6 leads to the phosphorylation of Ikb- kinases and release of **NFkB** as well as activation of the mitogen activated protein kinase **(MAPK)** pathway."<br>**LPS → TLR4 → MyD88 → IRAK → TRAF6 → NFkB** and **MAPK**	[26]
			"Downstream cascades **involve** recruitment of the cytoplasmic adaptor proteins Mal and MyD88 to the **TLR2 complex,** (22) which results in **activation of nuclear factor- k B (NF- k B)** and subsequent production of many cytokines and chemokines."	[14]
			"Two important families of transcription factors that are activated downstream of TLR signalling are nuclear factor-κB (NF-κB) and the interferon-regulatory factors (IRFs)"	[27]
		NOD2	"**NOD2 interacts directly with intracellular bacterial PGN** fragments containing the MDP motif. Ligand recognition relieves intramolecular autoinhibitory interactions, leading to NOD oligomerization. Recruitment of the downstream serine/threonine kinase (S/T kinase) RIPK2 occurs through **CARD interactions. Subsequent activation of the NF-kB and MAPK** pathways results in the transcriptional upregulation of proinflammatory and host-defense genes."<br><br>**MDP → NOD2 → CARD → RIPK2 → NFkB** and **MAPK**	[23]
		"Under normal physiological conditions, **noncanonical Nuclear factor kappa- light-chain-enhancer of activated B cells (NF-kB**) signaling is mainly involved in lymphoid organogenesis as well as B cell survival and maintenance... Aberrant activation of this pathway is commonly observed in human malignancies, ... **In particular, TNF receptor- associated factor 3 (TRAF3) mutations** …"		[29]
		**TRAF3IP2** is altered in IBD		[25]
		**TRAF3IP2→** NFkB activator. Is altered.		http://www.genecards.org/cgi-bin/carddisp.pl?gene=TRAF3IP2
		"TLRs activate the transcription factors NFKB, AP-1, and IRF to induce the expression of inflammatory cytokines and type I interferons."<br><br>"NFKB and AP-1 act primarily to induce the expression of pro-inflammatory cytokines and chemotactic" Fig page 91.:<br><br>**Comments:** Node located inside the cells that present TLRs		IBJ I (INTRODUCTION TO IMMUNOBIOLOGY AND Basic Concepts in Immunology)

NFkB = TLR2 OR NOD2 OR TLR4				
8	IL6<br><br>**Upregulate d** in CD and UC<br>[30]	NFkB AND NOT (IL4 OR IL10)	"The dominant **MyD88** dependent pathway leads to the phosphorylation of **IkB-** kinases which releases the **NF-kB** transcription factor sequestered in the cytosol, and at the same time activates the mitogen activated protein kinase **(MAPK)** pathway causing nuclear translocation and the activation of inflammatory cytokines such as TNF-α, IL-1, **IL-6** and IL- 18". Ref (43,81)<br><br>**MyD88 → IkB-kinase→ NFkB** and **MAPK → IL6**	[16]
			43: Do not mention IL6.	[26]
			81: Do not mention IL6.	[31]
			"NFkB is likely to play a role in the expression of genes for other cytokines such as IL-6, TNF-a, and lymphotoxin. Moreover, **NF-KB can itself be induced by TNF-a and IL-1** (Osborn et al., 1989)"	[32]
			(Osborn et al., 1989): "TNF-a and IL-1 are These cytokines, both products of activated macrophages, thus represent specific physiologic activators of NF-KB and the HIV enhancer."<br>Nothing about IL-6.	[33]
			**AND NOT IL4: "Suppression of IL-6 production in monocytes was associated** with **decreased nuclear NF-KB activity.** These findings demonstrate that **IL-4 inhibits IL-6 production in a tissue-specific manner**, and suggest a potential mechanism by which IL-4 may regulate the expression of multiple cytokines."<br><br>**Figure3 and Figure 4**	[34]
			**AND NOT IL10: "**We demonstrate here that **IL10, added to monocytes,** activated by interferon, LPS, or combinations of LPS and IFN at the onset of the cultures, **strongly inhibited the production of** IL1a IL1b, **il6,** il8, TNFa, GM-CSF, and G-CSF **at the transcriptional level (NF-kB)**."<br>**Table 1.**	[35]
		Th17 AND IL23	"**IL-6 were the most specific for TH-17**".<br>"**IL-6 production** was mostly **dependent on TGF-b and IL-23**, a regulation that is more closely related to that of IL-17".<br><br>**Comment:** **Th17 AND IL23** instead of **Th17 AND IL23 AND TGFb** because TGFb inhibit Th17 in its boolean function and IL6 would not be activated. The graph from the experiment shows how IL6 is also secreted by Th17 in the presence of IL23, but in a less extent.	[36]
		MACR AND	"**Wild-type macrophages** showed **dose-dependent production of**	[37]

		PGN	**IL-6**, TNFa and IL-12 **in response to** MALP-2 and **PGN**."	
		DC	"Compared with colonic DCs from healthy controls, more **DCs from patients** with **active Crohn's disease produced** IL-12 and **IL-6 (Figure 8).** In contrast, production of IL-12 and IL-6 by DCs from patients with ulcerative colitis did not differ from that of control DCs."<br>**Figure 8 B**	[38]
		DC AND (LPS OR PGN)	"Monocytes and **circulating conventional DCs (cDCs)** activated **by lipopolysaccharide (LPS) and peptidoglycan,** which produce **large amounts of** IL-1b and **IL-6.**"	[39]
		(Th17 OR MACR) AND IL23	"..**IL-23 binds to Th17 T cells and macrophages, promoting the release of IL-17, IL-6, IL-1 and TNF**."	[40]
			"**IL23** is produced by activated myeloid cells including macrophages and dendritic cells (DCs) following bacterial stimulation20 and drives or via CD40 signalling,6 increases in a number of inflammatory cytokines in the intestine in the absence of T cells, including TNFa, IFNc, **IL6** and IL17."<br><br>"The IL23R is expressed by activated DCs and macrophages, and IL23 can induce production of inflammatory cytokines by macrophages.9"<br><br>Ref 9. [41] Does not indicate that IL23 is implicated in IL6 synthesis.<br>"We analysed the DLNs from MOG-immunized mice by real-time quantitative PCR to determine in vivo expression of proinflammatory cytokines. p19 2/2 mice produced a strong in vivo IFN-g, IL-1b, tumour-necrosis factor (TNF), IL-6 and granulocyte/macrophage colony-stimulating factor (GM–CSF) response, comparable to wild-type controls, whereas p402/2 mice showed no induction of proinflammatory cytokines, including IL-23 p19 (Fig. 3c). DLNs from p35 2/2 mice expressed elevated levels of IL-1b, TNF, IL-6, GM–CSF and IL-23 p19, but not IFN-g"	[42]
			"**These observations suggest that IL-23 can induce chronic inflammation through 2 independent pathways:** (a) **activation of Th17/ThIL-17 cells**; and (b) i**nduction of IL-1 and IL-6 production via myeloid cell activation (**Figure 2)."<br>In chronic inflammation: **IL-23** also acts on dendritic cells and macrophages in an autocrine/paracrine manner to stimulate the generation of proinflammatory cytokines, such as IL-1, IL-6, and TNF-α.<br><br>There is not reference for the statement (IL6 = MACR AND IL23)	[43]

		IL22	"In addition to regulating the expression of antimicrobial peptides and defensins, **IL-22 contributes to the expression** of a range of genes encoding molecules involved in inflammatory responses, including **IL-6,** G-CSF, IL-1α, LPS-binding protein, serum amyloid A, α1-antichymotrypsin and haptoglobin 15,20–23"	[44]
			15, 20, 22, 23: Do not provide relevant information<br>21: MICE, pneumonia: "In the absence of IL-17A, **antibody neutralization of IL-22** resulted in **a further reduction in IL-6** and CCL3 abundance, suggesting a requirement for both IL-17A and IL-22 in this response."	[45]
		FIBROBLAST	"**IL-6 is produced by cells of the innate immune system such as DCs**, monocytes, **macrophages**, mast cells, B cells, and subsets of activated T cells, but also by tumor cells, **fibroblasts**, endothelial cells, and keratinocytes (37, 38).	[15]
			38: Nothing related to fibroblasts.<br>37: **Human IL-6 purified from supernatants of** T cells (7), **fibroblasts** (19), and peripheral blood mononuclear cells (27)<br>19: Reference from 1988	[46]
**IL6** = (MACR **AND** PGN) **OR**(DC **AND** (LPS **OR** PGN)) **OR** (Th17 **AND** IL23) **OR** (NFkB **AND NOT** (IL4 **OR** IL10)				
9	TNFa<br><br>Upregulated in CD and UC<br>[30]	LPS AND NFκB	"**TNFa induces its own transcription through (NFkB) activation** " 7	[47]
			7: Mouse macrophages, and not very clear	[48]
			"Nuclear factor kappa- B **(NFkB) is activated by** phosphorylation of inhibitory kappa-B (I?B) and then translocates to the nucleus to **activate TNF promoters**. " **10**	[49]
			10: "NFkB is activated by inhibitory kappa B (I?B) phosphorylation (which disengages its inhibitory subunit, I?B) and translocates to the nucleus to activate TNF promoters"<br><br>**Figure 4**<br><br>"LPS-LBP-CD14 interaction provokes rapid activation of protein tyrosine kinase (PTK) causing tyrosine phosphorylation of several intracellular protein kinases **(116, 244, 252, 296–298)**. PTK activates a pathway involving Ras/Raf-1/mitogen-activated protein kinase kinase (MEK)/MAPKs/NF?B (46, 70, 162, 163). Several studies have	[50]

			demonstrated that LPS activates PTK and that PTK inhibition abolishes downstream activation of MAPKs, TNF and IL-1 production, and macrophage-mediated tumoricidal activity (**46, 127, 205, 237, 282**)."	
			**116→** "Endotoxin induces rapid protein tyrosine phosphorylation in 70Z/3 cells expressing CD14.<br>LPS (endotoxin) → CD14"<br>**224 →** Murine Macrophages<br>**252** → Nothing relevant unless this reference: **251**[51]→ mouse<br>**296–298 →** mitogen-activated protein kinases in macrophages….<br>**46, 127, 205 →** Nothing relevant.<br>**237** → "The early steps in LPS signal transduction involve the tyrosine phosphorylation and activation of a number of kinases of the src family, and inhibition of this pathway causes a severe impairment in the production of the cytokines TNF-a and IL-1 p. We find that LPS-induced macrophage activation also involves the Raf-1 kinase, a key component in mitogenic signal transduction. Treatment of BAC-1.2F5 macrophages with LPS causes phosphorylation and activation of Raf-1" **But: mouse macrophage cell line, BAC 1.2F5.**	**116**[52]<br>**237** [53]
			"The canonical pathway is induced by tumour necrosis factor-alpha (TNF-alpha), interleukin-1 (IL-1) or byproducts of bacterial and viral infections. This pathway relies on IKK- mediated IkappaB-alpha phosphorylation on Ser32 and 36, leading to its degradation, which allows the p50/p65 NF-kappa B dimer to enter the nucleus and activate gene transcription." **Figure Kegg**	http://www.kegg.jp/kegg-bin/highlight_pathway?scale=1.0&map=map04064&keyword=tnfa
			Figure Kegg	http://www.kegg.jp/kegg-bin/highlight_pathway?scale=1.0&map=map05321&keyword=tnfa
			**Figure 1**	[54]
		IL-1b	"**IL-1β** on the other hand, can **activate the release** of other proinflammatory cytokines such as **TNF-α**, IL-23 and IL-6, which can promote a Th17 bias adaptive response, or IL-1β can co-stimulate IL-6 production and act as a growth factor for B cell proliferation and enhanced antibody production in the Th2 response" [30,71,116]	[16]
			**30:** Do not mention that IL1b induces TNFa	[55]

			71. Do not mention that IL1b induces TNFa	[56]
			116: Do not mention that IL1b induces TNFa	[57]
		(IL2 OR IL15) AND NK	**“IL-2 and IL-15** equally **lead to** the upregulation of perforin and granzyme B expression as well as **cytokine production** including **IFN-g, TNF-a,** and granulocyte-macrophage colony- stimulating factor **(GMCSF)** by **NK** cells.”	[58]
		AND NOT (IL10 AND (TLR2 OR TLR4))	“To confirm the role of **endogenous IL-10** in **suppressing TLR2-induced TNFa** production, we used anti-IL-10 neutralizing antibodies”<br>“IL-10 and Stat3 mediate feedback inhibition of TLR2-induced TNFa production”<br>**“Compared with TLR2, TLR4** induced **higher levels of TNFa**, **with an incremental increase in TNFa production when IL-10 was neutralized**”<br>**Figure1 E**<br><br>“We “**IFN-g augments induction of TNFa by** TLR ligands, immune complexes, and zymosan by **suppressing IL-10 production**”<br>**Figure 2**	[59]
			“Interestingly, at 48 h, a 13-fold increase in IFN-γ and a 2-fold increase in TNF-α mRNA levels were observed in IL-10– depleted cultures compared with control cultures (mouse)”	[60]
		TGFb	“IL-1, IFN-c, **GMCSF**, **TGF-b**, and **TNF-a** itself by autocrine mechanisms are able to **induce the expression of TNF-a**.”19	[61]
			19: Do not mention that TGFb induces TNFa secretion.	[49]
		CD4_NKG2D	“We show that during **the NKG2D-TCR costimulation** of lymphocytes, all **IL-17–secreting CD4+ T** cells also **express TNF-a”**<br>**Figure 4**	[62]
		CD8_NKG2D	“Our results show that cells that were primed with anti-**CD3 mAb** in the presence of **NKG2D** costimulation tend to produce more t**ype 1 cytokines (IFNG and TNFa)**”	[63]
			**“CD8+NKG2D+ T** cells almost did not produce IL-17 but **highly expressed IFN-g**”<br>**Figure 6**	[62]
		NK_NKG2D	“In human **NK cells**, crosslinking with multivalent soluble ligands **(ULBPs)** stimulates the **production of several cytokines including IFN-γ, TNFα**” 8,22,49	[64]

		FIBROBLAST AND IFNg	"CD4+CD28- T cells are thought to promote the formation and maintenance of RA inflammatory lesions mainly through IFNg release. **IFNG** perpetuates synoviocyte pathology, which is associated with **secretion of TNFa**, IL-15, and tissue-injurious metalloproteinases **by** synovial **fibroblasts** and **macrophages"**	[65]
		MACR AND (IFNg AND LPS)	"Macrophages are the main source of TNF..."	[49]
			"Wild-type macrophages showed dose-dependent production of IL-6, TNF-a and IL-12 in response to MALP-2 and PGN": **MOUSE**	[37]
			"**Preincubation of macrophage**s with interferon **(IFN)-g augments TNF** production **in response to LPS** stimulation" **7**	[47]
			"We report that IFN-g augments induction of TNFa **by TLR ligands**" "Control or **IFN-g-activated human macrophages** were stimulated with 10 ng/ml of Pam3Cys (anti IL10) for indicated periods of time, and levels of IL-10 and **TNFa** in culture supernatants were determined by ELISA."	[59]
		MACR AND PGN	"**Wild-type macrophages** showed **dose-dependent production of IL-6, TNFa** and IL-12 **in response to** MALP-2 and **PGN**."<br><br>"Cytokine production in peritoneal macrophages in response to TLR ligands. a, Peritoneal macrophages from wild-type (þ/þ) and TIRAP-deficient (2/2) **mice** were cultured with 1 ngml21 LPS, 10nM R-848 or 1 g m M CpG DNA in the presence (IL-12) or absence (IL-6 and TNF-)of 30 ngml21 IFN- a for 24 h. Concentrations of IL-6, TNF-a and IL-12 in the culture supernatants were measured by enzyme-linked immunoabsorbent assay (ELISA)."<br>**Figure a and b**	[37]
		MACR AND IL2	"**IL-2,** granulocyte-macrophage colony–stimulating factor, and macrophage colony–stimulating factor have also been reported to induce TNF **release from macrophages.**"9	[47]
			9 "Other cytokines, IL2, ..."	[66]
			14 "As a result of our in vitro and in vivo studies we propose that some of the in vivo effects of IL-2 may in fact be mediated by TNF produced by macrophages, including some of. the toxicity and/or anti-tumour activity associated with IL-2- based clinical immunotherapy regimens."<br>**Figure 1**	[67]
		NK AND IL23	**Figure 5. IL-23 enhances NK cell proinflammatory cytokine secretion and cytolytic activities**. (A and B). **Purified NK cells** (2.0?105/ml) from PB of ? g/ml) **IBD patients and healthy controls were culture**d in a 96-well flat-bottom plate, **immobilized with**	[68]

			**human IgG (hIgG**; 100 ? g/ml) **in the absence or IFN-G and TNF** by ELISA. Results are representative of three independent experiments. Figure 5	
			"Secretion **(induced by IL23) in IBD"**	[69]
		(MACR OR DC) AND IL23	"**TNF-α is produced in response to bacteria** or **IL-12 and IL-23 cytokines by mononuclear cells** through **the activation of cellular receptors** (e.g. the Toll-like receptor 4), which are critical in CD4 lymphocyte differentiation**(5,6)**"	[40]
			**5**"it induces the **NK cells to secrete IFN-γ and TNF-α**[75,76]"<br><br>→ It did not mention that TNFa is produced by **mononuclear cells and IL12 or 23.**	[70]
			**6** It did not mention that TNFa is produced by **mononuclear cells and IL12 or 23.**	[71]
			table 1	[72]
			"..**IL-23 binds to Th17 T cells and macrophages, promoting the release of IL-17, IL-6, IL-1 and TNF**. b) IL-23 is one of the cytokines of IL-12 family and, like IL-12, is a pro-inflammatory cytokine. Il-23 shares p40 subunit with IL-12, and holds a specific subunit, p19. IL-23 binds to a heterodimeric complex receptor consisting of IL-12 receptor (IL-12 R) β1, binding p40 subunit, and IL-23 receptor complex (IL-23 R), binding p19 subunit, while IL-12, composed of p40 and p35 subunit, binds to a different complex, composed of IL-12 R β1 and IL-12 R β2.	[40]
			"In innate colitis, **IL-23 was found to control the production of a number of inflammatory cytokines such as IL-1b, IL-6, TNF-a and IFN-g** and both TNF-a and IFN-g were found to play a functional role (7). This finding is consistent **with the ability of IL-23 to induce production of these inflammatory cytokines by activated DCs and macro-phage**s (34)."	[73]
			7 → T and B cell-deficient mice	[74]
			34 → "IL-23 induced the production of IL-1b and TNF by peritoneal macrophages, which occurred even in the presence of neutralizing antibodies to IFN-g (13)."	[75]
			13 → MICE	[41]
			75 It did not mention that TNFa is produced by **NK.**	[76]

		NK AND (IL12 AND (IL15 OR IL2 OR IL18))	76 “Whereas **IL-2 and IL-12 synergize** to induce IFN-y production**, the effect of the two cytokines on TNF production by NK cells is additive**. To analyze a possible role of these cytokines in the IL-12-dependent inhibition of the IL-2-induced proliferation in NK and TCR-yG+ cells, proliferation was studied. As shown in Figure 8, **anti-TNF,** but not anti-IFN-y antibodies, **were able to reverse almost completely the inhibitory effect of IL- 12** on **IL-2-induced proliferation of NK cells”**	[77]
			**Figure 4. IL15 and IL-2 stimuhte CD56 dim NK cell cytokine production in a similar fashion.** 10^5 sorted CD56 dlm NK cells (>97% pure) were plated in U-bottom wells in 200 #1 of medium supplemented with one or more of the following cytokines: TNF-~, (300 U/ml), IL-12 (10 U/ml), I1.,2 (10 ng/ml), or IL-15 (10 ng/ml). Cells were incubated for 72 h at which time the culture supernatants were harvested and assayed for the presence of either IFN-3, (A), TNF-~ (B), or GM-CSF (C), using an ELISA. Cytokine production is measured in pg per ml”	[78]
			**Figure 3:**	[79]
			“We show that although **IL12-cultured NK cells produced** abundant IFNg, **TNFa,** and GM-CSF in response to stimuli acting on the NKp46-activating receptor, IL-4-cultured NK cells did not release detectable levels of these cytokines.”<br><br>**Figure 2:**<br>“**NK cells from donor A were cultured overnight in the presence of the indicated cytokines**, used alone or in combination. Subsequently, NK cells were stimulated with either plate-bound anti-NKp46 (b) or PMA plus ionomycin (c). After 4-h stimulation, supernatants were harvested and as-sessed for IFN-g, TNF-a, and GM-CSF (b) or for IFN- , TNF- , GM- IL-5, and IL-13 (c) by specific ELISA (n ? 3; mean ? SD). Data are representative of five different experiments.”	[80]
**TNFa** = ((NFkB **AND** LPS) **OR** ((MACR **AND** (IL2 **OR** (IFNg **AND** LPS) **OR** PGN)) **OR** ((NK **AND** (MDP **OR** PGN **OR** LPS) **AND** (IL23 **OR** IL12 **AND** (IL2 **OR** IL15)))) **OR** (FIBROBLAST **AND** IFNg) **OR** (CD4_NKG2D **OR** CD8_NKG2D **OR** NK_NKG2D) **AND** (IEC_MICA_B **OR** IEC_ULPB1_6))) **AND NOT** (IL10 **AND** (TLR2 **OR** TLR4) **AND** TNFa)				
10	TGFb<br><br>Upregulate d in CD and UC [81]	Treg	“Active **TGF-β is a cytokine produced by** various cell types, including **natural Treg cells (nTreg)** and cells of the innate immune system”	[15]
			**TGF-β1** induces FOXP3 → Treg transcription factor<br>**Figure 2: a and b**	[82]
			**“Treg cells** exert their immunoregulatory functions through various mechanisms requiring CTLA-4, direct killing of antigen presenting cells or T cells, consumption of IL-2, **and the production** of immunosuppressive cytokines such as IL-10, **transforming growth factor (TGF)-b**, IL-35 and galectin-1 [53”]”	[83]

			"**Treg** cells suppress adaptive immune responses through cell–cell contact dependent mechanisms or **secretion** of the anti-inflammatory cytokines IL-10 or **TGF-β**"(14, 15)"	[84]
			"TLR2 also activates **regulatory T cells** via PI3K **to secrete transforming growth factor-b 27**"<br>PI3K is part of MAPK	[14]
			**27:** "The finding that **HSP60-treated Tregs** significantly **upregulated their secretion of TGF-β and IL-10** (Figure 7A) suggests that costimulatory signals, such as HSP60, might account for the importance of these cytokines detected in Treg-mediated suppression in vivo (13–15)."	[85]
			"There are three isoforms of TGF-, TGF-1, −2, and −3. TGF-1 is the predominant isoform, present at sites of inflammation/injury, and is the major isoform secreted by circulating monocytes and tissue macrophages (Assoian et al., 1987; Grotendorst et al., 1989)."	[86]
			Fig 1: "In the present of inflammation and dysregulated mucosal homeostasis, newly recruited monocyte-derived macrophages (and other innate host defense cells) generate transforming growth factor-β (TGF-β) and chemokines to recruit monocytes and other leukocyte populations to contain the infection."	[87]
		MACR	"The studies reported here demonstrate that TGF-b, rmRNA is expressed at similar levels in unstimulated monocytes and in monocytes activated to become macrophages".<br><br>"**TGF-b** itself i**s only secreted by activated macrophages."**	[88]
			"Upregulation of stem cell markers and mitotic activity in the cultures was shown to be controlled by autocrine production/secretion of activin A and transforming growth factor-beta (TGF-b). These reprogrammed monocyte derivatives were termed "**programmable cells of monocytic origin" (PCMO**)."<br><br>"Moreover, we found that TGF-b is secreted by PCMO into the culture supernatant as determined by ELISA, with levels of TGF-b1 declining during monocyte conversion to PCMO."	[89]
		IL6<br>IL21	**INHIBIT SECRETION**<br>**IL-6 and IL-21** inhibit FOXP3→ **No TGF-B por parte deTreg**	[82]
		While the ELISA analyses, which measured total (latent and active) **TGF-b, showed increased levels of TGF-b1 and TGF-b2in IBD patient plasma relative to uHC (unrelated healthy controls),** the differences did not reach statistical significance.		[81]
**TGFb** = Treg **OR** MACR				

<table>
<tr><td rowspan="7">11</td><td rowspan="7">INFLAM</td><td colspan="2"><u>Inflammasomes</u> **are formed by**
● an **adaptor protein**
● an i**nflammatory caspase.**
● a **sensor protein**. Which are:
○ **PRRs** that act as scaffolds. Could be a member of the NLR:
■ **NLR family** include:
● the **NOD** (NOD1, **NOD2**, NOD3/NLRC3, NOD4/NLRC5, NOD5/NLRX1, CIITA), **NLRP** or **NALP** (NLRP 1–14)
● **and IPAF** (NLRC4 and NAIP) subfamilies.
○ or a pyrin (PYD) and HIN domain-containing protein **(PYHIN) family.**</td><td>[21]</td></tr>
<tr><td>MDP</td><td>“So far, **bacterial muramyl dipeptide (MDP**) and lethal toxin (LeTx) produced by Bacillus anthracis have been shown to **activate the NALP1 inflammasome** [35,54,82].”

“Early studies by Faustin et al [38] using purified recombinant proteins defined the minimal requirements for caspase-1 activation in the NLRP1 **inflammasome** as being; **MDP**, NLRP1, ATP and procaspase-1, a process not requiring ASC, but enhanced in the presence of ASC.”</td><td>[16]</td></tr>
<tr><td>LPS</td><td>“The **inflammasomes** can sense an array of stimuli via PRRs, including PAMPs from the bacterial cell wall (e.g. lipopolysaccharides **(LPS),** lipoproteins or flagellin)....[5,11,17].”</td><td>[21]</td></tr>
<tr><td rowspan="2">PGN</td><td>“The **NLRP3 inflammasome** uses the adaptor molecule ASC and caspase-1 to convert pro-IL-1b and pro-IL-18 into their active forms.”
“**NLPR3 can be activated by** an array of stimuli including microorganisms such as viruses,fungi and bacteria; individual bacterial components such as **LPS**, single stranded RNA (ssRNA), double- stranded RNA (dsRNA), peptidoglycans (**PGN**), CpG DNA, MDP and bacterial pore-forming toxins [74,78,91].”</td><td>[21]</td></tr>
<tr><td>“NLRP3 inflammasome is involved in the induction of ROS on IL-1b production by **PGN/LPS**-induced MDMs”</td><td>[90]</td></tr>
<tr><td rowspan="2">Th0_M</td><td>“the level of **negative regulators of inflammasomes**, including activated T cells and microRNAs, have the potential to augment the inflammatory response [21,22]”
“Human **CD4+ memory T** cells **dampen NLRP3 activation** via downregulation of P2X7R signaling [88].”</td><td>[91]</td></tr>
<tr><td>[88] “**human activated CD4+CD45RO+ memory T-cells** specifically s**uppress P2X7R-mediated NLRP3 inflammasome activation,** without affecting P2X7R-independent NLRP3 or NLRP1 inflammasome activation.”

“**Soluble Caspase-1 Release is Decreased in Monocytes** Cultured **with Activated CD4+CD45RO+ Memory T-cells**”</td><td>[92]</td></tr>
</table>

			Not included in the boolean function because Th0_M inhibits only two pathways of inflammasome activation. It could be expressed as a partial inhibition.<br><br>Adding: **AND NOT** (Th0_M **AND** (PGN **OR** MDP **OR** LPS) would lead to the total inhibition of the INFLAM node in a CD patient simulation (see Th0_M Boolean function) and will not be real.	
**INFLAM** = MDP OR LPS OR PGN				
12	Th0<br><br>Downregulated<br>CD		"Naive T cells are inexperienced T cells that have yet to see their cognate antigen. **T-cell receptor (TCR) engagement in an inflammatory cytokine milieu induces the cell to undergo numerous changes**, including chromatin remodeling and DNA methylation modifications, leading to the induction or repression of transcription factors in differentiated cell subsets [1]."	[93]
		LPS<br><br>MDP<br><br>PGN	**"Lymphocytes circulate in the blood** and the **lymph** and are also found in large numbers in **lymphoid tissues or lymphoid organs**, which are organized aggregates of lymphocytes in a framework of non lymphoid cells."<br><br>"Once they have completed maturation, **both types of lymphocytes enter the bloodstream as mature naive lymphocytes**. They **circulate through the peripheral lymphoid tissues**, in which an adaptive immune response is initiated if a lymphocyte meets its corresponding antigen."	IBJ I (INTRODUCTION TO IMMUNOBIOLOGY AND Basic Concepts in Immunology)
			"Absolute quantitation of T cell subsets revealed that **the higher proportion of memory T cells was attributable to lower concentrations of CD45RA naïve T cells in patients and siblings compared with controls**, whereas memory T cell frequencies did not differ significantly compared with ontrols, figure 1B–E."<br>"**Although concentrations of T cells in patients were contributed to by the use of immunosuppressants by half of patients, concentrations of naïve T cells were also reduced in immunosuppressant naïve siblings, and in the CD4 naïve T cell population** this difference was significant. Whole blood was labelled with fluorescently conjugated monoclonal antibodies before acquisition of data using a LSRII flow cytomete"<br><br>**Figure 1 D**	[94]
			**<u>Comment:</u>** Th0 node represent the CD4+T activated (Th0 naive once their TCR have been encountered Ags)<br><br>Th0 is activated by antigens instead of APC (MACR OR DC) because MACR and DC nodes are created as immune cells (cytokine producers, with its inhibitors) Antigen presenting takes part always there are antigen.	
**Th0** = U (LPS OR MDP OR PGN OR GLY)				

13	Th0_M<br><br>Upregulate d in CD [94]	**Figure 3. Th0_M time (days):**		[95]
		IL23 OR IL12	“IL-23 stimulates the proliferation of CD4+ memory T cells rather than naive CD4+ T cells **14**”	[96]
			**14** :“IL-23 induces strong proliferation of **mouse memory** (CD4+CD45Rblow) T cells, a unique activity of IL-23 as IL-12 has no effect on this cell population”<br><br>“Similar to IL-12, **human IL-23 stimulates IFN-g production and proliferation** in PHA blast T cells, as well as **in CD45RO (memory) T cells**”<br><br>**CD4+ memory Tcells: p19p40 complex: expressed by human DC. Interaction with IL-12Rb1 and an additional receptor subunit.**<br>“In contrast, both mouse and **human memory T cells respond strongly to p19p40 by enhanced proliferatio**n and, in the case of human cells, by enhanced IFN-g production. **Interestingly, human memory T cells but not mouse memory T cells were also very responsive to IL-12**.”<br><br>**Figure c and d:**<br><br>**“(C and D) p19p40 has a more pronounced effect on human CD45RO (memory) than on CD45RA (naive) T cells. FACS-purified CD45RA and CD45RO T cells were cultured on anti-CD3** (10 ?g/ml) **and anti-CD28** (1 ?g/ml) coated 96-well plates and stimulated with 40 ng/ml **hHy-p19-p40**, **1 ng/ml IL-12** (R&D Systems), **or 100 U/ml IL-2** (R&D Systems) for 60 or 136 hr. IFN-g production (C) and proliferation (D) were determined as in (A) and (B).”	[97]
		IL15	“L-15 is known to exert essential signals that favor **cell proliferation, development, and maintenance** of memory T cells as well as promote survival and increases cytotoxicity of T and NK cells”	[58]
		IL2<br><br>IL4	“Despite the efficiency of AICD (activation induction cell death), the expression of several molecules may allow cells to escape apoptosis. Some of these factors are secreted growth factors such interleukin-2 **(IL-2), IL-4, IL-7 and IL-15**, which have been shown to be **antiapoptotic** in vitro as well as in vivo [7, 8]. Others are antiapoptotic members of the TNF family, such as CD30 [9] and 4-1BB [10, 11]. In addition, several Bcl-2 family members, such as Bcl-x, have been shown to inhibit apoptosis triggered by a variety of stimuli [12]. The action of these antiapoptotic proteins **may allow** a cell to escape AICD and **form long-living memory cells”**	[98]
		“**The proportion of blood T cells that were CD45RA− memory T cells was significantly higher in patients and siblings** compared with controls, figure 1A.”<br>**Figure 1A**		[94]

		(Th0_M **AND** (IL15 **OR** IL2 **OR** IL4)) not included in the boolean function. Once Th0_M is activated, it will remain activated. Upregulation would not affect.		
		**Th0_M** = ((Th0 **AND** (IL23 **OR** IL12)) **OR** Th0_M		
14	IL18<br><br>Upregulated in CD and UC<br>[30]	NFkB	"Stimulation of NLRs results in activation of the caspase-1 signaling complex (**known as the inflammasome**) mediating cleavage of pro-IL-18 and pro-IL-1b or activation of NF-kB signaling."	[24,99]
			"The processing and secretion of active IL-1b and IL-18 by innate immune cells is dependent, first, on induction of pro–IL-1b and **pro–IL-18 through TLR-induced NF-kB** activation and then on activation of caspase- 1, which is required for cleavage of pro–IL-1b and IL-18 into mature IL-1b and IL-18."<br><br>**TLR → NFkB → proIL18 → caspase-1 → IL18**<br><br>**Comment:** INFLAM is not a node. NFkB is activated by all antigens and LPS is required as well	[100]
		((MACR OR DC) AND LPS)	"**Upon stimulation with LPS, macrophages secrete biologically active IL-18** in **a caspase-1-dependent manner."**<br>**Figure1:**<br><br>"Dendritic cells express IL-18 mRNA and produce mature IL-18" **[45].**	[101]
			**45:** "Signals for IL-18 mRNA are detectable in various DC fractions by Northern blot analysis."<br><br>"We, therefore, **highly enriched various subtypes of DC, such as lymph node-derived DC**, bm-DC **and human blood-derived DC**, and assayed for the presence of IL-18 mRNA by Northern blot **and tested the supernatants for release of functional IL-18 protein**. **All DC were shown to be sources of IL-18 mRNA and protein.**"<br><br>**Enriched:** "<br>Briefly, **whole blood buffy coats were used for preparation of PBMC** isolated in Ficoll-Histopaque (Seromed, Berlin, Germany). Lymphocytes were depleted using immunomagnetic beads coated with either anti-CD2- or anti-CD19 mAb (Dynal) and **the resulting cell fraction was cultured** in X-VIVO-15 (Bio-whittacker, Walkersville, MD) supplemented with 800U/ml hGM-CSF (Leukomax; Sandoz, Basel, Switzerland), 1000U/ ml hIL-4 (PBH) and 1% autologous plasma. Cells were fed every other day with fresh complete medium containing 1600U/ml hGM-CSF and 1000U/ml hIL-4.On day 7, nonad-herent cells were transferred to fresh six-well plates in complete culture medium and stimulated with 10ng/ml hIL-1 g (PBH), 10 ng/ml hTNF- § (PBH) and 1000U/ml hIL-6 (R&D Systems) **to induce and stabilize the maturation of DC.**"<br>**Figure2:**<br><br>"**Human CD83+ blood- derived DC** express IL-18 mRNA. Blood-	[102]

			derived DC were prepared and enriched for CD83+ cells as described, and total mRNA was analyzed by quantitative RT-PCR.”	
		MACR OR DC	“However, in agreement with previous reports (20, 31), these proinflammatory cytokines as well as endotoxin induced expression and release of IL-18 by MØ”<br><br>“Proinflammatory cytokines including IL-1b , TNF- a , or IL-6, as well as LPS, induce IL-18 gene expression **(20)**”	[103]
			**20:** “Murine and human dendritic cells constitutively express IL-18 mRNA and produce mature IL-18 (68)”	[104]
		**Comment**: NFkB could be deleted from the Boolean function because it is going to be activated if LPS is activated. But it is possible that there is a polymorphism that alters NFkB and leaving the node in the function could be implemented.		
**IL18** = ((MACR **OR** DC) **AND** LPS) **AND** NFkB				
15	IL1b<br><br>Upregulated in CD and UC [30]	NFkB	“Stimulation of NLRs results in activation of the caspase-1 signaling complex (**known as the inflammasome**) mediating cleavage of pro-IL-18 and pro-IL-1b or activation of NF-kB signaling.”	[24,99]
			“The processing and secretion of active IL-1b and IL-18 by innate immune cells is dependent, first, on induction of pro–IL-1b and **pro–IL-18 through TLR-induced NF-kB** activation and then on activation of caspase- 1, which is required for cleavage of pro–IL-1b and IL-18 into mature IL-1b and IL-18.”<br><br>**TLR → NFkB → proIL1b → caspase-1 → IL1b**<br><br>**Comment:** INFLAM is not a node. NFkB is activated by all antigens and LPS is required as well	[100]
		(MACR OR DC) AND LPS	“The **inflammasome functions** to convert inactive procaspase-1 to active caspase-1, which cleaves the inactive **IL-1β** precursor to a secreted, **active cytokine**. Caspase-1 also cleaves the precursors of IL-18, IL-33, and IL-1F7.”<br>“The primary sources of **IL-1β** are the blood monocyte, **tissue macrophages, and dendritic cells**.”	[105]
			**“Inflammasome** formation is essential for the production of the **mature IL-1β and IL-18** cytokines and is seen as a two-step process [1]. Step one, is the p**riming step and involves TLR signaling to induce NF-kB transcription of proIL1β, proIL18 and inflammasome component** such as; NLRP3 and NLRP1 [3,82]. Step two; another stimulus leads to inflammasome oligomerization, caspase1 auto activation, cytokine cleavage and mature IL-1β and Il-18 production and cellular release.”<br><br>**Comment:** INFLAM is not a node. NFkB is activated by all antigens	[16]

			and LPS is required as well	
			“The peptides containing C-terminal 15 amino acids of EscI protein could significantly activate NLRC4 inflammasome responses in macrophages pre-stimulated with lipopolysaccharide. Intracellular caspase-1 was activated and pyroptotic dead cells were found after peptides delivery. The contents of cytokines, IL-1β and IL-18, in supernatants were elevated significantly compared with that of the control ($P < 0.05$). Besides, through comparison of IL-1β contents under different stimulation conditions, 4 h incubation after peptides delivery (peptides: lipofectamine 2000 = 70 μg/μL) could obviously promote the secretion of IL-1β.”<br>No paper access.<br>“The peptides containing C-terminal 15 amino acids of EscI protein could significantly activate NLRC4 inflammasome responses in macrophages pre-stimulated with lipopolysaccharide. Intracellular caspase-1 was activated and pyroptotic dead cells were found after peptides delivery. The contents of cytokines, IL-1β and IL-18, in supernatants were elevated significantly compared with that of the control ($P < 0.05$). Besides, through comparison of IL-1β contents under different stimulation conditions, 4 h incubation after peptides delivery (peptides: lipofectamine 2000 = 70 μg/μL) could obviously promote the secretion of IL-1β.”<br>”Peptides containing C-terminal 15 amino acids of E. coli EscI protein can significantly induce NLRC4 inflammasome activation in macrophages.”<br>**No paper access.**<br>Figure 3. Contents of IL-1β(A) and IL-18(B) in supernatants.	[106]
		AND NOT (IL1b AND IL10)	“IL10 was shown to inhibit macrophage activation and production of cytokines such as TNFa and IL-1 (19-24).”	[35,107]
			**Activation of monocytes with LPS** (1/~g/ml) resulted in production of high levels of IL1a, IL1b IL6, IL8, IL10, TNFa, GM-CSF, and G-CSF. **Interestingly, IL-10 inhibited the production** of IL1a, **IL1b**, IL6, IL8, IL10, TNFa, GM-CSF, and G-CSF **to various extents. The inhibition of IL1b and IL6 production was less pronounced**.	[35,107]
		MACR AND IL23	“..**IL-23 binds to Th17 T cells and macrophages, promoting the release of IL-17, IL-6, IL-1 and TNF**.	[40]
			Figure:	[108]
			“IL-23 induced the production of IL-1b and TNF by peritoneal macrophages, which occurred even in the presence of neutralizing antibodies to IFN-g (13).”	[75]
			13 → MICE	[41]

IL1b = ((MACR OR DC) AND LPS AND NFkB) AND NOT (IL1b AND IL10)				
16	IFNg<br><br>Upregulated in UC and CD<br><br>[30,109]	NK AND IL23	**“IL-23** potently **induces IBD NK cell differentiation**, leading to secretion of proinflammatory cytokines such as TNF and IFNg”	[69]
			**“Human IL-23R is expressed by both T cells and NK** cells, including NKL cells (Fig. 5, a and b), **consistent with the ability of these cells to respond to IL-23.** We demonstrate here that **IL-23 enhanced the production of IFN-? by NKL** cells. The combination of IL-2, IL-18, and IL-23 induced levels of IFN-g that were substantially greater than those induced by either cytokine alone or when combined solely with IL-2”	[110]
		AND NOT (TGFb AND IFNg)	Inhibits production DOWNREGULATION (concentration dependent). “The mean production of IFNg was respectively 45.9%, 41.6%, 69.9% and 92.1% of that measured in the presence of medium only”<br><br>“**Crohn’s disease** as they are in cells from controls.40 **We confirmed this here in that TGFb did not shut off IFNg production, only reducing it by about 50%**.” → downregulation	[109]
		Th1	Reviews: IFNg secretion by Th1 cells.<br><br>• **Silva et al. 2005:** “Using organ cultures of colonic tissue explants from pediatric CD patients, we observed that recombinant human IL-15 (rIL-15) inhibited the production of TH1 cytokines INF-g and TNF-a”<br><br>• **Strober and Fus 2011**: Mice	[109,111–113]
		(MACR OR Th0) AND IL18 AND IL12	“**MØ expressed IFNg more uniformly after stimulation with IL-12/IL-18**” (both).	[103]
			“**Human T** cells also r**equire stimulation with both IL-12 and -18 to produce significant amounts of IFNG [82 ,91**”	[101]
			**82:** “IL-12 and IL-18 Differentially Regulate the Transcriptional Activity of the Human IFN-g Promoter in Primary CD4+ T Lymphocytes”<br><br>“The data reported here show that that **IL-12 and IL-18 act differentially to activate IFN-g gene transcription in primary human CD4+ T cells**. Furthermore, they suggest that both AP-1 and STAT4 are required for IL-12-dependent IFN-g promoter activation, whereas IL-18 causes direct promoter activation via AP-1. This differential activation of the IFN-g promoter gives further insights into molecular pathways governing Th1 T cell development and differentiation.”	[114]
			**91**: Human CD4+CD45RA+ T cells<br>“The requirement of pretreatment with IL-12 for rendering T cells	[115]

			responsive to IL-18 suggests that IL-12 stimulates T cells to express IL-18R.”	
		AND NOT (IFNg AND IL10)	“**Interestingly, at 48 h, a 13-fold increase in IFN-γ and a 2-fold increase in TNF-α** mRNA levels were observed i**n IL-10– depleted cultures compared with control cultures** (Figure 3A).”	[60]
		Th17 AND (PGN OR MDP OR LPS)	“Secretion from Th0 stimulated with cytokines which induces Th17 cell differentiation.”	[96]
			“A prominent feature in these cells is a functional plasticity towards the Th1 subset, being reported that **some Th17 cells are able to produce both IL-17 and IFN-*γ*** **[15]**.”<br>“Based on this, it is obvious that Th17 cells play an important role in IBD pathogenesis. It has been shown that the numbers of IFN-*γ* secreting T cells in peripheral blood from IBD patients significantly correlated with disease severity in CD but not in UC **[47].**”<br>---<br>**[47]** → do not mention IFNg	[116]
			**[15]**: “In this study, we demonstrate the existence of remarkable proportions of **Th17 and of IFNg producing Th17** (Th17/Th1) in the **gut of subjects with Crohn's disease (CD)**.”<br>“Proportions of Th17 also producing IFNg were higher among gut T cells than PB CD4 T cells from subjects with CD.”	[117]
		AND NOT Th2	“**Th2 inhibits IFN-g secretion** (GATA-3 represses T-bet expression and IFN-g secretion and therefore TH1 differentiation)”	[118]
		Treg	Treg-Th17 like: “Moreover, **CD-derived LP FoxP3+IL-17+ Treg cells also acquired the capacity to produce significant amounts of another effector cytokine, IFN-γ**” Fig 4	[119]
		NK_NKG2D	“Next we determined whether binding of ULBP to NK cells induced any biological effects. Purified NK cells were stimulated with IL-15 for 20 h, washed and recultured in the presence or absence of IL-12 (1 ng/ml) and stimulated with various concentrations of LZ forms of ULBP1-LZ or ULBP2-LZ. Cell-free supernatants were analyzed by ELISA after 24-h incubation for the presence of IFN– + ,TNF– § and MIP-1 g . Production of all three proteins from NK cells was induced in a dose dependent manner by ULBP (Fig. 2A–C).”<br><br>“A **strong enhancing effect on IFN– g + ,TNF- a** , and MIP-1 g **production and proliferation was observed when NK cells were stimulated with ULBP in the presence of IL-12**”	[120]
		CD8_NKG2D	“In this regard, we investigated the functional capacity of TCR-stimulated **CD8+ T cells** with or without costimulation by NKG2D. Cells cultured for 7 days were restimulated with anti-CD3 mAb for 24 h in a recall assay, followed by analyses of the cytokine production profile. **Our results show that cells that were primed with anti-CD3 mAb in the presence of NKG2D costimulation tend**	[63]

			**to produce more type 1 cytokines (IFN-g and TNFa).”**	
		NK AND ((IL12 AND (IL2 OR IL15 OR IL18))	“IL-12...and it induces the NK cells to secrete IFN-γ and TNF-α[75,76]”	[70]
			**Figure 4. IL15 and IL-2 stimuhte CD56 dim NK cell cytokine production in a similar fashion.** 10^5 sorted CD56 dlm NK cells (>97% pure) were plated in U-bottom wells in 200 #1 of medium supplemented with one or more of the following cytokines: TNF-~, (300 U/ml), IL-12 (10 U/ml), I1.,2 (10 ng/ml), or IL-15 (10 ng/ml). Cells were incubated for 72 h at which time the culture supernatants were harvested and assayed for the presence of either IFN-3, (A), TNF-~ (B), or GM-CSF (C), using an ELISA. Cytokine production is measured in pg per ml”	[78]
			**Figure 3:**	[79]
			“We show that although **IL12-cultured NK cells produced** abundant IFNg, **TNFa,** and GM-CSF in response to stimuli acting on the NKp46-activating receptor, IL-4-cultured NK cells did not release detectable levels of these cytokines.”<br><br>**Figure 2:**<br>“**NK cells from donor A were cultured overnight in the presence of the indicated cytokines**, used alone or in combination. Subsequently, NK cells were stimulated with either plate-bound anti-NKp46 (b) or PMA plus ionomycin (c). After 4-h stimulation, supernatants were harvested and as-sessed for IFN-g, TNF-a, and GM-CSF (b) or for IFN- , TNF- , GM- IL-5, and IL-13 (c) by specific ELISA (n ? 3; mean ? SD). Data are representative of five different experiments.”	[80]
		Th1 AND IL12	“While **IL-12 promotes the release of IFN-γ from Th1 T cells….,”**	[40]
		IL4	“IL-12-mediated induction of IFN-g can be modulated by a number of cytokines, including **IL-4,** IL-10, and IL-18 (16, 18, 37–39).”<br><br>16 → MOUSE<br>18 → MOUSE<br>37 → Do not mention IL4<br>38 → MOUSE<br>39 → MOUSE	[75]
		(MACR OR DC) AND IL23	“In innate colitis, **IL-23 was found to control the production of a number of inflammatory cytokines such as IL-1b, IL-6, TNF-a and IFN-g** and both TNF-a and IFN-g were found to play a functional role (7). This finding is consistent **with the ability of IL-23 to induce production of these inflammatory cytokines by activated DCs and macro-phages** (34).”	[73]

			7 → T and B cell-deficient mice	[74]
			34 → "Moreover, **IL-23 stimulation could drive IL-12 and IFN-g production** at levels comparable to stimulation with IL-12 **(43).**"<br><br>**"IL-23 was reported originally to enhance IFN-g production by human memory (CD45RO) T cells.** This finding has led to some confusion regarding the importance of IL-23 in Th1 cell development and IFN-g production… However, **in human, this developmental progression does not appear to be quite** as clear cut, **as activated T cells can produce IFN-g in response to IL-23 stimulation but at far lower levels than that induced by IL-12** (9)"	[75]
		Th0_M AND (LPS OR MDP OR PGN) AND (IL12 OR IL23)	43→ MURINE<br>**9 → "Similar to IL-12, human IL-23 stimulates IFN-g production and proliferation** in PHA blast T cells, **as well as in CD45RO (memory) T cells."**<br><br>**Figure 6. "p19p40 Stimulates the IFN-g Production** and the Proliferation of Human PHA Blasts and Activated/Memory T Cells (A) PHA blasts were derived from cultured PBMC in PHA (0.1 ?g/ml)- and IL-2 (10 U/mL)- containing medium. After 7 days, cells were stimulated for 60 hr with 40 ng/ml hHy-p40- p19 or 1 ng/ml IL-12 and 1 ?g/ml soluble anti- CD28 in 96-well plates coated with 10 ?g/ ml anti-CD3 mAb. IFN-? was measured by ELISA."<br><br>**Figure c and d:**<br><br>**"fig (C and D) p19p40** has a more pronounced effect **on human CD45RO (memory)** than on CD45RA (naive) T cells. FACS-purified CD45RA and CD45RO T cells were **cultured on anti-CD3** (10 ?g/ml) and **anti-CD28** (1 ?g/ml) coated 96-well plates and stimulated with **40 ng/ml hHy-p19-p40**, **1 ng/ml IL-12** (R&D Systems), or 100 U/ml IL-2 (R&D Systems) for 60 or 136 hr. **IFN-g production (C)** and **proliferation (D) were determined** as in (A) and (B)**."**	[97]
**IFNg** = ((NK **AND** (PGN **OR** LPS **OR** MDP) **AND** (IL23 **OR** (IL12 **AND** (IL2 **OR** IL15 **OR** IL18)))) **OR** (Th0_M **AND** (LPS **OR** MDP **OR** PGN) **AND** (IL12 **OR** IL23)) **OR** Th1 **OR** ((CD8_NKG2D **OR** NK_NKG2D) **AND** (IEC_MICA_B **OR** IEC_ULPB1_6)) **OR** (Th17 **AND** (PGN **OR** LPS **OR** MDP)) **OR** ((MACR **OR** Th0) **AND** IL18 **AND** IL12)) **AND NOT** ((IFNg **AND** (TGFb **OR** IL10)) **OR** Th2))				
16	IL23<br><br>Upregulate d in CD [30]	IL1b AND MACR	"IL-1b on the other hand, can activate the release of other proinflammatory cytokines such as TNF-a, IL-23 and IL-6" **[30,71,116].**	[16]
			**30:** "Production of IL-23 in monocytes from these patients by beta-glucan is IL-1β dependent **(43)**."	[55]
			**(43)** "IL-1 system regulates IL-23 production from human monocytes" **Figure 4**	[121]

		DC	"IL-23 is predominantly produced by cells of the innate immune system, including **DCs and macrophages** in the gut"	[15]
			"...Their most potent effector functions are stimulated by sensing the **production of IL-23 by macrophages and DCs activated** in response to microbial stimulation **[57]"**	[24]
			**[57] MICE** "IL-23 and IL-1 secreted by DCs in response to microbial stimulation work together to induce IL-17A production by ILCs."	[122]
			"Secreted by activated dendritic cells and activated macrophages, IL-23 functions in innate and adaptive immunity to regulate Th17 function and proliferation. In addition, this cytokine induces CD8+ memory T cells to proliferate and produce IL-17. As such, IL-23 has been described as a key cytokine controlling inflammation in peripheral tissues. These activities are mediated by activation of the JAK/STAT pathway following IL-23 binding."	http://www.ebioscience.com/knowledge-center/antigen/il-23.htm
			**MICE** "We demonstrate that IL-23 and IL-1 secreted by DCs in response to microbial stimulation work together to induce IL-17A production by ILCs"	[123]
		MACR OR DC	"**IL-23 is expressed by macrophages** and **activated by dendritic cells in response to microbial stimulation**, to endogenous signals like prostaglandin E2, and stimulation via CD40L, a protein that is primarily expressed on activated T cells [31]."<br>[31] Review Nature.	[40]
		AND NOT (MACR AND TNFa)	41→ MICE<br>[46]<br>"To investigate the relationship between on LPS-and IL-12 production, we analyzed the effects of exposure of human monocyte-derived macrophages to<br>TNF-a on LPS, we anañyze the effects of exposure of human monocyte-derived macrophages to TNFa on LPS or SAureus induced IL12 production in the presence or absence of IFNg. "<br><br>"**TNFa is a potent inhibitor of IL12 p40 and p70 section from human macr** induced by LPS or S.aureus."<br><br>"IL10 is not responsible for the TNFa mediated inhibition of IL12."<br><br>"These data demonstrate **a selective negative regulation on IL12 by TNFa, identifying a direct negative feedback mechanism for inflammation-induced suppression of IL12 gene expression**."<br><br>"We show here that i**n human monocyte-derived macrophages (MDM)**,3 **selectively inhibits IL-12 p40 transcriptio**n, but not p35, identifying a novel regulatory mechanism of action for TNF-a -induced suppression of IL-12 production."<br><br>"In this report, **we observed that TNF-a is a potent and selective**	[124]

			**TNF-a inhibitor of IL-12 p40 and p70 production from human MDM**(Fig.1). This inhibition is observed **when MDM were pre-exposed to TNF-a before, but not at, the time of stimulation with LPS or S. aureus**”<br><br>**comment:**<br>IL23 shares p40 subunit, then TNFa inhibits also IL23 production ¿?	
		**Comment:**<br>“Antibodies to p40, it turns out, also suppress the activity of IL-23, because IL-23 shares the p40 subunit with IL-12. Thus the role of IL-12 may have been overestimated.”<br><br>“However, selective targeting of IL-23 is now emerging as an attractive concept—not only with the new findings, but also because IL-12 mediates protective systemic antimicrobial immunity.”<br><br>**Comment:**<br>“However, there is evidence that IL23 contributes to antibacterial immunity at mucosal surfaces,15 35 and further understanding of this pathway will be required to balance the beneficial effects of IL23 depletion on chronic inflammation with potential deleterious effects on host protective mucosal immunity.”<br><br>**Comment:** In the absence of IL-23, both Th1 and Th17-associated cytokines were reduced, suggesting that IL-23 promotes both types of response in the intestine (5).<br><br>**Comment:**<br>“Recent extensive genome-wide association studies on two large cohorts of CD patients (567 patients with ileal CD) **showed a correlation between several polymorphisms in the IL-23R gene locus on chromosome 1p31, and CD**: **an uncommon variant of the gene IL-23R seems to be related to a protective activity against CD** [24 These data, although the underlying mechanism behind the differential susceptibility is not clear, have lead to the evaluation of the IL-23 pathway, as a target for new therapeutic agents.”		[125]<br>[42]<br>[73]<br>[40]
**IL23** = (MACR **AND** IL1b) **OR** DC				
17	IL22<br><br>upregulated in CD<br>[126]	Th17	“**Like IL-23, IL-1b induced the expression of** IL-17A, IL-17F, **IL-22** and IFN-g **by naive CD4+ T cells. IL-23** strongly **upregulated the expression** of IL17A, IL17F, **IL22**, IL26 and CCL20.”<br><br>**Comment:** Th0 AND (IL23OR IL6 OR IL1b) is Th17.	[96]
		NK AND (IL18 AND IL12) OR IL23	“A recent publication by Cella et al. has shown that **IL-22 is also produced by human and mouse mucosal natural killer (NK-22)** cells **[18]**. This subset of NK cells is present in the **mucosa-associated lymphoid tissue** and produce IL-22 in response to IL-23.”	[127]
			**18:** “NKp441 NK cells revealed production of IL-22”	[128]

			**“NK cells produce IL-22** in response to IL-12 and IL-18 or IL-23. This activation by IL-12 and IL-18 can also induce NK cells to secrete IL-22 **(26, 27)**.”	[129]
			**27: Mice:** “**IL-12 and IL-18 induced IL-22 mRNA and protein in NK cells**, but only in the CD27 high population. On the other hand, **IL-23 was able to induce IL-22 in both subsets**.”	[130]
			**26:** “In this study **we show that IL-22 expression is specific for activated T cells and, at lower levels, NK cells in which it increases with time**.” Figure 1	[131]
			“However, IL-22 is not exclusively produced by Th17 cells, but rather appears to be produced by activated T cells in general, as well as by IL-2- or IL-12-stimulated natural killer(NK)cells **(36).**”<br>**36:** [131]	[15]
			“NK cells developed in the absence of this transcription factor (15, 16). Of note, the majority of NKp46+ RORgd+ cells were localized within the cryptopatches of the small intestine and ROR?t was required for their development, whereas RORgd+ is required for the lineage commitment of lymphoid tissue inducer cells (LTi cells)”<br><br>“NKp46+ ROR?t− cells but not NKp46+ ROR?t+ cells mediated cytotoxicity and produced IFN-g albeit at lower levels than did mature splenic NK cells (15, 16). These data suggest that **NKp46+ RORgd− cells are the LP-resident NK cell population**, whereas the **NKp46+ RORgd+ cells might have derived from LTi cells.** “<br><br>“**NKp46+ ROR?t+** cells **constitutively produce IL-22**, a cytokine regulating epithelial expression of antimicrobial proteins”	[3]
		CD4_NKG2D	“It has been shown that IL-22 is increased in active In CD and promotes proinflammatory gene expression.14 our study, a notable fraction of CD4+ T cells produced IL-22 in the mucosa and in the PB. **As observed for IL-17, the LP CD4+NKG2D+ T cells produced higher levels of IL-22 (17.3%?7.3%) than CD4+NKG2D- T cells** (6.2%?4.6%, P ? .001, n ? 10).” **Figure 6**	[62]
		(Th0 AND IL22 AND IL21) AND NOT TGFb	“In contrast to what has been observed in mice, **IL-21 also increased IL22 mRNA levels in naive CD4+ T cells in the absence of any exogenous IL-23. However, TGF-β suppressed the expression of** IL21 and **IL22 mRNA induced by IL-21**”	[132]
**IL22** = Th17 **OR** (NK **AND** ((IL18 **AND** IL12) **OR** IL23)) **OR** CD4_NKG2D **OR** ((IL22 **AND** Th0 **AND** IL21) **AND NOT** TGFb)				
18	IL21	Th17	“Th17 cells also secrete IL-21 to communicate with the cells of the immune system”	[134]

	Upregulated in CD and UC.<br><br>[30]<br>[133]		"Because Th17 cells themselves are a major source of IL-21, an autocrine amplification loop was proposed by which Th17 cells en-hance their own differentiation and precursor frequency **(5–7, 25)** (Figure 1)."	
			**6 mice: Figure a**	[135]
			**5 mice**<br>**7 mice**<br>**25 mice**	[136–138]
		NK	"IL-21 is a pleiotropic type I cytokine that is produced mainly by T cells and natural killer T (NKT) cells."	[139]
		(Th0 AND IL6) AND NOT (IL4 OR IFNg OR TGFb)	**Mice:** "**Naive CD4 + T** cells were stimulated with anti-CD3 mAb/anti-CD28 mAb in the presence of **IL-6, anti – IL-4 mAb, and anti – IFNG** mAb with or without TGFb. We found that IL-6 together with the blocking antibodies to IL-4 and IFNg strongly induced the **development of IL-21** – producing CD4 + T cells without the induction of IL-4, IFNG, IL-17A, or IL-17F production. Furthermore, unexpectedly, **TGFb inhibited the development of IL-21 – producing CD4 + T cells in a dose- dependent manner.**"	[140]
IL21 = Th17 OR ((Th0 AND IL6) AND NOT (IL4 OR IFNg OR TGFb))				
19	IL17<br><br>Upregulated in CD and UC<br><br>[30]<br>[109]	Th17	Figure 2 : **Th0 AND (IL1b OR IL23)** which is Th17	[96]
		(CD4_NKG2D AND (IEC_MICA/B OR IEC_ULPB1_6))	**"NKG2D is present on more than 70% of IL-17–positive CD4+ T** cells and almost absent in CD4+ IL-17 negative cells."<br><br>**"CD4+NKG2D+ T cells showed a strong responsiveness to Th17-promoting signals.** These results emphasize that **NKG2D strongly identifies Th17 cells in CD"**<br><br>"in HCs, NKG2D and IL-17 expressions on CD4+ T cells were significantly lower, and IL-17–producing CD4+ T cells slightly expressed NKG2D"	[62]
		Th17	"It is also well established that Th17 like Treg cells are present at elevated levels in the intestinal mucosa and in circulation of IBD patients, compared to healthy controls **[10,75–77]**."<br><br>"Taken together, these data demonstrate that inflammatory conditions in IBD can induce IFN-c or IL-17A production in a fraction of Foxp3+ Tregs in vivo, but do not affect their immunomodulatory functions"<br><br>**Comment:**<br>• Inflammatory conditions AND IL6 AND IL23 AND IL1b which	[83]

			is Th17.<br>• It is produced by Treg-Th1 cells (because of T cell plasticity) but in Th17 conditions. It has been simplified with Th17 in the Boolean function because it is not possible to write Treg because it is inhibited in this cytokine environment and this relation in the Boolean equation would not happen.	
			10 **"**IL17+FoxP3+ T cells were identified in inflamed intestinal mucosa of **patients with <u>Crohn's disease (CD)</u>**, but **not in patients with ulcerative colitis (UC**) or healthy controls."<br><br>"These cells shared phenotypic characteristics of Th17 and Treg cells, and showed potent suppressor activity in vitro. Transforming growth factor-® was necessary and sufficient to induce development of a IL17+ FoxP3+ cell population in CD4+ LPLs derived from patients with UC"<br><br>"TGF-β is an essential factor in the development of FoxP3+IL-17+ cells, and that this population originates from FoxP3+ precursor cells in a distinct inflammatory microenvironment."	[119]
			Recently, it has been demonstrated that Tregs in healthy humans are able to produce IL-17A after activation [15,16].	[141]
		Th17_M	Figures b (Central memory T cell):<br>**Th0_M AND (IL23 OR IL1b OR IL6)** and this is equal to Th17_M	[82]
			**"IL-1b and IL-6** induced during the **early stages** of an inflammatory response may act on **memory T cells** to promote **IL-17** and **IL-21** secretion"	[96]
		CD4+T OR NKT OR CD8+T OR gdT	"There are other immune cells that also produce IL-17, such as gammadelta T cells [10, 19, 26], innate Th17 (iTh17) [27], **natural killer (NK)** cells, mast cells, and neutrophils **[10, 19]**."	[142]
			19 Do not mention	[143]
			10 **"**In addition to T cells, other cellular sources of IL-17 include **natural killer (NK) cells**, mast cells and neutrophils"<br>"**iNK** cells **that produce IL-17A** have been shown to express neuropilin 1 (55) and are recent thymic emigrants, suggesting that they may be genetically programmed to produce IL-17A."<br><br>"IL-17 can also be produced by several other innate immune cell types, such as lymphoid tissue inducer cells, natural killer and natural killer T cells, macrophages and Paneth cells (reviewed in references **39 and 40**). The functional importance of the IL-17 produced by these cell types during inflammation is not very well characterized."	[144]

			39 "A role for NK cells in exhibiting certain Th17-like factors has previously been described. In certain situations NKp44+ NK cells produce high levels of innate IL-22 (93). Additionally, NK cells can express high levels of the transcription factor RORγ (94). **Thus, it seems plausible that NK cells would be another innate source of IL-17 and IL-17F,** especially considering their developmental similarity to LTi cells. **However, only one report has demonstrated that NK cells have this potential. In a model of toxoplasmosis, NK cells were able to generate IL-17 through a mechanism that was dependent on IL-6 (95)**. Thus, further assessments of NK cells as IL-17 producers are warranted."<br><br>"Of particular importance are the invariant NKT cells **(iNKT)** that express a single restricted TCR receptor and are activated by αGalCer. These cells were also shown to **produce IL-17 in an innate capacity following TCR ligation and IL-23 stimulation (97). iNKT cells did not, however, require IL-6 to induce IL-17**."<br><br>"Furthermore, CD4-/NK1.1**+ NKT T cells were recently described as being the most potent IL-17 producers compared to the CD4**+ and NK1.1+ NKT subsets (98)."<br><br>**Table 1 and 2**<br><br>**<u>Comment:</u>** CD4+T OR NKT OR CD8+T OR gdT not in the network	[145]
		AND NOT IL13	"Conclusions—IL-13Rα1 is expressed on human CD4+ Th17 cells, and IL-13 attenuates IL-17A production at polarization and restimulation. While IL-13 is an attractive therapeutic target for decreasing symptoms associated with asthma, these results suggest that therapies inhibiting IL-13 production could have adverse side effects by increasing IL-17A production."<br>**Figure 13**<br><br>"IL-13 attenuated IL-17A levels in human CD4+ Th17 cells, but the amount of IL-13 necessary for inhibiting IL-17A production was greater in humans than in mouse Th17 cells, in which 5ng/ml of IL-13 attenuated IL-17A production.8"<br><br>"This study shows that IL-13 is important in negatively regulating Th17 cytokine production. However, further studies need to be conducted to determine the precise mechanisms for IL-13 inhibiting IL-17A production in human Th17 cells. In contrast, IL-13 may provide a potential therapeutic for treatment of Th17 mediated diseases, including Crohn's disease and rheumatoid arthritis, by decreasing IL-17 production."<br>**Figure 4**	[146]
		AND NOT TGFb	**Figure 1**: "A **combination of TGF-b and IL-6 reduced** the IL-23-driven production of **IL-17A,** IL-17F and IL-22".	[96]

			Inhibits production. Downregulates in a concentration dependent manner:<br>"**At different concentrations of TGFb1** (10, 1, 0.1 and 0.01 ng/ml) The mean production of IL17 was, respectively, 67.7%, 61.2%, 89.6% and 86.2% of that measured in the presence of medium only"	[109]
		Th17 AND IL23	"..**IL-23 binds to Th17 T cells and macrophages, promoting the release of IL-17, IL-6, IL-1 and TNF**.<br><br>"The difference between the heterodimeric complex binding IL-12 and IL-23 confers to **IL-23 a specific intracellular signaling, with consequent lymphocyte activation and cytokine, mainly IL-17 production** [23]"<br><br>~~Th17 AND IL23 →~~ IL23 induces Th17, then once it is active IL17 would be secreted	[40]
**IL17** =Th17 **OR** (Th17_M **AND** (LPS **OR** MDP **OR** PGN)) **OR** ((CD4_NKG2D **AND** IEC_MICA/B **OR** IEC_ULPB1_6)) **AND NOT** ((TGFb **OR** IL13) **AND** IL17)				
20	IL10<br><br>Altred:<br><br>[147]:<br><br>Wang et al[18] Upregulated in CD<br><br>Kucharzik et al[82] Upregulated in CD or UC<br><br>Mitsuyama et al [85] Upregulated in UC<br><br>Nielsen et al[81] not altered<br><br>[148]	(TLR2 AND NFkB) AND NOT (MACR AND IFNg)	"**TLR2 is a potent inducer of anti-inflammatory interleukin-10** (IL-10), which critically **inhibits multiple macrophage and dendritic cell (DC)** effector functions, thus limiting exaggerated immune responses. Downstream recruitment of the adaptor proteins Mal or MyD88 lead to diverse immune responses through cytokine production (such as anti-inflammatory interleukin-10 (IL-10)) **via transcriptional activation of nuclear factor-kB (NFkB)**."<br><br>"**Presence of a CARD15 mutation** -which has been shown to be associated with increased Crohn's disease susceptibility-imbalances TLR2-mediated NFkB activation in antigen presenting cells, thus leading to mucosal inflammation through exaggerated interferon-g , IL-12, and IL-23 production, 29 which in turn **may further suppress mucosal TLR2-mediated anti-inflammatory IL-10 production via paracrine loops.** 30"	[14]
			"**IFN-g inhibits IL-10** production by regulating the activity of GSK3 and MAPKs downstream of TLR2"<br><br>"Taken together, our results support a model whereby **IFN-g suppresses IL-10 production by two mechanisms:** (1) IFN-g-dependent suppression of MAPKs and (2) IFN-g activation of GSK3"<br><br>"**TLR2 stimulation induced the time-dependent production of substantial amounts of IL-10** in control cultures (Figure 2A, top). In striking contrast, TLR2 stimulation of IL-10 production **was strongly suppressed in IFN-g-activated macrophages**"<br><br>**Comment:** ~~AND TLR2~~ Can be suppressed from the Boolean function because TLR2 is needed for the activation, thus the inhibition is going to take place only if something is activated, and IL10 is	[149]

	**KAT2B down-regulation in IBD:** - Downregulated		activated in the presence of TLR2.<br><br>TLR2 AND NOT (MACR AND IFNg ~~AND TLR2~~)	
		Treg	**Mice:** "**Regulatory T cells (Treg)**, Tr1 cells, and/or Th3 cells **produce IL-10**"	[111]
			"Treg are crucially involved in the maintenance of gut mucosal homeostasis by suppressing abnormal immune responses against the commensal flora or dietary antigens. In particular, Treg exert their function by producing the anti-inflammatory cytokines IL-10 and TGF-β and by preventing both the activation and the effector function of T cells that have escaped Innate and adaptive i other mechanisms of tolerance **[81]**"	[150]
			**[81]** "CD4?CD25hi T cells proliferated poorly to immobilized anti-CD3 (Figure 2A), and produced no detectable amounts of IL-2, IL-10, IL-4, TGF?, IFN?, or IL-6 (data not shown)."<br><br>**Comment**: assuming that in normal conditions Treg produced IL10	[151]
		Th2 AND NOT IL23	"Interleukin (IL)-23 suppresses IL-10 in inflammatory bowel disease."<br>"IL-23 Suppresses Expression of IL-10 in Th2 Cells."<br>"The **data suggest a potential that the decrease in IL-10 in the IBD colon mucosa may be induced by the increase in IL-23**"	[152]
		Th17 AND NOT IL23	"There is evidence that **differentiation of Th17 cells in the absence of IL-23 leads to Th17 cells producing IL-10**, an anti-inflammatory cytokine, which are therefore poor inducers of inflammation **23**"	[113]
			**23** All in mice.	[153]
		IL10 AND (Treg AND IL2)	"We recently demonstrated that **IL-2 enhanced IL-10** production through **STAT5 activation**."<br><br>We do not include:<br>**OR** (IL10 **AND** Treg **AND** IL2)<br>Because Treg already activates IL10. The upregulation will do nothing	[154]
		(MACR AND LPS) AND NOT IL4	"IL-4 strongly inhibited IL-10 production by LPS activated monocytes"	[35]
		DC AND LPS	"In cells from control tissue, the proportion of IL-12–producing DCs was variable and low; overall, it did not reach statistical significance. There were no detectable IL-6–producing DCs. However, a **significant proportion of colonic DCs from healthy tissue produced IL-10. Figure**	[38]

<table>
<tr><td></td><td></td><td></td><td>Mice "The ambiguous Th2/Treg marker cytokine IL-10 was detectable only when stimulated DCs were used for T cell stimulation. In case of stimulated ROF-DCs, IL-10 levels were much lower than observed for cocultures that contained LPS-stimulated control BM-DCs."
● ROF-DCs: PDE4 inhibitor roflumilast (ROF, trade name: Daxas)
● BM-DCs: bone marrow-derived (BM-) DCs

Comment: bone marrow-derived (BM-) DCs produce IL10 when they are stimulated.</td><td>[155]</td></tr>
<tr><td></td><td></td><td colspan="2">To examine the molecular consequences of KAT2B down-regulation in IBD:
-IL-1β, IL-6, and IL-18 were significantly up-regulated
-whereas IL-4, IL-8, and IL-10 were significantly down-regulated in a dose-dependent manner.
-IL-13, IL-17A, IL-23A, and TNFα expression was not influenced by KAT2B</td><td>[148]</td></tr>
<tr><td></td><td></td><td colspan="2">Comments:
"Studies have been inconsistent regarding serum levels of IL-10 in IBD, as stated earlier some studies show higher IL-10 levels in CD, Wang et al[18] found that CD patients had significantly higher levels of IL-10 compared to controls. Kucharzik et al[82] reported increased serum IL-10 concentrations in patients with active CD or UC compared to controls. Mitsuyama et al [85] showed an increase in serum IL-10 in active UC patients but not CD. In contrast, Nielsen et al[81] concentrations did not differ among UC, CD and healthy control subjects."

"However, to date, the results of the clinical trials have been disappointing. Although CD activity was reduced as measured by the CD activity index, IL-10 supplementation did not result in significantly reduced remission rates or clinical improvements when compared to placebo"

"Table 1 Summary of key findings from interleukin-10 trials in human and animal studies"</td><td>[147]</td></tr>
<tr><td colspan="5">IL10 = Treg OR (Th2 AND NOT IL23) OR ((TLR2 AND NFkB) AND NOT (MACR AND IFNg)) OR ((MACR AND LPS) AND NOT IL4) OR (DC AND LPS)</td></tr>
<tr><td rowspan="2">21</td><td rowspan="2">Th17

Upregulated in CD and UC
[156]

[157]</td><td colspan="3">EMAIL: rene.de.waal.malefyt@merck.com related to their work [96]
"Following this initial paper, we have conducted many more studies that were published by Katia Boniface and Melanie Kleinschek in JI and JEM. We found consistently that using our culture conditions both IL-1 and IL-23 are able to induce Th17 differentiation. We also showed that there are many different subtypes of TH17 cells.
From our first paper on we have never seen a positive effect of TGFb on human Th17 differentiation. TGFb is mainly immunosuppressive and inhibits T cell responses and differentiation. Later on it was demonstrated that Th17 differentiation in the mouse could also occur in the absence of TGFb :
http://www.nature.com/nature/journal/v467/n7318/abs/nature09447.html
As far as I know, no one has been able to repeat the results from Yang."</td></tr>
<tr><td>IL2</td><td>"IL-2 will ultimately lead to the population expansion of TH-17 cells" ([96], [39]):</td><td>[158]</td></tr>
</table>

			• [96]: Do not show it	
			[39]: Several cytokines, including type I and type II interferon, IL-4, IL-12, IL-27 and **IL-2,** inhibit TH-17 differentiation 1,2,4,18–20.	[39]
			1: MICE	[159]
			2: MICE	[160]
			18: In addition, IL-2 and IL-15 also induce IL-17 production in human PBMC [24].<br>We show that not only IL-2 and IL-15, but also IL-23, IL-18 and IL-21 stimulate IL-17 production by human T cells.**¿?**	[161]
		IL1b OR IL23	“**IL1b and IL23** were able to drive human **naive CD4+** T cells toward a Th17 phenotype”	[96]
			“I**L-6 did not enhance this apparently spontaneous TH-17 differentiatio**n, whereas **TGF-b suppressed the production of both IL-17 and IFN-g. Also, IL-1b strongly induced the differentiation of IL-17-producing cell”**	[39]
			**MICE:**<br>**39:** “The development of TH-17 cells from naive precursor cells was potently **inhibited by interferon-g (IFN-G) and IL-4,** whereas committed TH-17 cells were resistant to suppression by TH1 or TH2 cytokines. **In the absence of IFN-g and IL-4, IL-23 induced** naive precursor cells to differentiate into TH-17 cells independently of the transcription factors STAT1, T-bet, STAT4 and STAT6.	[160]
		TGFb and IL21	“TGF-β and IL-21 was uniquely able to induce TH17 differentiation.”<br>**Do not: email Rene**	[132]
		IL21 AND TGFb	IL-21 drives differentiation of naiıve T cells into Th17 cells.<br>[35-38(humanos)]	[142]
			Promotes differentiation	[15]
		IL6	“IL-6 induces the generation of Th17 cells from naı¨ve T cells together **with TGF-b** and inhibits TGF-b-induced Treg (iTreg) differentiation [13–15].”	[162]
			Promotes differentiation(**needs TGFβ)**	[15]
		AND NOT TGFb	“In contrast to what might have been predicted based on experiments in mice, **TGF-b and IL-6 did not induce TH-17 differentiation and even blocked IL-23-induced development of TH-17 cells**”	[96]<br>[163]

			“TGF-β **suppressed** the production of both IL-17 and IFN-g.”	[39]
			TGF-β inhibits IL-17 production<br>• 1. Reference Wilson<br>• 2. A costa RodrigueZ	[158]
		AND NOT Treg	“Regulatory T cell **(Treg) generation** (depicted by the cells shown in red) and thus counter-acts the inhibitory effect on Tregs on both Th1 (IFN-γ) and Th17 (IL-17) pro- inflammatory responses.”<br><br>“This is seen in the fact that induction of Foxp3 expression, the signature protein of regulatory T cells, inhibits RORγt function, the main IL-17 transcription factor. Similarly, the induction of Th17 cells inhibits Foxp3 expression” 25,26	[113]
			25: High concentrations of TGF-β, together with retinoic acid (RA), may be required for induction of **Foxp3+Tregs** to s**uppress potentially detrimental inflammatory Th17 cell responses** [57-62].	[164]
		AND NOT IL12<br><br>AND NOT IL4	“IL-12, IFN-g, and IL-4 suppress Th17 cell differentiation [22, 23, 39]”<br>• ref 22: MICE<br>• ref 23:MICE<br>• ref 39: MICE:<br>“The development of TH-17 cells from naive precursor cells was potently **inhibited by interferon-g (IFN-G) and IL-4,** whereas committed TH-17 cells were resistant to suppression by TH1 or TH2 cytokines. **In the absence of IFN-g and IL-4, IL-23 induced** naive precursor cells to differentiate into TH-17 cells independently of the transcription factors STAT1, T-bet, STAT4 and STAT6.”	[111]<br>ref 22:[165]<br>ref 23:[166]<br>ref 39:<br>[160]
		AND NOT IFNg	“Th1 and Th2 cells are known to antagonize each other, and not surprisingly, IL-12, IFN-γ, and IL-4 can inhibit Th17 differentiation in both mouse and human (**2, 3, 17, 19, 20, 25, 26**).”<br>**2 → mice**<br>**3 →mice**<br>**19 → mice**	[167]
			**17** →”Even the neutralization of IL-4 produced by Th2 is critical in neutralizing the development of IL-17; however, neither IFN-γ nor IL-4 seem to be effective on already established Th17 (20).”	[168]
			**20** → “both IL-12 and IL-4 prevented IL-23-induced TH-17 development **(Fig. 1e).**”	[169]

			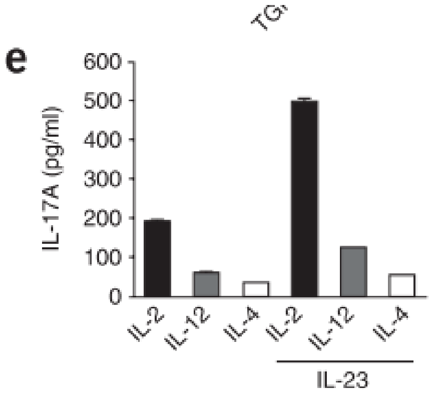	
			26 →"IL-23 enhanced IL-17 secretion, as did IL-2, IL-15, IL-18 and IL-21. **In contrast, IL-12 mediated specific inhibition of IL-17 productio**n."	[161]
		Th17 AND IL23	"IL-23 primarily induces proliferation and survival of Th17 cells."	[70]
			"IL-23 specifically induces the differentiation of naïve CD4+ T cells into Th17 cells, with the activation of a cascade of several pro-inflammatory cytokines: IL-17, IL-17F, IL-6, and TNF-alpha [25"<br><br>"Since the cytokine microenvironment can modify the behavior of some T helper (Th) subtypes and there is an overlap between Th1 and Th17 pathways, a precise differentiation between the specific effects exerted by IL-12 and IL-23 has not been defined [30]."	[40]
		Th0 AND IL23	In chronic inflammation, antigen-stimulated dendritic cells and macrophages produce IL-23, which promotes the development of Th17/ThIL-17 cells	[43]
		**<u>Th1 does not difference between CD from HC</u>**<br>**<u>Th17 Upnregulated</u>**<br>**<u>Th17/Th1 Upnregulated</u>**<br><br>"A larger proportion of commensal-specific CD4+ T cells from patients with CD have a Th17 phenotype or produce Th1 and Th17 cytokines, compared with T cells from controls; this might contribute to intestinal inflammation in patients with CD."<br><br>"To define the cytokine profile of microbial antigen-driven T cell responses, we first **measured IFN-γ and IL-17 in culture supernatants of total PBMCs stimulated for 7** days with TT, ASCA Ag, CBir1, FlaX, A4-fla2, and YidX. **We looked at IFN-γ and IL-17 as they are characteristic of Th1 and Th17 response,** respectively, **both of which have been associated with gut inflammation in CD** 28. **Both cytokines were significantly increased in CD compared to control supernatants** in response to FlaX, A4-fla2, and YidX (Supplementary Figure 4A-B)."<br><br>"**While the percentage of single IFN-γ+ IL-17- cells (Th1) was similar for CD patients patients and controls** (Figure 2B), **the frequency of IL-17-producing T cells was significantly higher in the former**. Remarkably, we noted significantly higher		[157]

		frequencies of IL-17+ IFN-γ- single (Th17) and IL-17+IFN-γ+ double-positive (Th17/Th1) CD4+ T cells that recognized FlaX single (Th17) and IL-17+IFN-γ+ double-positive (Th17/Th1) CD4+ T cells that recognized FlaX, A4-fla2 and YidX in CD patients compared to control individuals (Figure 2C-D). **These data indicate that commensal-specific CD4+ T cells in CD patients present a Th17 and Th17/Th1 phenotype upon antigen recall that is not observed in T cells responding to the same antigen in healthy controls"**		
		**Upregulated in CD and UC:**<br><br>"**the prevalence of IL-17A-producing Th17 cells was increased in both IBD cohorts (Fig. 3C), as were CD25 and FoxP3 double-expressing or FoxP3 single-expressing Treg (Fig. 3D) compared with HC.** However, no Significant differences in the prevalence of Th17 or Treg cells were observed between the 2 IBD cohorts **(Fig. 3C, D).**"		[156]
**Th17** = ((Th0 **AND** (IL1b **OR** IL23 **OR** IL6)) **OR** (Th17 **AND** IL23)) **AND NOT** ((TGFb **OR** IL12 **OR** IL4 **OR** IFNg **OR** Treg) **AND** Th17)				
22	Th17_M<br><br>Overexpres sion in CD [94]	IL23	"Here we demonstrate that human naive CD4+ T cells develop into TH-17 cells in response to IL-23 or IL-1b."<br><br>"we identified **TH-17** cells in situ as a subpopulation of **CD4+ memory T cells** expressing the **IL-23 receptor (IL-23R)**."<br><br>**Figure 3**	[96]
		IL2	"These data are consistent with another study showing that **IL-17-producing memory T** cell populations **are expanded by IL-2** in both species" 13.	[158]
			**13:** "We also found, by an intracellular cytokine analysis assay, that the IL-17 expressing cells were predominantly CD4+ T cells (Fig. 1d) with a memory phenotype. **Fig. 1e:** "Detection of IL-17– and IFN-g–expressing naive (CD62L) or memory (CD45RO) cells in human CD4+ T-cell cultures propagated **in the presence or absence of IL-2"**	[170]
		Th0_M AND (IL1b AND IL6)	"IL-1b and IL-6 induce IL-17A secretion from human central memory CD4+ T cells.."	[132]
		"**The proportion of blood T cells that were CD45RA− memory T cells was significantly higher in patients and siblings** compared with controls, **figure 1A."**		[94]
**Th17_M** = (((Th0_M **AND** (PGN **OR** MDP **OR** LPS)) **AND** ((IL1b **AND** IL6) **OR** IL23 **OR** IL2))) **OR** Th17_M				
23	Th1	IL12	"A cytokine initially identified as IL-12, the **master cytokine driving the Th1 response 4,5"**	[113]

	Downregulated<br><br>[156]<br><br>Upregulated-equal<br>[157]		4: MICE<br>5:MICE: "IL-12 promotes Th1 cell differentiation and cell-mediated immunity. In the present study, the potential role of IL-12 was analyzed in an experimental colitis model in scid mice"	[171]
			"When T cells were stimulated with **IL-12** and **IL-18 in** the presence or absence of anti-CD3, **only those T cells stimulated by engagement of the T-cell receptor (TCR) could develop into Th1** cells [62]."	[101]
			"Thus, **IL-12 can promote the differentiation of Th1** cells, and IL-6 and TGFb or IL-23 can promote the differentiation of Th17 cells [9–13]."	[111]
			"In the same culture conditions, **IL-12** inhibited TH-17 differentiation but **promoted TH1 differentiation** (Fig. 1a,b)"	[39]
		IFNg	**"IFN-γ** and **IL-12 initiate** the **differentiation of Th1** cells that are characterized by high production of IFN-γ and are indispensable for clearing intracellular pathogens."	[15]
			**Figure 3:**<br><br>"**Human IFNG expression** by (B) **primary and (C) effector un-polarized (Th0), Th1 and Th2 cultures from** BACdel1, BACdel2 or the **full-length human 190 kb BAC transgene**. Results are expressed as mean ± the SEM of human IFNG expression relative to murine Gapdh expression."	[172]
		AND NOT Treg	"Regulatory T cell **(Treg)** generation (depicted by the cells shown in red) and thus **counteracts the inhibitory effect on Tregs on both Th1** (IFN-γ) and Th17 (IL-17) proinflammatory responses."	[113]
			"while T**REG can inhibit both TH1 and TH2** induction (Gorelik et al., 2000, 2002)."	[173]
		IL18	**"IL-18** is an important **mediator in the Th1 response,** primarily by induction of IFN-γ secretion from T cells and natural killer (NK) cells" **[33,34]**	[174]
			**33: MICE**<br>"This observation of **IL-12 synergy with IL-18** is consistent with the observations of o**thers who claimed that IL-18 alone was able to drive the TH1 response independently of IL-12,** 21,28"	[104]
			**34:** Review. Mouse	[101]
			"When T cells were stimulated with **IL-12** and **IL-18** in the presence or absence of anti-CD3, **only those T cells stimulated by engagement of the T-cell receptor (TCR) could develop into Th1** cells **[62]**."	[101]

			[62] "Like these NK cells, T cells produce IFN-g in response to IL-12 and IL-18 without their TCR engagement or development into Th1 cells."	[175]
		IL1b OR IL21	Fig 1b	[132]
		IL10	"The **suppressive function of IL-10** involves the inhibition of macrophages and DCs and **direct suppressive effects on Th1 cells"[18].**	[176]
			**18 MICE:** "**Interleukin-10 (IL-10)** was first described as cytokine synthesis inhibitory factor (CSIF) (1), an activity produced by mouse Th2 cells that **inhibited activation of and cytokine production by Th1 cells**."<br><br>"the effects of IL-10 on DC are consistent with **inhibition of Th1 inflammatory response**s and can be achieved by inhibitory effects on "inflammation-inducing DC" or by induction of anti-inflammatory T cell populations by IL-10-producing DC"	[177]
		AND NOT ((L23 OR IL17) AND IL12)	"IL-17 signaling via these receptors inhibit Th1 differentiation by suppressing expression of T-bet, a factor necessary for Th1 T cell developmenT. 46"	[113]
			**46: MICE** Figure 2: IL-17 modulates TH1 differentiation.	[178]
			"whereas I**L-23 suppressed Th1 cell differentiation**"<br>"IL-23 or IL-17 can suppress Th1 cell differentiation in the presence of exogenous IL-12."	[111]
		IL4	**"**IFNg suppressed TH2 and TH17 development, **IL-4 inhibits the TH1** and TH17 lineages (Harrington et al., 2005; Park et al., 2005; Yamane et al., 2005)"	[173]
		TGFb	**"TGF-β suppresses the functions of Th1** and Th2 CD4+ effector cells and NK cells, and promotes the generation of Treg cells. To promote immune responses, TGF-β induces the generation of Th17."<br><br>"we have found that **TGF-β potently inhibits effector functions of activated T** cells and thus t**heir differentiation into Th1** or Th2 effector cells under tissue culture conditions (76)."	[179]
			"Thus, i**n humans, TGF-b** seems to **inhibit the three main pathways (TH1**, TH2 and TH-17) of effector T cell **differentiation.**"	[39]
		Th0 AND IL12	"IL-12 induces activated T-cells to differentiate into IFN-γ producing Th1 cells and it induces the NK cells to secrete IFN-γ and TNF-α[75,76]"	[70]

		Th0 AND DC AND IL12 AND IL23 AND LPS	"Bacterial-derived products [e.g., **lipopolysaccharide (LPS) from** Gram-negative and **muramyl dipeptide (MDP) from Gram-positive bacteria**] are proposed to play a pivotal role in the generation of neurological and neuroinflammatory/immunological responses during bacterial infections of the nervous system."	[180]
			**GnB: Gram-negative bacteria**<br><br>**"The elevated expression levels of IL-23 and IL-27 may suggest that these cytokines may act as a cofactor, together with IL-12, in the induction of Th1** responses by **GnB-primed moDC**."<br><br>"This study demonstrates that **GnB have a clear immuno-modulatory effect on DC by the imprinting of a strong Th1 polarizing capacity**. This capacity **is only partly dependent on the activity of the classical Th1 polarizing cytokine IL-12, and it is suggested that this Th1 polariza-tion may as well be driven by the action of the novel IL-12 family members IL-27 and/or IL-23."**<br><br>**"Intestinal GnB but not GpB prime moDC for a high Th1 polarizing capacity"**	[181]
			"In addition, NFκB activation of APCs (including dendritic cells) by microbial adjuvants in**duces the expression of** MHC class II antigens, co-stimulatory molecules, **IL-12 and IL-23, which can activate TH1** and TH17 cells, **respectively,** if the appropriate antigen is present." → IL12<br><br>**Figure 3:**	[182]
		**Th1 does not difference between CD from HC**<br>**Th17 Upnregulated**<br>**Th17/Th1 Upnregulated**<br><br>"A larger proportion of commensal-specific CD4+ T cells from patients with CD have a Th17 phenotype or produce Th1 and Th17 cytokines, compared with T cells from controls; this might contribute to intestinal inflammation in patients with CD."<br><br>"To define the cytokine profile of microbial antigen-driven T cell responses, we first **measured IFN-γ and IL-17 in culture supernatants of total PBMCs stimulated for 7** days with TT, ASCA Ag, CBir1, FlaX, A4-fla2, and YidX. **We looked at IFN-γ and IL-17 as they are characteristic of Th1 and Th17 response,** respectively, **both of which have been associated with gut inflammation in CD** 28. **Both cytokines were significantly increased in CD compared to control supernatants** in response to FlaX, A4-fla2, and YidX (Supplementary **Figure 4A-B)."**<br><br>"**While the percentage of single IFN-γ+ IL-17- cells (Th1) was similar for CD patients patients and controls** (Figure 2B), **the frequency of IL-17-producing T cells was significantly higher in the former**. Remarkably, we noted significantly higher frequencies of IL-17+ IFN-γ- single (Th17) and IL-17+IFN-γ+ double-positive (Th17/Th1) CD4+ T cells that recognized FlaX single (Th17) and IL-17+IFN-γ+ double-positive (Th17/Th1) CD4+ T cells that recognized FlaX, A4-fla2 and YidX in		[157]

		CD patients compared to control individuals (Figure 2C-D). **These data indicate that commensal-specific CD4+ T cells in CD patients present a Th17 and Th17/Th1 phenotype upon antigen recall that is not observed in T cells responding to the same antigen in healthy controls"**		
		<u>**Th1 Downregulated**</u><br><u>**Th2 Upnregulated in UC**</u><br><br>● "An i**ncreased IFN-g-producing or T-bet-expressing Th1 cells** (Fig. 3A) and a **decreased IL-13-producing or Gata3+-expressing Th2 cells** (Fig. 3B) **in patients with CD were observed compared with patients with UC**."<br><br>● "Interestingly, the prevalence of IFN-g+ or T-bet+ **Th1 cells were significantly decreased in both patients with UC and CD in comparison with HC (Fig. 3A)."**<br><br>● An **increased IL-13-producing or Gata3-expressing CD4+ Th2** cells were found in patients with **UC**, **but not in patients with CD, when compared with HC (**Fig. 3B). Interestingly, the prevalence of IFN-g+ or T-bet+ **Th1 cells were significantly decreased in both patients with UC and CD in comparison with HC (Fig. 3A).**		[156]
**Th1** = (Th0 **AND** **(**(IL12 **OR** IFNg **OR** IL18) **OR** (DC **AND** IL12 **AND** IL23 **AND** LPS))) **AND NOT** ((**U**IL17 **AND** **U**IL12) **OR** ((Treg **OR** Th2 **OR** TGFb **OR** IL10 **OR** IL4) **AND** Th1))				
24	Th2<br><br>Upnregulated in UC<br><br>[156]	Th2 AND IL4	"IL-4 triggers the differentiation of Th2 cells."<br><br>"however, t**he effector cytokines that are subsequently produced by Th1 and Th2 cells** (i.e., IFN-γ and IL-4) **can potentially feed back to amplify Th1 and Th2 cells** and **further enhance differentiation of the respective T cell subset."**	[15]
			"IL-4, a member of the g-chain receptor cytokine family, is considered a crucial mediator of CD4? Th2 T cell differentiation and suppression of IFN-g-producing CD4+ Th1 cells **[1]"**.	[183]
			**[1]** "IL-4 such cells to develop into cells capable of producing IL-4 and a series of other cytokines including IL-5, IL-10 and IL-13 (i.e. TH2-like cells) **(7, 8)**."<br><br>**7, 8** → MICE	[184]
			"Interleukin-4 (IL-4) promotes a Th2 response in lymphocytes **[14]**."	[185]
			**[14]** Mouse: nothing.	[186]

			“Conversely, **cells activated in the presence of IL-4 down-regulate the IL-12 receptor and mature along the Th2 pathway**, activating the STAT-6 **[15],** C-maf **[16]** and GATA-3 **[17]** **t**ranscription factors.”	[187]
			**[15]** **“mice** deficient in Stat6 by gene targeting…”<br>**[16]** “Murine Th1 (D1.1, OF6, Ar5, AE7, and S53) and Th2”<br>**[17]** “In transgenic mice…”	[188–190]
		AND NOT Treg	“while **Treg can inhibit both TH1 and TH2** induction **(Gorelik et al., 2000, 2002).**”	[173]
			**(Gorelik et al., 2000, 2002).** → Mice (references explained below).	[191,192]
		NOD2 AND PGN	“demonstrating that **Nod2-mediated PGN recognition** elicits Ag-specific T and B cell immunity with a **predominant Th2 polarization** profile.”<br><br>“Taken together, o**ur findings show that Nod2 preferentially skews the immune response toward a Th2 profile**”<br>Mice cells<br><br>**Comments:**<br>• NOD2 recognize PGN?<br>• Mice presents a different Th differentiation pathway	[99]
		IL10	**“IL-10 promotes Th2 responses in vivo”** **(Agrawal et al., 2003; Dillon et al., 2004; Pulendran et al., 2001; Redecke et al., 2004)**	[59]
			**Pulendran et al:** **“**Good candidates for **Th2-inducing cytokines are IL-10 and IL-4**. **However, significant levels of IL-10 or IL-4 could not be consistently detected in these cultures** (data not shown).”	[193]
			**Dillon et al., 2004: MICE:** “In summary, the present data suggest that Pam-3-cys induces lower levels of the Th1-inducing cytokine IL-12(p70), a**nd much higher levels of the Th2-inducing IL-10, than E. coli LPS”**	[194]
			**Agrawal et al., 2003: “**IL-10, a regulatory cytokine that **is known to dampen both Th1 and Th2 responses in humans (6)**,” **?¿¿?**	[195]
			**Redecke et al., 2004:** Nothing related to Th2 induction by IL10	[196]
			“Like IL-10, IL-19 also promotes an increase in Th2 to Th1 T lymphocyte ratios **[25].”**	[185]

			[25] **“T-cell activation in the presence of elevated levels of IL-10** such as those found in the periodontal lesion, systemic lupus erythematosus and the rheumatoid joint, l**eads to incomplete maturation of Th1 cells** and skewing towards the IL-10 dependent Tr1 subset [4,5].”	[187]
			“IL-12 and IL-10, respectively, stimulate Th1 and Th2 immune responses.” → **It is an assumption. no references. Paper related to histamine**.<br><br>“In humans, **initial clinical trials demonstrated that IL-10** administration ameliorated inflammatory symptoms associated with endotoxemia (10), **inflammatory bowel disease (11)**, and rheumatoid arthritis (12).”	[197]
		AND NOT IFNg	“IFN-γ and IL-4 antagonize each other on different levels, and thus **Th1 and Th2 development is considered mutually exclusiv**e”<br><br>“however, t**he effector cytokines that are subsequently produced by Th1 and Th2 cells (**i.e., IFN-γ and IL-4) **can potentially feed back to amplify Th1 and Th2 cells** and **further enhance differentiation of the respective T cell subset.”**	[15]
			**“IFNg suppressed TH2 and TH17 development**, IL-4 inhibits the TH1 and TH17 lineages **(Harrington et al., 2005; Park et al., 2005; Yamane et al., 2005)”**	[173]
			**Harrington et al., 2005** → IL17<br>**Park et al., 2005** → IL17<br>**Yamane et al., 2005** → Mice	[198]
		(IL18 AND IL4) AND NOT IL12	“I**n the absence of IL-12**, **IL-18 drives the Th2 response** by inducing IL-4, resulting in increased numbers of IL-4 positive cells **from NK T cells** [30]”	[16]
			**30:** For example, **IL-18 induces IL-4, resulting in increased numbers of IL-4-positive cells** from **activated natural killer (NK) T cells in the absence of IL-12**. **→ Without reference**	[105]
			**MOUSE** “moreover, **naive T cells can develop into Th2** cells and produce both IL-4 and IL-13 in response to stimulation with TCR engagement **after being stimulated with IL-2, IL-18 and Ag**”	[101]
		AND NOT TGFb	**“TGF-β suppresses the functions of Th1 and Th2 CD4+ effector cells** and NK cells, and promotes the generation of Treg cells. To promote immune responses, TGF-b induces the generation of Th17”<br><br>“we have found that **TGF-β potently inhibits effector functions of activated T cells and thus their differentiation into Th1 or Th2** effector cells under tissue culture conditions **(76).**”	[179]

			"While the detailed mechanism remains unknown, our and others' studies showed that **T-bet and Gata-3 expression is inhibited by TGF-β (32,33,77,78)**, possibly, in the latter case, through a mechanism via blocking Itk kinase activity and calcium influx (31)."	
			**(76)** "in the presence of , and CD4+ TGF-β do not acquire CTL function 16 T cells fail to become TH1 or TH2 cells **14,15** (FIG. 1)."	[191]
			**14:** "TGF-P inhibited the development of T cells that could produce the Th2 cytokines IL-4 and IL-5." **→ MICE**<br>**15→ MICE**	[199]
			**32→ Th1**<br>**33** → MICE<br>**77**→ MICE	**33:** [192]
			"Thus, i**n humans, TGF-b** seems to **inhibit the three main pathways (TH1**, TH2 and TH-17) of effector T cell **differentiation.**"<br><br>"Finally, **the finding that in humans, TGF-b inhibits TH-17 as well as TH1 and TH2 differentiation** suggests particular caution should be taken in the use of TGF-b-blocking antibodies in autoimmune diseases, as they may unleash powerful pathogenic T cells."	[39]
		**Th1 Downregulated**<br>**Th2 Upregulated in UC**<br><br>• "An **increased IFN-g-producing or T-bet-expressing Th1 cells** (Fig. 3A) and a **decreased IL-13-producing or Gata3+-expressing Th2 cells** (Fig. 3B) **in patients with CD were observed compared with patients with UC**."<br><br>• "Interestingly, the prevalence of IFN-g+ or T-bet+ **Th1 cells were significantly decreased in both patients with UC and CD in comparison with HC (Fig. 3A)."**<br><br>• An **increased IL-13-producing or Gata3-expressing CD4+ Th2** cells were found in patients with **UC**, **but not in patients with CD, when compared with HC (**Fig. 3B). Interestingly, the prevalence of IFN-g+ or T-bet+ **Th1 cells were significantly decreased in both patients with UC and CD in comparison with HC (Fig. 3A).**		[156]

Th2 = ((Th0 **AND** (IL10 **OR** ((IL18 **AND** IL4) **AND NOT** IL12)) **OR** (Th2 **AND** IL4)) **AND NOT** ((Treg **OR** IFNg **OR** TGFb) **AND** Th2)

25	IL4<br><br>Altered [200]	IL18	"In the absence of IL-12, IL-18 drives the Th2 response by inducing IL-4, resulting in increased numbers of IL-4 positive cells from NK T cells [30]."	[16]
		Th2	"Th1 cells are major producers of IFN-g, and Th2 cells are major producers of IL-4, IL-5, and IL-13."	[111]

			"By intracellular FACS analysis, Th2-type cytokines (e.g. IL-4 and IL-13) were hardly detectable in Th1 or Th17 cells (Fig. 4A)."	
			"TH2-cell lineage, producing IL4, IL5 and IL13"<br><br>"Gata3 is to reorganize chromatin structure in the so called TH2-locus (see below), encompassing the IL4, IL5 and IL13 genes**[8]**."<br><br>"Gata3 reorganizes chromatin structure in the Th2 locus, encompassing the Il4, Il5 and Il13 genes, enhancing their transcription competence **(13)**."	[201]
			**[8] Review, mice: "**Mast cells, like Th2 cells, produce large quantities of IL-13<br>and IL-4 (36)."<br><br>"Despite the minor effect on Il-4 expression, acute deletion of Gata3 in differentiated Th2 cells led to pronounced reduction of Il5 and Il13 expression, consistent with the fact that both the Il5 and Il13 promoters contain functional GATA3 binding sites (31, 32)."<br><br>"Th2 cells activate Il4, Il13, and Il5 transcription with similar kinetics, the peak steady-state level of transcripts and the resulting amounts of cytokine protein are dramatically greater in Th2 cells (43–45)"	[202]
			**(13) mice: "**This response is dominated by a Th2 cytokine secretion profile characterized by high levels of IL-4, IL-5, IL-10, and IL-13, and importantly, fails to result in appreciable IL-12 production **(34, 35)**"<br>---<br>**(34, 35):** Mice	[203]
			**Review, mice:** "TH2 cells function both through their production of various TH2 cell-associated cytokines, including interleukin-4 (IL-4), IL-5, IL-9, IL-13 and IL-25"	[204]
			"IL4 is produced by several cell types including mast cells, basophils and activated T lymphocytes **(7,8).**"	[205]
			**8:** "The cytokines IL-4 and IL-13 are produced by T helper type 2 (Tj,2} cells in response to antigen receptor engagement, and by mast cells and basophils upon cross-linkage of the liigh-affinity receptor for immunoglobulin E (lgE) **(/, 2).**"	[206]
			**"**IL-4, a member of the g-chain receptor cytokine family, is considered a crucial mediator of CD4? Th2 T cell differentiation and suppression of IFN-g-producing CD4+ Th1 cells **[1]"**.	[183]
			**[1]** "IL-4 such cells to develop into cells capable of producing IL-4 and a series of other cytokines including IL-5, IL-10 and IL-13 (i.e. TH2-like cells) **(7, 8)**."<br>---<br>**7, 8** → MICE	[184]

		NK (with GLY act)	Secretion: The text do not mention what the picture shows.<br><br>"The above studies of ulcerative colitis provide a basic framework with which to understand the immunopathogenesis of UC; nev**ertheless, they leave certain basic questions unanswered including the identification of the glycolipid antigen or antigens that stimulate UC NKT cells and the mechanism by which IL-13 stimulates NKT cell cytotoxic activity**."	[113]
		AND NOT TGFb	"TGFb- could serve to inhibit IL-4 and IFN- g"<br>(Already inhibits Th2)	[207]
		**Downregulated CD Figure**		[208]
		**Alteration:**<br>Downregulated?<br>Upregulated?<br><br>"The number of **IL-4 secreting cells in the mucosa of CD patients is only a third of that of healthy controls,** and the **amount of IL4 mRNA and secreted protein is also significantly lowered**. Conversely, **significantly increased expression of IL4 mRNA expression is associated with early ileal lesions of CD patients**. A functional polymorphism in the IL4 gene promoter (−34C/T) was associated with CD in a British population [Aithal et al. (2001) Association of single nucleotide polymorphisms in the interleukin-4 gene and interleukin-4 receptor gene with CD in a British population."		[200]
		IL4 = Th2		
26	IL15<br><br>Overexpression in CD and UC<br><br>[209]	MACR AND (LPS OR IFNg)	"A study by **Liu et al,3** involving LPMCs and PBMCs isolated from adult patients, ascertained the potential relevance of IL-15 as a proinflammatory cytokine in the immunopathogenesis of IBD. T**hey reported that IL-15 was highly expressed after lipopolysaccharide** or **TNF-a stimulation** of LPMCs from IBD patients but not from controls."<br><br>**Comment:** LPMCs: Lamina Proprial Mononuclear cells. In the network they are expressed as MACR	[112]
			**Liu et al,3:**<br>FIGURE 2: "IL-15 production by LPMC were incubated with LPS or IFN-g"<br><br>"Our results show local **production of IL-15** in the inflamed mucosa in IBD as evidenced by immunohistochemistry but also by culture of LPMC with **LPS** or **IFN-g**, allowing us to demonstrate the actual secretion of this cytokine."<br>**Figure 2**	[210]

		TNFa	“A study by **Liu et al,3** involving LPMCs and PBMCs isolated from adult patients, ascertained the potential relevance of IL-15 as a proinflammatory cytokine in the immunopathogenesis of IBD. T**hey reported that IL-15 was highly expressed after lipopolysaccharide** or **TNF-a stimulation** of LPMCs from IBD patients but not from controls.”<br><br>Liu et al: IL15 is not produced after TNFa stimulation!!!	[112]
		NFkB	“Interestingly, interleukin-15 (IL-15), a cytokine induced upon NF-κB activation in multiple cell types **8**, is highly upregulated in the epithelium and the lamina propria (Lp) of CD patients9”<br><br>**8:**[211] Do not mention this.	[209]
		MACR	“during periods of immune response and inflammation, low level of IL-15 protein is produced, only by a limited number of cells like **activated macrophages** and epithelial cells.”<br><br>“In terms of transcriptional control, levels of IL-15 mRNA were up-regulated in lipopolysaccharide (LPS)-stimulated macrophage and virus-infected cell lines”	[212]
			“Immunohistochemistry demonstrated local **IL-15 production by macrophage**s in inflamed mucosa from IBD patients”	[210]
		FIBROBLAST<br><br>OR<br><br>MACR	“CD4+CD28- T cells are thought to promote the formation and maintenance of RA inflammatory lesions mainly through IFNg release. IFNG perpetuates synoviocyte pathology, which is associated with secretion of TNFa, **IL-15,** and tissue-injurious metalloproteinases by synovial **fibroblasts and macrophages (33)”**	[65]
			**(33)** “fibroblast-like synoviocytes.”<br><br>**Comment:** We assume that it would be the same for IBD fibroblast.	[213]
			“**IL-15** is expressed preferentially by nonlymphoid tissues, epithelial, and **fibroblast** cell lines and by **activated monocytes/macrophage**”	[214]
**IL15** = (FIBROBLAST **AND** (MDP **OR** LPS **OR** PGN)) **OR** (MACR **AND** (LPS **OR** IFNg))				
27	IL12<br><br>Upregulated in CD [30]	AND NOT (TNFa AND MACR)	“TNF-a is a potent and specific inhibitor of IL-12 p40 and p70 secretion from human macrophages”<br>“TNF-a inhibits IL-12 p40 production in human MDM independently of IL-10”<br><br>“In this report, we observed that **TNF-a is a potent and selective inhibitor of IL-12 p40 and p70 production from human MDM** (Fig.1). This inhibition is observed when **MDM were pre-exposed to TNF-a before, but not at, the time of stimulation with LPS** or S.aureus (Fig. 2). T**he fact that the inhibitory effects of TNF-a macrophages take a considerable preincubation period (16 h)** to	[215]

			attain suggests that TNF-a may be inducing de novo protein synthesis required for its inhibitory activities."<br><br>MDM: monocytes derived macrophages	
			"**The basis for decreased IL-12 production in vivo is clearly multifactorial,** involving both loss of CD11chigh DCs as well as alterations in the responsiveness of macrophages and remaining splenic DCs. **There is no demonstrable mechanistic role for** B or T lymphocytes, **the soluble mediators IL-10, TNF-α**, IFN-α/β, nitric oxide, or the NFκB family members p50, p52, or RelB140"<br><br>**<u>Comment</u>**: is multifactorial. No demonstrable mechanistic role for TNFa. Ma et al. 2000 uses MDM cells which il12 expression was inhibited by TNFa when previous exposure.	[216]
		(MACR OR DC) AND (LPS AND IFNg)	"Goriely et al. showed that lipopolysaccharide (LPS)- and IFN-γ-induced human Il12a gene activation was immediately"<br><br>"An important pathway in robust **IL-12 induction is the requirement for "priming" of LPS-activated macrophages and DCs by IFN-γ** for the expression of maximal amounts of Il12a and Il12b mRNAs and for IL-12 production4,20,38"	
		MACR AND PGN	"**Wild-type macrophages** showed **dose-dependent production of IL-6**, TNFa and IL-12 **in response to** MALP-2 and **PGN**."	[37]
		AND NOT IL10	"For instance, enhanced IL-10 production by TLR2-primed DCs abolishes TLR3 / 4-mediated production of proinflammatory IP-10 and IL-12p35, 25 "	[14]
			"IL-10 is one of the most potent inhibitors of macrophage activation including the suppression of IL-12 production (24)."	[215]
			**"IL-10** is a cytokine with potent anti-inflammatory activity that has been demonstrated to be able to **inhibit the synthesis of** several cytokines (24), including IP-10 (25) and **IL-12** (26)"	[217]
			**<u>Comment</u>**: Not need to add: AND NOT IL10 because it inhibits MACR and DC	
		DC	"In cells from control tissue, the proportion of IL-12–producing DCs was variable and low"<br>"In cells from the healthy colonic mucosa, there were no IL-6 –positive DCs and few, if any, IL-12–positive DCs"<br>"In contrast, **DCs making IL-12** and IL-6 were **prominent in cells from active Crohn's disease tissue.**"	[38]
			"In addition, **NFκB activation of APCs** (including **dendritic cells)** by microbial adjuvants induces the expression of MHC class II antigens, co-stimulatory molecules, **IL-12** and IL-23, which can activate TH1 and TH17 cells, respectively, if the appropriate antigen is present.<br>"In vivo studies show that commensal bacteria selectively activate	[30]

			IL-12 p40 in dendritic cells of the distal ileum.47”	
		(DC OR MACR) AND TLR2 AND NFkB	Figure2: “In antigen-presenting cells, TLR2-NFkB signaling may be altered by CARD15 mutations, leading to exaggerated immune responses through proinflammatory cytokine production (such as IL-12 or interferon- g.”	[14]
			“In addition, **NFκB activation of APCs** (including **dendritic cells)** by microbial adjuvants induces the expression of MHC class II antigens, co-stimulatory molecules, **IL-12** and IL-23, which can activate TH1 and TH17 cells, respectively, if the appropriate antigen is present.”	[30]
		AND NOT TNFa OR (IL10 OR TGFb)<br><br>OR IFNg<br><br>OR (IL4 OR IL13) AND MACR	41→ MICE<br>[46] → “Ma and coworkers demonstrated that **in human monocyte-derived macrophages TNF-α inhibits both LPS-induced and IFN-γ-enhanced IL-12 production at the level of transcription of the p40 gene**, but not the p35 gene whose mRNA expression is not significantly suppressed”<br>IL12 = AND NOT TNFa 41,46<br><br>IL12 = IFNg 30<br><br>IL12 = (IL4 OR IL13) AND MACR 57<br><br>IL12 = AND NOT (IL10 OR TGFb) 35, 17	[72]
		(MACR AND ((LPS OR PGN) AND IFNg)) AND NOT TNFa	41→ MICE<br>[46]:<br>“To investigate the relationship between on LPS-and IL-12 production, we analyzed the effects of exposure of human monocyte-derived macrophages to TNF-a on LPS or SAureus induced IL12 production in the presence or absence of IFNg. “<br><br>“**TNFa is a potent inhibitor of IL12 p40 and p70 section from human macr** induced by LPS or S.aureus.”<br><br>“Fig. 1A shows that **pre-exposure of MDM to TNF-a inhibited the production of IL-12 p40 and p70 induced by IFN-g** and S. aureus in a dose-dependent manner.”<br><br>“These data demonstrate **a selective negative regulation on IL12 by TNFa, identifying a direct negative feedback mechanism for inflammation-induced suppression of IL12 gene expression**.”<br><br>“We show here that i**n human monocyte-derived macrophages (MDM)**,3 **selectively inhibits IL-12 p40 transcriptio**n, but not p35, identifying a novel regulatory mechanism of action for TNF-a - induced suppression of IL-12 production.”<br><br>“In this report, **we observed that TNF-a is a potent and selective TNF-a inhibitor of IL-12 p40 and p70 production from human**	[124]

			**MDM**(Fig.1). This inhibition is observed **when MDM were pre-exposed to TNF-a before, but not at, the time of stimulation with LPS or S. aureus**”<br><br>“**FIGURE 3**. **TNF-a inhibits IL-12 p40 production induced by** S. aureus alone **or** by IFN-g and S. aureus.”<br><br>---<br>**IL10:** “IL10 is not responsible for the TNFa mediated inhibition of IL12.”<br><br>---<br>**IL6:** “Although IL-6 has been reported to inhibit IL-12 secretion (22), our data showed a selective effect by TNF-A in suppressing IL-12 p40 secretion.”<br><br>---<br>**NFkB:** “TNF-a treatment does not alter the nuclear translocation and/or DNA binding of the major transcription factors involved in IL-12 p40 gene promoter activation”<br><br>“**Fig. 7** shows that pre-exposure of MDM to TNF-a alone for 40 h induced marginal NF- kB binding to the kB “half site” element at -107/-117 (TGAAATTCCCC) of the IL-12 p40 promoter (26), whereas stimulation with LPS and IFN-g caused a strong increase in the binding IFN-g activity to this element, which was not inhibited by the pretreat-ment with TNF-a”<br><br>“These results suggest that **the inhibitory effects of TNF-a on IL-12 gene expression are not mediated through NF- kB**.”<br><br>---<br><br>**<u>comment:</u>**<br>• S Auruеus = PGN<br><br>**IL23 shares p40 subunit,** then TNFa inhibits also IL23 production, although:<br>“**”TNFa-mediated inhibition is selective for IL-12 p40 gene expression”**	
			“There are also effective mechanisms that down-regulate IL-12 production and the responsiveness of T and NK cells to IL-12, such as those mediated by cytokines IL-10 and (5).”	[124]
		IL12 = AND NOT (IL10 OR TGFb)	5<br>“IFN-3' and GM-CSF are among the stimulatory cytokines, enhancing IL-12 production and other functions of monocytes/macrophages (12, 13), whereas **IL-10, IL-4, and TGFb** are usually considered **as the most important deactivating factor**s (14).”	[218]
			**Figure 3 :**<br>“In contrast to **IL-10 and TGFb**, which **consistently inhibited cytokine production induced by either LPS or S. aureus**, IL-4 and IL-13 often failed to inhibit cytokine production in response to S.	[218]

			aureus and were always less potent in inhibiting"<br>Fifure 4:	
		IL12 = (IL4 OR IL13) AND (MACR OR DC) AND (LPS OR PGN)	Figure 2→ "we show that **pretreatment of PBMC with IL-4 or IL-13** for/>20 h **enhances several fold their ability to produce IL-12** and TNF-ot in response to **Staphylococcus aureus or LPS.**"<br><br>"The data presented here show that treatment of PBMC with IL-4 or IL-13 for />20 h primes them for production of IL12 and TNF-cz in response to S. aureus and LPS, whereas their ability to produce IL-1B or IL-10 in response to LPS"<br><br>"In the **absence of a cytokine-inducing stimulus such as S. aureus or LPS**, **IL-4 did not have a direct stimulatory effect on the mRNA accumulation or production of either IL-12 or TNF-a.**"<br><br>**"The ability of IL-4 and IL-13 to prime PBMC for IL.-12 production is particularly surprising** because **IL-12 and IL-4 are known to play an antagonistic role in inducing development of Thl and Th2** immune response, respectively (47). **The equilibrium between IL-12 and IL-4 early during an im-mune response most likely determines whether the response is Thl or Th**2 (48)."<br><br>"The **priming effect of IL-4 and IL-13 on IL-12 production by PBMC is almost completely suppressed by IL-IO**, another cytokine prevalently produced by Th2 cells, suggesting that the priming effect of IL-4 on II.-12 production is not effective when a Th2 response predominates."<br><br>**<u>Comment:</u>**<br>"PBMCs include lymphocytes (T cells, B cells, and NK cells), monocytes, and dendritic cells."	[218]
			IL12 = DC AND LPS<br>**figure 2.**	[219]
		DC AND IL1b	"Here, **IL-1β is identified as a new IL-12-inducing agent,** acting conjointly with CD40 ligand (CD40L) **on human monocyte-derived DC in vitro.** The effects of IL-1β were dose dependent, specifically blocked by neutralizing antibodies, and were observed both in immature and mature DC. Immature DC secreted more IL-12 than mature DC, but the effects of IL-1β were not due to a block of DC maturation as determined by analysis of DC surface markers."<br><br>"The mechanisms of action of IL-1β could be contrasted to that of other inducers of IL-12 such as IFN-γ and lipopolysaccharide (LPS)."<br>**figure 1.**	[220]
		IL12 = LPS AND NFKB AND MACR AND IFNg	"We and others have shown previously that **NF-KB is a critical factor involved in the induction of IL-12 p40 transcription by LPS and IFN-G** (26, 27). Fig. 7 shows that preexposure of MDM to TNFA alone for 40 h induced marginal NF- KB binding to the "half site" element at	[124]

			?107/?117 (TGAAATTCCCC) of the IL-12 p40 promoter (26), whereas stimulation with LPS and IFN-G caused a strong increase in the binding”	
			27 “A NF-kB element was found to be functionally important for promoter activation in response to LPS and IFN- g (17).”	[221]
			26,<br>“**We demonstrate binding of this sequence to NF-?B** (p50/p65 and p50/c-Rel) complexes in macrophages activated by several p40-inducing pathogens **and provide functional data to support a role for NF-kB family members in IL-12 p40 activation.** Finally, we find that **IFN-g treatment of cells enhances this binding interaction, thus potentially providing a mechanism for IFN-? augmentation of IL-12 production by macrophages.**” → MURINE	[222]
		IL18	**“IL-12 production is controlled by** a variety of cytokines including interferon **(IFN)-g,** **IL-18,** granulocyte–macrophage colony-stimulating factor (GM-CSF), **IL-4,** and even **IL-12 itself**, partly explained by upregulation of the IL-12R by these cytokines. Optimal induction of IL-12 very often requires a combination of microbial, CD40, and cytokine stimuli (17, 26).”<br><br>“Moreover, **IL-23 stimulation could drive IL-12 and IFN-g production at levels** comparable to stimulation with IL-12 (43).”	[75]
			17 → Do not mention IL18	[223]
			26→ Do not mention IL18	[224]
			43→ “IL-23 and IL-12 have overlapping, but distinct, effects on **murine dendritic cells**”	[225]

Comment: the BF can be reduced but the results would be the same. It is not reduced just in case a polymorphism in one of the nodes is implemented

---

Comment:
“This receptor family includes **receptors for IL-12 and IL-23 which are both heterodimeric cytokines consisting of two protein subunits, namely p35/p40 and p19/p40 subunits**, respectively, hence sharing the p40 subunit[71].

- **IL-12 binds to the IL-12R** which is composed of **IL-12Rβ1** and IL-12Rβ2 subunits,
- **whereas IL-23 binds to the IL-23R** complex, composed of IL-23R and **IL- 12Rβ1**[72]”
-

“The **IL-23R is expressed on T-cells,** but has also been found to be expressed by **NK cells**[72,77]”

“The **binding of ustekinumab to free IL-12 and IL-23 cytokines block their interaction with IL-12Rβ1[89]** expressed on **T-cells, NK cells, macrophages, DCs, and B-cells**.” [70]

IL12 = ((((MACR OR DC) AND (LPS OR PGN) AND IFNg) AND NOT TNFa) OR (DC AND IL1b) OR (IL12 AND (IL13 OR IL4))) AND NOT (IL10 OR TGFb)f

	IL5<br><br>(eliminada, no hace nada en la red)	Th2	Secretion	[109]
			TH2-cell lineage, producing IL4, IL5 and IL13	[201]
			TH2 cells function both through their production of various TH2 cell-associated cytokines, including interleukin-4 (IL-4), IL-5, IL-9, IL-13 and IL-25	[204]
			IL5 = Th2	
28	IL13<br><br>Upregulated in UC<br><br>[30]		**“IL-13 is known as an effector cytokine that drives inflammation in ulcerative colitis**. This role has been shown by using a suit of murine models of ulcerative colitis from confirmed excess production of IL-13 in human disease [145].”<br><br>“IL-13 level in patients with active ulcerative colitis is higher which is resulted **from lamina propria mononuclear cells**. They also have shown increased epithelial pSTAT6 in comparison to the active Crohn’s patients and healthy controls [148, 149]”.<br><br>“The deleterious effects of IL-13 on the epithelial barrier and colonic epithelial cells occur when the production of IL-13 in ulcerative colitis is extremely High. **IL-13 has been reported to activate the proapoptotic molecule cysteine-aspartic acid protease 3 (CASP3)** in mouse colonic epithelial cells as well as in patients with active ulcerative colitis [150].”<br><br>“In vitro studies show that **IL-13 can induce epithelial cell apoptosis and it also causes disruption in tight junction by inducing claudin 2 [151]**. These observations support the idea that IL-13 is a potentially valuable target for therapeutic objects in ulcerative colitis.”	[226]
		Th2	“Th1 cells are major producers of IFN-g, and Th2 cells are major producers of IL-4, IL-5, and IL-13.”<br>“By intracellular FACS analysis, Th2-type cytokines (e.g. IL-4 and IL-13) were hardly detectable in Th1 or Th17 cells (Fig. 4A).”	[111]
			“TH2-cell lineage, producing IL4, IL5 and IL13”<br><br>“Gata3 is to reorganize chromatin structure in the so called TH2-locus (see below), encompassing the IL4, IL5 and IL13 genes[8].”<br><br>“Gata3 reorganizes chromatin structure in the Th2 locus, encompassing the Il4, Il5 and Il13 genes, enhancing their transcription competence (13).”	[201]
			**[8] Review, mice:** “Mast cells, like Th2 cells, produce large quantities of IL-13 and IL-4 (36).”<br><br>“Despite the minor effect on Il-4 expression, acute deletion of Gata3 in differentiated Th2 cells led to pronounced reduction of Il5 and Il13 expression, consistent with the fact that both the Il5 and Il13	[202]

			promoters contain functional GATA3 binding sites (31, 32).”<br><br>“Th2 cells activate Il4, Il13, and Il5 transcription with similar kinetics, the peak steady-state level of transcripts and the resulting amounts of cytokine protein are dramatically greater in Th2 cells (43–45)”	
			**(13) mice: “**This response is dominated by a Th2 cytokine secretion profile characterized by high levels of IL-4, IL-5, IL-10, and IL-13, and importantly, fails to result in appreciable IL-12 production **(34, 35)**”<br><br>**(34, 35):** Mice	[203]
			**Review, mice:** “TH2 cells function both through their production of various TH2 cell-associated cytokines, including interleukin-4 (IL-4), IL-5, IL-9, IL-13 and IL-25”	[204]
			“Interleukin-13 (IL-13) is a cytokine secreted by activated T-helper 2 (Th2) cells. It is a counter-regulatory system for the type I immune response and has emerged as an important regulator of type II cytokine-mediated immune responses **[1]**.”<br><br>“...Several years later, three groups cloned the complementary DNA (cDNA) for human IL-13 **[5–7]**.”<br><br>“This effect has been proved by investigating related responses on both human and mouse cells which suggests a homeostatic role of IL-13 in the gut **[142]**.”	[226]
			**[1] Review (old):** Th2 cells secrete the cytokines IL-4, IL-5, IL-10 and IL-13, which effect humoral immunity to helminthic parasites and are responsible for immune responses to persistent antigens, for instance allergens 1, 2 and 3”	[227]
			**5** “Here we report the discovery by molecular cloning of a new interleukin (interleukin-13 or IL-13) expressed in activated human T lymphocytes. Recombinant IL-13 protein inhibits inflammatory cytokine production induced by lipopolysaccharide in human peripheral blood monocytes.”	[228]
			**[142] mice:** “we extend our in vitro findings and determine that IL-13 increases IL-10 production from Th17-polarized cells and that IL-13–induced IL-10 production negatively regulates the secretion of IL-17A and IL-21.”<br>“We have shown previously that IL-13 negatively regulates IL-17A production from Th17 cells in both mice and humans **(13, 14)”**	[229]
			**14:** “Conclusions—IL-13Rα1 is expressed on human CD4+ Th17 cells, and IL-13 attenuates IL-17A production at polarization and restimulation. While IL-13 is an attractive therapeutic target for decreasing symptoms associated with asthma, these results suggest that therapies inhibiting IL-13 production could have adverse side effects by increasing IL-17A production.”	[146]

		"inflammation of ulcerative colitis (UC) has recently been shown to be associated with excess production of IL-13 **[1].**"		[230]
		IL13 = Th2		
29	Treg<br><br>Downregulated in Blood, Upregulated in inflamed IBD mucosa<br><br>CD and UC<br><br>[231]	TGFb	"since Th17 cells usually require **TGF-β** for differentiation, **a cytokine also involved in regulatory T cell differentiation**"<br><br>**<u>Comment:</u>** Th17 do not need TGFb for differentiation.	[113]
			MICE: "Transforming growth factor- b (TGF-b) is a critical differentiation factor for the generation of Treg cells 7".	[207]
			"We fortuitously identified **TGF-β** and IL-6 as the differentiation factors for Th17 cells while looking for the factors that inhibited the **TGF-β-driven conversion of naive Tcells into Foxp3+ Tregs** in vitro and in vivo"	[15]
			"TGF-b1 promotes Treg differentiation, which in turn suppresses adaptive T-cell responses and prevent autoimmunity **[11, 12].**"	[162]
			"**TGF-β** has been extensively characterized as being required to maintain immunological tolerance, **acting in both differentiation and maintenance of Foxp3+ Tregs** that restrain effector T cell responses [33]."	[164]
		TNFa	"Previous work has suggested that the subset of CD4?CD25hi cells is enriched in Tregs,24 and, therefore, this population was isolated."<br><br>"As Tregs appeared to express TNFRII, we next determined whether signaling through TNFRII influenced the regulatory function of CD4+CD25hi Tregs. **The addition of soluble TNF** to the culture of CD4+CD25hi responders and CD4?CD25hi **Tregs completely reversed the suppression of the proliferation of CD4?CD25? T cells without influencing the anergic phenotype of CD4?CD25hi Tregs** (Figure 3A)..."	[151]
		AND NOT (Th17 OR IL22)	"it is not surprising that Th17 cells and regulatory T cells have a "ying-yang" relationship wherein the development of one type of cell is reciprocal to the development of the other type of cell. This is seen in the fact **that induction of Foxp3 expression,** the signature protein of regulatory T cells, **inhibits RORγt function, the main IL-17 transcription factor. Similarly, the induction of Th17 cells inhibits Foxp3 expression 25,26.**"	[164]
			**25** "We have found that **AhR cooperates with RORγt** to induce **maximal amounts of IL-17 and IL-22 and also inhibits TGF-β-induced Foxp3 expression**, thus **highlighting the antagonism between Th17 and Treg cell differentiation** (L. Zhou and D. R. Littman, unpublished)"<br><br>AhR: aryl hydrocarbon receptor (the regulation of transcription of	[164]

			Th17 cytokines)	
			**26** “Figure 5”	[232]
		AND NOT (IL6 OR IL21)	**MICE:** “**IL-6**, an acute phase protein induced during inflammation 8,9, **completely inhibits the generation of Foxp3+ Treg cells induced by TGF-b**”	[207]
			“in contrast, **IL-6 inhibits TGF-b- induced Treg differentiation**.”<br><br>“**IL-6 induces the generation of Th17 cells from naıve T cells together with TGF-b and inhibits TGF-b-induced Treg (iTreg) differentiation** [13–15].”<br><br>(**Comment:** EMAIL: rene.de.waal.malefyt@merck.com assure that TGFb do not induce Th17 differentiation. Reference is not trustful)	[162]
			“Recent studies have demonstrated that IL-6 has a very important role in regulating the balance between IL-17 producing Th17 cells and Treg. **IL-6 (plus TGFb) induces the development of Th17 cells from na¨ıve T cells; in contrast, IL-6 inhibits differentiation into Treg** [34].”	[142]
			“Induction of FOXP3 was **inhibited by IL-6** and to a greater extent **by IL-21**—transcription factors that induce RORC2”.<br>**Figure 2: IL-6 and IL-21** inhibit FOXP3	[82]
		AND NOT IL23	MICE: “These findings strongly suggest that **IL-23 suppresses regulatory T cell developmen**t and thus introduce the possibility that mice that lack IL-23 fail to develop colitis, not because they cannot produce a key effector cytokine (IL-17) but rather because they have a dominant regulatory T cell response.”	[113]
		TLR2	**TLR2** also **activates regulatory T cells** via PI3K to secrete transforming growth factor- Β **27**, which is essential for tissue healing and epithelial restitution in the intestine.	[14]
			**27:** The finding that **HSP60-treated Tregs** significantly u**pregulated their secretion of TGF-β and IL-10** (Figure 7A) suggests that costimulatory signals, such as HSP60, might account for the importance of these cytokines detected in Treg-mediated suppression in vivo (13–15).	[85]
		Treg = CD4+CD25highFoxP3+		[141]
		“Although there are **many types of Treg**, naturally occurring Treg (nTreg) and iTreg are the best characterized:<br>● **nTreg** are generated in the thymus and acquire the expression of the transcription factor Foxp3.		[162]

			• **iTreg** are generated from naı¨ve T cells in the periphery or in vitro, after stimulation with antigens and TGF-b [58]	
			**<u>Downregulated in Blood, Upregulated in inflamed IBD mucosa. But do not present a compromised function</u>**<br><br>“In peripheral blood, CD4?CD25high T cells from IBD patients retain their suppressive activity. **CD4?CD25high and FOXP3?Treg cells are increased during remission but decreased during active disease**.”<br><br>“**Different than peripheral blood, inflamed IBD mucosa contains an increased num-ber of CD4?CD25high T cells, FOXP3? T cells, and tran-scripts for FOXP3 compared with noninflamed mucosa. However, the increase of FOXP3? T cells in IBD lesions is significantly lower compared with inflammatory controls.**”<br>• Regulatory T cells in peripheral blood from IBD patients and controls.<br>Figure 2<br><br>• CD4? CD25high T cells are increased in IBD lesions.<br>Figure 4<br><br>“Because our data demonstrate that IBD is character-ized by an inadequately low number of CD4?CD25? Treg cells, it is interesting to note that, to date, there is no indication to suggest that CD4?CD25? Treg cells from IBD patients might have a compromised suppressor function.”	[231]
			**<u>Downregulated:</u>**<br><br>“We showed that **CD4?CD25high T-cell frequency was diminished in patients with naı¨ve CD.** Compared to CD, the **CD4?CD25high T-cell frequency decrease was stronger in UC**.”<br><br>“**In CD, the percentage of CD4?CD25high T cells was not correlated to CD activity, and did not differ between patients having a CDAI superior or equal to 150** (2.29 ± 0.22%) and those with a CDAI inferior to 150 (2.41 ± 0.27%) (Table 3). CD4?CD25high T-cell frequency was not significantly influenced by the site of disease (terminal ileum and/or upper gastrointestinal [L1, L4] versus colon [L2] or ileocolon [L3]), disease behavior”<br>“However, **CD4?CD25high T cell frequency was significantly lower in the group of patients who had no treatment for 6 months** or who were recently diagnosed (naı¨ve CD)”<br>**Figure 3**	[233]
**Treg** = (THR_Th0 **AND** (TGFb **OR** TLR2)) **AND NOT** ((IL6 **OR** IL21 **OR** IL23 **OR** Th17 **OR** IL22 **OR** TNFa) **AND** Treg)				
30	NK<br><br>Upregulated in CD and UC<br>[1]	(NKT AND IL13)	“..in addition, IL-13 enhances **NKT** cell cytotoxity.”	[113]
		(DC AND IL15)<br><br>OR IL2 OR	“It has also been shown that **IL-15** stimulates the functional **maturation of DCs** and **IL-15 enhances the ability of DCs to activate** CD8+ cells and **NK** cells by up-regulating the expression of costimulatory molecules and IFN-c **[37,38]**”	[212]

		(NK AND IL12<br><br>(NK BRIGHT)	“IL-15R complex is expressed on T cells, B cells, natural killer (NK) cells, monocytes, macrophages, fibroblasts and dendritic cells (DCs) [25]”	
			**[38]:** “IL-15 is able to activate innate mediators of immunity such as NK cells and macrophages **(14, 24)**.”	[234]
			**[37]:** IL**-15 also modulates the functional maturation of dendritic cells and contributes to the survival and activation of** neutrophils, B and **NK cells (23,24,25)**	[235]
			**23 “**We therefore proceeded to compare the proliferative effects of IL-15 and IL-2 on highly purified NK cells that constitutively express the IL-2R. CD56b~ ht NK cells constitutively express a high affinity IL-2R and exhibit a proliferative response to concentrafions of IL-2 that partially or fully saturate this receptor”<br><br>“In contrast to IL-2, **IL-15** did not induce proliferation of CD56 bright NK cells at concentrations ranging from 0.01 to 1.0 ng/ml in over 20 separate experiments. However, at 10 ng/ml, IL-15 produced a maximal proliferative response in CD56b~g ht NK ceils.”<br><br>“IL15 or IL.2 synergize with IL-12 to enhance the cytotc~ic activity of CD56 ~NK cells.”	[78]
			**“**We evaluated IVIG modulation of human blood lymphocyte response to IL-2 and other cytokines. Neither IVIG nor low concentrations of IL-2 (3–30 U/ml) induced lymphocyte proliferation, but in combination they synergistically enhanced proliferation of NK cells. The CD56bright cells expanded more than CD56dim NK cells, with 90% of NK cells dividing up to 8 generations by day 6,while b8% of T cells divided. IVIG also potentiated NK cell proliferation with IL-12, IL-15 and IL-18. The IVIG+cytokine-expanded NK cells were less cytotoxic for K562 cells, than NK cells with cytokine alone.”	[236]
			“**NK cell activation and cytolytic function is regulate**d by many mechanisms, including expression of activating or inhibitory receptors on the surface of NK cells, **cytokine signals, such as IL-2 or IL-15,** and transcription factors associated with NK cell maturation and effector function **(Waldmann et al., 2006)**.”	[237]
			**(Waldmann et al., 2006).**<br>“IL-2 and IL-15 also stimulate the generation proliferation and activation of NK cells **16**.”	[238]
			**16 “Mice** that lack the IL-2 gene have NK cells, whereas mice and humans that lack IL-2Rgc do not have NK cells. Further, treatment of mice with an antibody directed against IL-2Rb results in a loss of the NK cell compartment. **These data suggest that a cytokine other than IL-2, which binds to IL-2Rbgc, is important for NK cell**	[239]

			**development and survival in vivo**. “	
		IL10 AND IL18	Furthermore, **IL-10 can also co-stimulate NK cell** proliferation and cytokine production [22].	[176]
			**22 MICE:** “Our data showed showed that **IL-10 or IL-18 alone could elevate NK cell cytotoxic activity** (Fig. 2A). How- ever, t**he combination of IL-10 and IL-18 resulted in a greater than fourfold increase in NK** cell cytotoxicity”	[240]
		IL21	“**IL-21** has emerged to be **a strong driver of NK cell proliferation and expression of effector molecules in NK cells**, while being a poorer driver of T-cell expansion.“ **12**	[58]
			**12 review:** **“**IL-7 is also a potent vaccine adjuvant and it increases the effectiveness of adoptive transfer therapies, properties that it shares with IL-15, IL-21 and, in some cases, IL-2 **(REF. 108).**” d**o not mention NK**	[241]
			**(REF. 108) → Do not!**<br>“L-7, IL-15, and IL-21 each possess properties that can be exploited in the context of immunotherapy for cancer. Each has been demonstrated to mediate potent vaccine adjuvant effects in tumor models, and each can enhance the effectiveness of adoptive immunotherapies. Although the overlap among the agents is significant, IL-7 is uniquely immune restorative and preferentially augments reactivity of naive populations, IL-15 potently augments reactivity of CD8 memory cells and natural killer cells, and I**L-21 preferentially expands the inflammatory Th17 subset and may limit terminal differentiation of effector CD8 cells**.”	[242]
		AND NOT Treg	**“nTreg and iTreg** are immunosuppressive and **could efficiently suppress the function of** CD4, CD8, and **NK cells.”**	[58]
		IL2	IL-2 and IL-15 are known to have overlapping roles in inducing NK cell activation and cytolytic activity as well as in maintaining NK cell homeostasis and survival. Recent data from us and others have shown that both IL-2 and IL-15 efficiently induce proliferation of NK cells [8,9].	[58]
			“Here, we demonstrate that **CD56bright NK** cells are present in human lymph nodes and that endogenous T cell–derived IL-2, acting through the NKhigh-affinity IL-2 receptor, **costimulates CD56bright NK** cells to secrete **IFN-g”**<br><br>“The majority (approximately 90%) of **human NK** cells are CD56dim and express high levels of Fc?RIII (CD16), whereas a **minority (approximately 10%) are CD56bright** and CD16dim/neg”	[243]
			Figures a, b, c and d	[78]

			“**NK cell activation and cytolytic function is regulate**d by many mechanisms, including expression of activating or inhibitory receptors on the surface of NK cells, **cytokine signals, such as IL-2 or IL-15,** and transcription factors associated with NK cell maturation and effector function **(Waldmann et al., 2006).**”	[237]
			**(Waldmann et al., 2006).**<br>“IL-2 and IL-15 also stimulate the generation proliferation and activation of NK cells **16**.”	[238]
			**16** “**Mice** that lack the IL-2 gene have NK cells, whereas mice and humans that lack IL-2Rgc do not have NK cells. Further, treatment of mice with an antibody directed against IL-2Rb results in a loss of the NK cell compartment. **These data suggest that a cytokine other than IL-2, which binds to IL-2Rbgc, is important for NK cell development and survival in vivo**. “	[239]
		IFNg OR IL15 OR IL18	“It has been suggested that **NK cells in gut, like T cells, require** priming for activation, a process that involves cytokines such as IFN-γ, IL-15, and IL- 18 **[13]”**	[1]
			**[13]** → do not mention that IL18	[3]
		IL23	“purified NK cells (2.0?105 /ml) from PB of IBD patients and healthy controls were cultured…”<br><br>**“IL-23** potently **induces IBD NK cell differentiation**, leading to secretion of proinflammatory cytokines such as TNF and IFNg” **Figure A**<br><br>“IL-23-activated IBD NK cells exhibited stronger cytotoxicity against the target cells.”<br><br>“IL-23 signaling plays an important role in the maintenance of NK cell maturation and cytotoxicity potential [37]. **Figure**	[69]
			**“Human IL-23R is expressed by both T cells and NK** cells, including NKL cells (Fig. 5, a and b), **consistent with the ability of these cells to respond to IL-23.** We demonstrate here that **IL-23 enhanced the production of IFN-? by NKL** cells. The combination of IL-2, IL-18, and IL-23 induced levels of IFN-g that were substantially greater than those induced by either cytokine alone or when combined solely with IL-2”	[110]
		IL12	“The IL-12R is primarily expressed by **activated T-cells** and **natural killer (NK)** cells, but has been found to be expressed on **dendritic cells (DCs)** and B-cells as well[73,74]”	[70]

			73 →Figure 2<br>"The cytokine is primarily produced by phagocytic and dendritic cells in response to microbial stimulation2 and drives cell-mediated immunity by activating natural killer cells, inducin lymphokine-activated killer cells, and triggering IFN-γ production and T-cell proliferation by TH1 cells **4**."	[244]
			**74** → "IL-12 was identified as a product of Epstein–Barr virus (EBV)-transformed human B-cell lines that can acti-vate NK cells, generate lymphokine-activated killer cells (LAKs), and induce IFN-γ production and T-cell proliferation 9,12"<br><br>"IL-12R is undetectable on most resting T cells, but it is expressed at a low level by NK cells, which probably explains the abil-ity of these cells, and possibly of certain subsets of T cell, to respond rapidly to IL-12."<br><br>"**IL-12 does not induce proliferation of resting peripheral-blood T cells or NK cells**, although it augments proliferation induced by mitogenic lectins, alloantigens, CD3-specific antibodies diesters 9,69, **and it has a direct proliferative effect on pre-activated T cells and NK cells** 12,69"<br><br>"IL-12 induces T cells and NK cells to produce several cytokines — for example, granulocyte–macrophage colony-stimulatory factor and TNF — and it is particularly efficient at inducing the production of IFN-γ9,70,72"	[245]
			"IL-12 ..., and **it principally activates natural killer (NK)** cells and induces the differentiation of naïve CD4+ T lymphocytes to become interferon-gamma (IFN-γ)-producing T helper 1 (Th1) effectors in cell-mediated immune responses to intracellular pathogens **2**"	[216]
			**2 → Not clear**	[246]
			"**IL-12 is produced early in responses to infection** (15) and has **powerful effects on NK cell function** and T-cell development (8)."<br><br>"Along with the important effect that macrophage/DC-derived IL-12 has on T-cell development (15) and NK function (32), IL-12 can also feed back to regulate APC function."	[75]
			"**IL15 or IL.2 synergize with IL-12 to enhance the cytotc~ic activity of CD56 ~NK cells**."<br><br>"**(D) IL15 or IL.2 synergize with IL-12 to enhance the cytotc~ic activity of CD56 ~ NK cells**. CD56 am NK cells were incubated in 1 ng/ml IL-2, 1 rig/m1 LL-15, 10 U/ml ID12, alone or in combination, for 24 h and then tested for cytotoxicity against COLO 205 target cells at an E/T ratio of 5:1. Results represent the mean + SE of triplicate wells."	[78]

			"A number of studies have shown that activation of human NK cells with IL-2, IL-12, or TNF-a can result in cytokine production by human NK cells (16-18)."	
			**ntravenous IgG (IVIG) therapy:**<br><br>**"**We evaluated IVIG modulation of human blood lymphocyte response to IL-2 and other cytokines. Neither IVIG nor low concentrations of IL-2 (3–30 U/ml) induced lymphocyte proliferation, but in combination they synergistically enhanced proliferation of NK cells. The CD56bright cells expanded more than CD56dim NK cells, with 90% of NK cells dividing up to 8 generations by day 6,while b8% of T cells divided. I**VIG also potentiated NK cell proliferation with IL-12, IL-15 and IL-18. The IVIG+cytokine-expanded NK cells were less cytotoxic for K562 cells, than NK cells with cytokine alone."**<br><br>"As shown in the representative histograms in **Fig. 8,** a**fter 6 days of culture, CD56 NK cells were the predominant proliferating cells in response to the combination of IVIG with IL-12, IL-18 or IL-15,** with very low or no response to IVIG, IL-12, IL-18 or IL-15 alone used at the same concentrations."	[236]
		IL23	**"The activity of IL-23** has primarily been described in the context of TH17 cells. However, activity on other cell types, such as CD8+ T cells **and natural killer primarily been described in the context of TH17 cells.** However, activity on other cell types, such as CD8+ T cells and natural killer T cells has been recently reported 29,30."	[244]
			**"IL-23 contributes to the functional response o**f several effector cell sub-types other than CD4+ T cells, including CD8+ T cells **[26**, **natural killer (NK)**, NK T [27, γδ T cells [28], and innate lymphoid cells [29].	[40]
		**killing activity is increased 20-100-fold when NK** cells are exposed to IFN-a and IFN-b, **or to IL-12,** which is one of the cytokines produced early in many infections by dendritic cells and macrophages		[247]
		**Comments:**<br>"NK cells have been found to be increased in inflamed mucosa of IBD patients, and NK cell differentiation is also accelerated in the lamina propria, suggesting that NK cells are involved in the disease pathophysiology."<br><br>"**CD16+ NK cells are found to be increased in the lamina propria from both CD and UC patients** compared with healthy controls, and azathioprine preferentially inhibits proliferation of CD16+ NK cells and induces apoptosis in resting but not in preactivated NK cells **[23]**, indicating that NK cells with cytolytic potential are enriched in the colonic lamina propria of IBD patients and that azathioprine is associated with a reduction in these cells and a normalization of NK cell population		[1]

		in gut mucosa."<br><br>"However, **previous work** has reported that the populations of **CD161+ NK cells are significantly decreased** in the inflamed mucosa of **UC,** whereas the frequency of conventional CD161+ cells is similar among IBD patient and healthy controls" **¿?**	
		NK = (IL15 OR IL2 OR IL12 OR IL23 OR (IL18 AND IL10)) AND NOT (Treg AND NK)	
31	DEF<br><br>Downregulated UC and CD<br><br>[248][8]	DEF are secreted by Paneth cells:<br><br>Mutations that alter Ag elim (AMPs):<br>● ATG16L1 (altered granule exocytosis XBP)<br>● XBP1 (altered Paneth cell morphology)<br>● KCNN4 (reduced AMP secretion)<br>● Wnt/TCF4 (reduced a-defensins)<br>● NOD2 mutations (reduced a-defensins)<br><br>"HBD2 and HBD3 are neighboring genes on chromosome 8p23.1. In healthy humans the DNA copy number of this gene cluster is highly polymorphic [45] In **colonic CD** however the median number of **HBD2 gene copies was only 3 per genome compared to 4 in UC** patients **and the healthy control group**. Accordingly, **patients with 3 or less HBD2 gene copies are more susceptible to phenotypically develop colonic CD than individuals with 4 or more gene copies** (odds ratio 3.06, 95% confidence interval 1.46–6.45). Additionally, these patients show a diminished HBD2 mRNA expression [44] .<br>**However, these data could not be confirmed in another cohort from New Zealand [46]"**<br><br>● a further defensin called HBD2 is not constitutively expressed in the uninflamed colon but is induced under inflamed conditions especially in UC [38]<br>● Remarkably the strong induction of HBD2 during inflammation observed in UC patients is attenuated in patients with acute inflamed colonic CD [37]<br>● This deficiency of HBD2 can be observed on the protein level in colonic CD, too [39]<br><br>"about 300- to 1,000-fold enhanced expression of HBD2 and HBD3 and other AMPs during inflammation implicates an **intact bacterial shield, an**d, as a matter of fact, we were able to show an increased antimicrobial activity of biopsy extracts from **UC** patients against various bacteria [42, 43]. However, **this overwhelming antibacterial activity might be dearly bought through enhanced inflammatory processes** mediated by the proinflammatory and chemokinelike effects of AMP.<br>In terms of modulating the mucus layer function the ad- ministration of phosphatidylcholine formulations could be promising agents in UC [53,54.	[8]
		● Alpha defensins<br>○ HNP 1–4<br>○ HD 5–6<br>● Beta Defensins<br>○ HBD 1<br>○ HBD 2–4	[249]

		“However, the mechanisms responsible for regulating HD5 and 6 expression in human Paneth cells is less clearly understood.”<br><br>“A major function of these defensins is believed to be protection of the adjacent epithelial stem cells at the base of the crypt,19. therefore continual expression of these defensins allows constant protection of the epithelial barrier integrity.”<br><br>“Pathway responsible for induction is not completely understood, however is known to involve NOD2 as an intracellular bacterial receptor that activates Nuclear Factor (NF)-kB, which in turn triggers transcription of b-defensin genes **17**”		
		“In mice, maturation of Paneth cell a-defensins from precursor pre-propeptides to their active form depends on proteolytic cleavage by matrix metalloproteinase 7 (MMP7; matrilysin), which is also expressed specifically in Paneth cells [29]. **Genetic deletion of MMP7 in transgenic mice completely abolishes the production of mature a defensins, and the demonstration that such mice have a measurable defect in intestinal antibacterial defense was an important milestone in establishing a role for Paneth cells in host defense** [30].”		[250]
		**THR**_NOD2<br><br>(AND NOT NOD2 mutation)	**17:** Culture of Epithelial Cells: HEK293 cells and primary keratinocytes: “Human beta-defensin-2 (hBD-2) is an antimicrobial peptide induced in various epithelia upon extracellular as well as intracellular bacterial challenge. Nucleotide-binding oligomerization domain protein 2 (NOD2/CARD15) is a cytosolic protein involved in intracellular recognition of microbes by sensing peptidoglycan fragments (e.g.muramyl dipeptide)”<br><br>“Together, these data suggest that NOD2 serves as an intracellular pattern recognition receptor to enhance host defense by inducing the production of antimicrobial peptides such as hBD-2.”<br><br>“**These observations further support the hypothesis that decreased production of antimicrobial peptides may promote bacterial-mediated inflammation in Crohn disease** (61). **Our data revealed that HEK293 cells overexpressing the 3020insC NOD2 variant (the major NOD2 mutation associated with Crohn disease) failed to induce hBD-2 upon stimulation with MDP**. This gives reason to hypothesize that hBD-2 expression might be dysregulated in patients with Crohn disease and could also offer an explanation for the recent findings regarding a lack of induction of the inducible beta-defensins hBD-2 and hBD-3 in Crohn disease as compared with ulcerative colitis (62).”<br><br>**<u>Comment</u>: U**NOD2 in the Boolean function because NOD2 is activated with the Antigen and DEF would be activated very quick to eliminate the Antigen. Introducing the **U**, NOD2 should be activated for longer to activate DEF in the network.	[251]
			-”In the presence of **functional Nod2**, Paneth cells sense the presence of bacteria or bacterial antigen and release **anti-microbial peptides**”	[30,31]<br>[248]

			-"The link between Nod2 mutations and **Paneth cell-derived defensins** was discovered through a number of human and rodent studies [74, 81, 82]."<br><br>-In humans, **CD patients** with ileal involvement **showed reduced levels of the Paneth cell- derived human α-defensins** HD-5 and 6 [82–84]. **This decrease in the amount of α-defensins occurs irrespective of Nod2 mutations, but levels are much more drastically reduce in CD patients harboring NOD2 mutations** [82]	
		IL22	**"Stimulation** of keratinocytes with **IL-22** resulted in **marked induction of genes** encoding proteins involved in antimicrobial host defense, including S100A7, S100A8, S100A9, **β-defensin-2 and β-defensin-3** (refs. 13,14,20,22)."<br><br>"In addition to r**egulating the expression of antimicrobial peptides and defensins**, IL-22 contributes to the expression of a range of genes encoding molecules involved in inflammatory responses, including IL-6, G-CSF, IL-1α, LPS-binding protein, serum amyloid A, α1-antichymotrypsin and haptoglobin 15,20–23"	[44]
			**MOUSE** "IL-22 expression was increased in murine dextran sulfate sodium-induced colitis. IEC express functional receptors for **IL-22**, **which increases the expression of** proinflammatory cytokines and promotes the innate immune response by increased **defensin expressio**n"<br><br>**HUMAN**: "**IL-22 promotes the intestinal barrier integrity in vitro through stimulation of IEC migration and defensin expression.** Overall, our data indicate a role for this cytokine system in protecting the intestinal barrier by enhancing IEC migration, suggesting an important function in intestinal inflammation and wound healing"<br><br>"IL-22 significantly increased cell proliferation (P <0.002) and phosphatidylinositol 3-kinase-dependent IEC cell migration (P < 0.00001) as well as mRNA expression of TNF-a, IL-8, and human b-defensin-2."<br><br>**Figure E:** after stimulation with IL-22 (100 ng/ml), human b-defensin-2 (hBD-2) mRNA is upregulated 6-fold in HT-29 cells as determined by semiquantitative PCR."	[126]
		IL17	"More notably, **neutralization of IL-17A in TH-17** cell supernatants **suppressed the induction of b-defensin** 2 **to the degree induced by TH0** supernatants, but neutralization of IFN-g did not **(Fig. 5e)"**<br><br>"(e)ELISA of **b-defensin 2** in supernatants of NHEKs **stimulated for 48** h with conditioned media from CD4+ T cells cultured with **IL-2 (TH0)** or **IL-23 (TH-17)** in the presence of control IgG, anti-IL-17A or anti-IFN-g"	[96]

			**Comment:** Th0 AND IL2 → Th0??	
		“Our group was able to show that patients with ileal CD have a **reduced expression of TCF4 and also a reduced expression of HD5 and HD6 (DEF),** resulting in a lower antibacterial activity of intestinal mucosal extracts from patients with ileal CD, implicating a functional relevance of these observations.”		[8]
		“In humans, **CD patients** with ileal involvement **showed reduced levels of the Paneth cell- derived human α-defensins** HD-5 and 6 [82–84] “		[248]
**DEF** = IL22 **OR** IL17 **OR** **THR**_NOD2				
32	IL2<br><br>Upregulated in CD (but cell cultures) [252]	Th0	**“IL-2 is produced primarily by antigen-specific CD4+** and CD8+ **T cells** following activation and mediates an autocrine/paracrine proliferative program through the IL-2 receptor (IL-2R).17”<br>“Antigen-activated T cells express the HA IL-2R and use low concentrations of IL-2 for proliferation and survival”	[243]
			17 “As evidenced by the results of the very hrst IL-2 experiments (1), resting T cells do not produce IL-2, nor are they capable of responding to IL-2 when it is added exogenously. It follows odlat signals emanating from the T cell antigen-receptor complex coordinate the transcriptional activation of both the IL-2 gene and the genes encoding IL-2 receptors (51)”	[253]
			“Interleukin-2 (IL2) is a protein initially identified as a T cell growth factor [1]. IL2 is mainly produced by activated CD4+CD25- (helper) T cells, and induces the proliferation of these and others cells like CD8+ T cells, B and NK cells [2].”	[254]
			[1] “**During steady-state conditions, IL-2 is produced mainly by CD4+ T helper (TH) cells in secondary lymphoid organs** (FIG. 1) and, to a lesser extent, by CD8+ T cells, natural killer (NK) cells and natural killer T (NKT) cells 3,4”<br><br>“Under certain conditions, IL-2 can also be synthesized in small amounts **by activated dendritic cells (DCs) and mast cells** 5,6”<br><br>“IL-2 production by CD4+ and CD8+ T cells is strongly induced following activation by antigen, although IL-2 synthesis by CD8+ T cells is comparatively weak and the responses of these cells often require help from CD4+ T cells.”<br><br>“‡ Fibroblast cell lines have been reported to express CD25 and CD122, but not γc 75,76” (IL2 R).”	[95]
			3: **“IL-2 is rapidly and transiently produced upon engaging the TCR and costimulatory molecules such as CD28 on naive T cells.** The transient nature of IL-2 secretion depends on transcriptional	[256]

			induction by TCR signals and stabilization of IL-2 mRNA by costimulatory signals, followed by transcriptional silencing of the IL-2 gene and rapid degradation of IL-2 mRNA **(30–34).”**<br>**Figure 1**<br><br>**4: Mice** [255]	
		DC	**5:** The most unanticipated finding of this study was that DCs produce IL-2 in a tightly regulated time-frame. Thus, the adjuvant property of bacteria is explained by induction, in DCs, not only of the up-regulation of costimulatory surface proteins and the maximization of the effi- ciency in presenting antigens 5, but also by induction of the production of costimulatory molecules such as IL-2. This seems to be a unique feature of DCs because macrophages are unable to produce IL-2 upon bacterial activation. Two waves of IL-2 production by DCs after bacterial encounter were observed. The first was 4–8 h after bacterial uptake and the second was 14–18 h after activation”.<br>**Figure 4**	[257]
		NK AND IL2	“IL-2 is produced by **antigen-activated CD4+ T** cells [13],**CD8+ T** cells, **NK** cells [14], and **NKT** cells [15]”<br><br>14: *(CD56bright NK which are only a 10% of NK)*	[58]
			“NK cells are present within the gut-associated lymphoid tissue including intraepithelial lymphoid compartment, intestinal lamina propria, Peyer’s patches, and mesenteric lymphoid nodes and **display a proinflammatory cytokine profile** (e.g., IFN-γ, TNF, **IL-2**, IL-17, and IL-22) in response to commensal enteric bacteria through the innate immune system and cytolytic activity, indicating that **intestinal NK cells play an important role** in mucosal innate immunity and tolerance [13–17].”<br><br>“IL-2 is a lymphocytotrophic cytokine that is involved in the growth and differentiation of T and B cells and enhances the cytolytic activities of NK cells.”	[1]
			13 nothing: [3]<br><br>14 “In conclusion, CD3+ IEL were capable of strong lytic activity after in vitro expansion with IL-2, comparable to IL-2–stimulated peripheral blood NK cells.” [258]<br><br>15<br>16<br>17	[3]
		T0_M	**(**24,25) “CD45RA−CCR7+ central memory T (**TCM**) cells: traffic to lymphoid tissues: produced more interleukin-2 (**IL-2**) “	[259]

		**"IL2 expression was significantly elevated in CD patients not treated with anti-TNFs in comparison to healthy controls and CD patients treated with anti-TNFs**, and similar in infliximab-treated patients and controls."<br><br>**Fig 2.** Comparison of cytokine expression by CD4+ PBL of CD patients with and without anti-TNF therapy as demonstrated by FACS analysis. Only significant (< 0.05) P values are demonstrated. PBL: peripheral blood lymphocytes; CD-anti-TNF treatment (n = 11): CD patients treated with anti-TNFs; CD-no anti-TNF treatment (n = 12): CD patients not receiving anti-TNF treatment; n: number of patients included in the experiment.<br><br>**Biopsy of colonic cells→ cultured cells→ IL2 measurement**		[252]
		**IL2** = Th0 **OR** (T0_M **AND** (MDP **OR** LPS **OR** PGN)) **OR** DC		
33	MACR<br><br>Upregulated in CD and UC<br>[30]	(TLR2 OR TLR4 OR NOD2)<br><br>PGN OR LPS	"APC function in the gut is in turn directly **shaped by the microbiota"**	[260]
			"**Monocytes** and circulating conventional DCs (cDCs) activated by **lipopolysaccharide (LPS) and peptidoglycan.."**	[39]
			MOUSE:<br>"**LPS-induced** production of inflammatory cytokines from **peritoneal macrophages**. Wild-type macrophages produced IL-6, tumour-necrosis factor-a (TNF-)and IL-12 in response to LPS"<br><br>"**PGN and LPS** activates macrophages that secrete IL-6, TNFa and IL-12"	[37]
			**"Lipopolysaccharide** binds to a variety of serum proteins that influence the macrophage-mediated proinflammatory response"	[49]
		IFNg	"(B) **Control or IFN-g-activated human macrophages** were treated…"	[59]
		IL15	"IL-15 is pivotal for modulating the functional **maturation** of macrophages. IL-15 not only **enhances the phagocytic activity of macrophages** but **also induces the production of proinflammatory factors** such as IL-1, TNF, MCP- 1 and IL-8 **[35]**"	[212]
			**35:** "We have shown that **IL-15**, in addition to its effect on T cells, also exhibits effects on monocytes, and may act as a proinflammatory cytokine inducing monocytes to secrete both PMN and monocyte chemotactic factors"<br><br>"...suggesting that **IL-15 stimulation of synovial macrophages** might contribute to the induction of IL-8 and MCP-1 production in synovial fluid"	[214]
		AND NOT IL10	"IL-10 **inhibits multiple macrophage and DC effector functions** and plays a critical role in limiting tissue injury during infections and in preventing autoimmunity by limiting the duration and intensity of	[177]

			immune and inflammatory reactions"	
			"**TLR2 is a potent inducer of anti-inflammatory interleukin-10 (IL-10)**, which critically i**nhibits multiple macrophage and dendritic cell (DC) effector functions,** thus limiting exaggerated immune responses."	[14]
			"I**L-10 inhibits multiple macrophage and DC effector functions** and plays a critical role in limiting tissue injury during infections and in preventing autoimmunity by limiting the duration and intensity of immune and inflammatory reactions (**Moore et al., 2001**)"	[59]
		NFkB	**"Exposure of macrophages to bacterial products such as lipopolysaccharide (LPS) results in activation of the NF-κB transcription factor**, which **orchestrates a gene expression programme that underpins the macrophage-dependent immune response."**<br><br>"Recognition of pathogen associated molecular patterns (PAMPS), including gram negative bacterial lipolysaccha-ride (LPS), results in activation of macrophages, leading to a plethora of biological responses required for shaping both the innate and adaptive arms of the immune response [2]. These effects are mediated through the release of chemokines and cytokines such as tumour necrosis factor α (TNF) and interleukin 1β (IL-1)."<br><br>"B**inding of LPS to toll-like receptor 4 (TLR4) activates two principal signalling pathways,** distinguished by their dependence on the adaptor molecules myeloid differentiation factor 88 (MyD88) or TIR-domain-containing adaptor inducing IFN-β (TRIF) [6-10]. Significantly, **both** the Myd88 and TRIF pa**thways result in activation of the transcription factor, nuclear factor κB (NF-κB), a central regulator of the LPS, cytokine and stress responses in many cell types, including macrophages** [8,9]."<br>8→ mice<br>9→ mice	[261]
			"The transcription factor Nuclear Factor (NF)-kB is involved in the transcriptional regulation of the IL-1b, IL-6, IL-8, TNF-a, iNOS and COX-2 genes.7,8"<br>Figures	[262]
			"Our systems biology approach revealed that network dynamics of MyD88 and TRIF signaling and of cytokine production and response govern the stimulus-specific autocrine and paracrine functions of TNF."<br><br>"I**n macrophages, TLRs use two adaptors that mediate the signaling events leading to proinflammatory cytokine production: the plasma membrane-proximal MyD88 and the endosomal membrane-proximal TRIF.** Whereas TLR9, the receptor for	[263]

			unmethylated CpG DNA, engages MyD88, TLR3, the receptor for dsRNA engages TRIF, and TLR4, the receptor for lipopolysaccharide (LPS) engages both. T**hese adaptors mediate the activation of tran-scription factors such as NFkB and IRF3, both of which have been implicated in the control of TNF production** (Drouet et al. 1991; Wesche et al. 1997; Yamamoto et al. 2003; Covert et al. 2005; Lee et al. 2009)."<br>**Figure 1: Model.**	
		IL23	"IL-23 is also expressed on the surface of macrophages and dendritic cells and may thereby con-trol barrier function and immune response against the commensal microflora in the gut. Consistent with this hypothesis, IL-23 is required for gut inflammation via innate immune mechanisms in T cell–deficient ani-mals9."	[125]
			"IL23 is produced by activated myeloid cells including macrophages and dendritic cells (DCs) following bacterial stimulation20 or via CD40 signalling,6 and drives increases in a number of inflammatory cytokines in the intestine in the absence of T cells, including TNFα, IFNγ, IL6 and IL17. **The IL23R is expressed by activated DCs and macrophages, and IL23 can induce production of inflammatory cytokines by macrophages." (**Ya están activados)	[42]
			**<u>Comment</u>**: Monocytes are Macrophages precursors. So as they present similar functions and in some experiments it is not clear what is the exact phenotype of the cells, we intended as MACR both.<br><br>**<u>Comment:</u>** Receptors activate MACR when they are activated by their ligands (as in DC node)	
			**MACR** = (NFkB **OR** (MACR **AND** (IFNg **OR** IL15))) **AND NOT** (IL10 **AND** MACR)	
34	DC<br>[4]<br>[30]	PGN OR LPS<br>(TLR2 OR TLR4)	"Monocytes and circulating conventional **DCs** (cDCs) **activated by lipopolysaccharide (LPS) peptidoglycan,** which ...."	[39]
			"The CD11c+ cells have phenoptypic, morphological and functional properties of myeloid DC, and disease-associated changes in these cells occur in IBD [19]. For instance, in **Crohn's disease** (CD), m**ore colonic CD11c+ DC expressed Toll-like receptor (TLR)-2 and TLR-4** and produced more interleukin (IL)-12/23p40."	[4]
			"Compared with DCs from healthy controls, more colonic DCs from patients with active IBD expressed TLR2 and TLR4 **(Figure 2B).** The increase in DCs expressing TLR was similar in ulcerative colitis and Crohn's disease."	[38]
		NOD2	"Cooney et. al. showed that **Nod2**, in the **presence of MDP**, induces **autophagy in dendritic cells** dependent on Rip2, ATG5, ATG7 and ATG16L1. They further showed that dendritic cells from patients harboring CD- associated NOD2 or ATG16L1 mutations are defective	[248]

			in bacterial handling and autophagy induction **[89]**"	
			**89**: "We demonstrate that **NOD2 induces autophagy in DCs** that is required for NOD2 mediated antigen presentation and bacterial handling and is defective in the presence of Crohn's disease–associated NOD2 or ATG16L1 variants."<br><br>**Comment:** If NOD2 is altered, and autophagy is altered in IBD patients, DC is not able to present Ag and start autoimmune reaction.	89: [264]
			"The **suppressive function of IL-10** involves the inhibition of **macrophages and DCs** ….."<br>Refers to [177]	[176]
		AND NOT IL10	[177]"IL-10 **inhibits multiple macrophage and DC effector functions** and plays a critical role in limiting tissue injury during infections and in preventing autoimmunity by limiting the duration and intensity of immune and inflammatory reactions".	[177]
			"**TLR2 is a potent inducer of anti-inflammatory interleukin-10 (IL-10)**, which critically i**nhibits multiple macrophage and dendritic cell (DC) effector functions,** thus limiting exaggerated immune responses."	[14]
		NFkB	"**To activate DC, danger signals use several common signal transduction pathways with major roles for mitogen-activated protein kinases (MAPKs) and nuclear factor kappa B (NF-kB).**"<br><br>"**Signals such as Toll-Like Receptors (TLR) agonists** or proinflammatory cytokines **are known to activate NF-KB in DC leading to production or expression of many inflammatory cytokines**, chemokines, immune receptors, and cell surface molecules (Verhasselt et al., 1999)."<br>(Verhasselt et al., 1999) → No habla de NFkB<br><br>"**NF-KB and MAPK pathways are the two major pathways involved in DC maturation induced by danger signals such as TLR agonists**."	[265]
			"Dendritic cells (DCs) produce an array of cytokines after detecting various immune adjuvants through pattern recognition receptors (PRRs). PRR signaling leads to activation of transcription factors such as NF-kB or interferon regulatory factors (IRFs) but after activation must be attenuated to avoid immunopathology and to maintain tissue homeostasis."<br><br>"... Their signaling can activate DCs to produce proinflammatory cytokines and type I interferons (IFNs) through a set of transcription factors including NF-kB and IFN regulatory factors (IRFs) [7–10]."<br>7→ do not mention DC<br>8 → mouse	[266]

			9 → mice<br>10 → do not talk about NFkB	
			“A crucial pathway for the maturation of DC by either in vivo or in vitro inflammatory stimuli involves the transcription factor NF-κB”	[267]
			**When DCs encounter microbial products that stimulate them through pattern recognition receptors, such as Toll-like receptors (TLRs), they mature**. This maturation is characterized by the upregulation of major histocompatibility complex (MHC) and co-stimulatory molecules, as well as the secretion of cytokines that shape the nature and amplitude of the adaptive response directed against the microbes. **Members of the NF-κB family...have an important role in this maturation process** and different combinations of homo- or heterodimers participate in various aspects of gene regulation1,2.	[268]
			“NF-kB is a master regulator of inflammation, and several NF-kB subunits have been described to control DC functions (7–11).”	[269]
		IL12	“The IL-12R is primarily expressed by activated T-cells and natural killer (NK) cells, but has been found to be expressed on dendritic cells (DCs) and B-cells as well[73,74]”	[70]
			73 → “do not mention that IL12 activates DC”	[244]
			74 → “Expression of IL-12R has been shown also on other cell types, such as DCs21 and B-cell lines 6”<br><br>**Comment:** DC express IL-12R, but how IL12 activates DC and its consequences are not known	[245]
		IL23	“I**L-23 is also expressed on the surface of macrophages and dendritic cells** and may thereby con-trol barrier function and immune response against the commensal microflora in the gut. Consistent with this hypothesis, IL-23 is required for gut inflammation via innate immune mechanisms in T cell–deficient animals9.”	[125]
			9 → Mice	[74]
		**89**: “We demonstrate that **NOD2 induces autophagy in DCs** that is required for NOD2 mediated antigen presentation and bacterial handling and is defective in the presence of Crohn’s disease–associated NOD2 or ATG16L1 variants.”<br><br>**Comment:** If NOD2 is altered, and autophagy is altered in IBD patients, DC is not able to present Ag and start autoimmune reaction.		89: [264]
		“In patients with active IBD, **DC are reduced in number in the peripheral blood** [15], but i**ncreased in the intestinal tissue** [16,17], suggesting DC recruitment to and/or retention within the gastrointestinal (GI) tract during inflammation”		[4]<br>[30]

		“**Macrophages and dendritic cells in the lamina propria are increased in absolute number** and have an activated phenotype in both forms of IBD, but have been studied in greater detail in Crohn’s disease.”		
**DC** = NFkB **AND NOT** (IL10 **AND** DC)				
35	IEC_MICA/B<br><br>Upregulated in CD<br><br>[62]	“Among known NKG2DLs, **MICA and MICB are the most polymorphic. To date 100 alleles are known for MICA and 40 for MICB**. This corresponds to **79 and 26 unique protein sequences,** respectively.”<br><br>“Genetic variations are mainly represented by SNPs in exons 2-4 coding for the extracellular a1-3 domains. There are a total of 62 and 25 SNPs for MICA and MICB, respectively, and, except for 4 positions in MICA,”<br><br>“In MICA, there is a notable absence of coding polymorphism between residues 40 and 89 which could be explained by the important involvement of this segment in the binding with NKG2D”<br><br>“Several polymorphisms/mutations have major impacts on the protein structure and/or stability: (i)”<br><br>“It is also possible that under certain environmental conditions, the loss of MIC expression confers a selective advantage**(31)”**		[270]
		CD8+ T cells	“We observed that **MICA was induced upon activation of CD8+ T cells**. MICA expression was not significantly enhanced by NKG2D costimulation compared with anti-CD3 stimulation alone. We also observed an increase in the message levels of ULBP-1, -2, and -3.”	[63]
		TGFb	**“TGFb inhibits the transcription of** the NKG2D ligand **MICA**.”	[271]
		IEC_MICA_B AND TNFa	“**MICA... and its expression is upregulated by tumor necrosis factor-a** [7]”	[272]
			**7:** “**Tumor necrosis factor-a** and radiation **up-regulated expression of MICA/B and ULBP** proteins in vitro on skin and **intestine epithelial cell lines** and ex vivo in normal skin explants”.<br>**“MICA/B** and **ULBP1-3** molecules were **significantly up-regulated** by TNF-A”<br>“cell lines derived from spontaneously immortalized but **non tumorigenic human keratinocytes (HaCat)** and from **colorectal carcinoma (HT-29)”**	[273]
		“An abundance of sMICA is also found in rheumatoid arthritis patients that, intriguingly, fail to down-modulate NKG2D probably due to the compensating NKG2D upregulation by the activity of the pro-inflammatory cytokines, TNFa and IL-15”		[65]
		“Are stress-inducible molecule, especially in the context of bacterial infections, and are overexpressed in the mucosa of patients with CD.25”		[62]

<table>
<tr><td></td><td></td><td colspan="2">MICA expression was significantly increased on IECs in CD</td><td>[274]</td></tr>
<tr><td colspan="5">IEC_MICA_B = ((LPS OR MDP OR PGN) OR (IEC_MICA_B AND TNFa)) AND NOT TGFb</td></tr>
<tr><td rowspan="2">36</td><td rowspan="2">IEC_ULPB1-6

Upregulated in CD [62]</td><td>CD8+ T cells</td><td>“We observed that MICA was induced upon activation of CD8+ T cells. MICA expression was not significantly enhanced by NKG2D costimulation compared with anti-CD3 stimulation alone. We also observed an increase in the message levels of ULBP-1, -2, and -3.”</td><td>[63]</td></tr>
<tr><td colspan="2">“NKG2DL are stress-inducible molecules, especially in the context of bacterial infections, and are overexpressed in the mucosa of patients with CD.25”</td><td>[62]</td></tr>
<tr><td colspan="5">IEC_ULPB1_6 = CD8_NKG2D AND (LPS OR MDP OR PGN)</td></tr>
<tr><td rowspan="9">37</td><td rowspan="9">CD8_NKG2D</td><td>IL15 AND (IL7 OR IL2)</td><td>“We compared the effect of IL-2 vs IL-7 plus IL-15 on activated naive CD8+ T cells. Analyses at day 7 showed that NKG2D costimulation in both conditions increases the number of dividing cells, albeit more so in the presence of IL-7 + IL-15.”</td><td>[63]</td></tr>
<tr><td rowspan="2">AND NOT (IL21 AND IL2)</td><td>“IL-21 plus IL-2 results in the downregulation of NKG2D”[67]</td><td>[275]</td></tr>
<tr><td>[67] “We report that culture of human primary NK and CD8+ T cells with IL-21 in combination with IL-2 results in significant reduction of the cell surface expression of NKG2D, compared with that in cells treated with IL-2 alone.”
Figure 2</td><td>[276]</td></tr>
<tr><td rowspan="2">TGFb</td><td>“TGFb1 is a cytokine that has been reported to downregulate cell surface expression of NKG2D leading to a reduction in NKG2D-mediated cytotoxicity.”70
70→ Human NK cells</td><td>[275,277]</td></tr>
<tr><td>The TGFb mediated down-modulation of the NKG2D expression was protected by both IL-2 and IL-18 in a dose-dependent manner.
AND NOT (IL2 AND IL18) but only through the JNK pathway</td><td>[278]</td></tr>
<tr><td rowspan="2">AND NOT (THR_IEC_MICA/B OR THR_IEC_ULPB1-6)</td><td>“Another described mechanism for NKG2D downregulation is attributed to overexposure to NKG2D ligands.”</td><td>[275]</td></tr>
<tr><td>76“Here we show that binding of MIC induces endocytosis and degradation of NKG2D.
Figure 4</td><td>[279]</td></tr>
<tr><td colspan="2">“NKG2D is present in CD8 T cells (αβ), gamma-delta T cells, and natural killer cells”</td><td>[280] [274]</td></tr>
<tr><td colspan="2">“NKG2D is also expressed by CD8 T cells and T cells and provides important costimulatory signals in T cell-mediated adaptive immune responses by amplifying T cell cytokine production and proliferation”</td><td>[281]</td></tr>
</table>

CD8_NKG2D = (LPS OR PGN OR MDP) AND NOT ((UIEC_MICA_B OR UIEC_ULPB1-6 OR (IL21 AND IL2)) AND CD8_NKG2D)				
38	NK_NKG2D	“**Natural killer group 2, member D: NKG2D** is a type II transmembrane glycoprotein containing an extracellular C-type lectin domain (14, 15). In human, it is encoded by the KLRK1 (killer cell lectin-like receptor subfamily K, member 1) gene located within the NK gene complex (NKC) on chromosome 12, at 12p13.2.”<br><br>“NKG2D is a disulfide-linked homodimer e**xpressed on all NK cells, most NKT cells, subsets of cd T cells, all human CD8+ ab T cells...**(16–18). **Upon association with its ligands, NKG2D binds to an adapter molecule, DAP10 in human** (19)......This **transmits an activatory signal which ultimately triggers lymphocyte cytotoxic granules polarization and degranulation,** cy**tokine production, prolif-eration, and survival (21, 22)**.”<br><br>“In **contrast to its ligands, NKG2D is not polymorphic**. Only two single nucleotide polymorphisms (SNPs) with a minor allele frequency >0.01 have been reported in the coding sequence of the gene: the synonymous **rs1049172** and the non-synonymous **rs2255336**.”<br><br>“Other n**on-coding polymorphisms** in and around the gene have been described. The most studied is **rs1049174** in the 30-untranslated region (30UTR) of the gene and enables the distinguishing of two haplotypes:<br>**HNK1 (high NK cytotoxicity) and LNK1 (low NK cytotoxic-ity)**”.<br><br>“H**ealthy cells do not induce the activatory NKG2D signaling pathway in NK cell**s. The **absence of activation results** from a **balance** between the a**bsence or low expression of NKG2DLs on healthy cells and inhibitory signals mediated by inhibitory receptors on NK** cells. After a **cellular stress provoked by stimuli such as bacterial products**, oncogenes, viral infections, oxidative stress, ionizing radiations or DNA damages, **NKG2DLs are typically upregulated (upper arrow)**. This **upregulation leads to the lysis of the target cell via the activation of NKG2D signaling. Inappropriate expression of the ligands, however, may lead to the development of autoimmunity**. When stressed cells fail to upregulate the ligands there is too little NKG2D signaling (lower arrow), which can lead to the development of cancer or the spread of infection.”<br>**Figure 1**		[270]
		AND NOT (IL21 AND IL2)	“IL-21 plus IL-2 results in the **downregulation** of NKG2D”**[67]**	[275]
			**[67]** “We report that **culture of human primary NK and CD8+ T cells with IL-21** in combination with IL-2 results in **significant reduction of the cell surface expression of NKG2D**, compared with that in cells treated with IL-2 alone.”<br>**Fig 2**	[276]
		TGFb	**“TGFb1** is a cytokine that has been reported to **downregulate cell surface expression of NKG2D** leading to a reduction in NKG2D-mediated cytotoxicity.”**70**	[275]
			**70** “TGFb Induces Specific Down-Regulation of NKp30 and NKG2D	[277]

			Surface Expression in Human NK Cells" **fig 1**	
		THR_IEC_MICA/B THR_IEC_ULPB1-6	"Another described mechanism for **NKG2D downregulation** is attributed to **overexposure to NKG2D ligands**."**76**	[275]
			**76**"Here we show that binding of MIC induces endocytosis and degradation of NKG2D."<br>**Fig 4**	[279]
		AND NOT (NK_NKG2D AND IFNg)	"However, Zhang et al. [69] observed an inhibitory effect of high concentrations of IFNg on NKG2D expression with a resultant decrease in cytotoxicity toward NKG2D-ligand expressing targets."<br><br>"The fact that IFNg can downregulate NKG2D expression may imply that there is a negative feedback loop in which IFNg can 'dampen' NKG2D-mediated NK cell activation."	[282]
		"NKG2D is present in CD8 T cells (αβ), gamma-delta T cells, and **natural killer cells**".		[274]
**NK_NKG2D =** (LPS OR PGN OR MDP) AND NOT ((TGFb OR **THR**_IEC_MICA_B OR **THR**_IEC_ULPB1-6 OR (IL21 AND IL2)) AND NK_NKG2D)				
39	CD4_NKG2D<br><br>Upregulated in CD [274]	CD4_NK2D AND (IL15 AND TNFa)	"In addition, **IL-15 stimulation in conjunction with TNFa**, has also been shown **to induce an unusual subset of NKG2D+CD4+** T cells which have been described in patients with rheumatoid arthritis." **65**	[275]
			**65** "Thereafter, **NKG2D decreased gradually** unless the culture was replenished with **fresh IL-15.**" **Figure 3**<br><br>"Induction of NKG2D on CD4 T cells by IL-15 and TNF-a .(A and B)" **Fig 2**<br><br>"By contrast, RA patient serum **failed to down-modulate NKG2D because of the presence of TNF-a and IL-15 TNF-a (Fig. 6)**. Thus, **the ligand-induced down-modulation of NKG2D in RA patients was compensated by the opposite effect of its cytokine-mediated induction**."	[65]
		AND NOT THR_ IEC_MICA_B	"An **abundance of sMICA** is also found in these patients that, intriguingly, **fail to down-modulate NKG2D** probably **due to the compensating NKG2D upregulation by the activity of the pro-inflammatory cytokines, TNFa and IL-15**."	[65]
		AND NOT (CD4_NKG2D AND IL10)	"Exposure to **IL-10**, but not to IL-12 and IFN-G resulted in **less pronounced and variable induction of NKG2D**. T cell antigen receptor complex stimulation with anti-CD3 transiently induced NKG2D on some CD4 T cells (data not shown)."	[65]
		"**CD4+ T cells expressing NKG2D are specifically up-represented** in CD as compared with UC and with HCs"		[274]

<table>
<tr><td rowspan="2"></td><td rowspan="2"></td><td colspan="2">A subset of CD4+ T cells expressing NKG2D was increased in the lamina propria from patients with CD compared to controls or UC</td><td>[283]</td></tr>
<tr><td colspan="2">“CD4+NKG2D+ T cells expand in the lamina propria (LP) and the peripheral blood (PB) of patients with CD, but not in patients with UC or in healthy controls (HCs)”</td><td>[62]</td></tr>
<tr><td colspan="5">CD4_NKG2D = (LPS OR PGN OR MDP OR (CD4_NKG2D AND (IL15 OR TNFa))) AND NOT ((IL10 OR UIEC_MICA/B OR UIEC_ULPB1-6) AND CD4_NKG2D)</td></tr>
<tr><td rowspan="3">40</td><td rowspan="3">FIBROBLAS T

Upregulate d CD and UC
[284]</td><td>MACR AND (IL4 OR IL13 OR TGFb) AND NOT (IFNg OR IL12)</td><td>“A number of cell types have been implicated in intestinal fibrosis, but the exact cell type responsible for the fibrotic response is unknown. It has been suggested that the fibroblasts, myofibroblasts and smooth muscle cells are in a state of continuous differentiation and dedifferentiation, rather than a single one of them being exclusively responsible 4. There is, however, substantial evidence for a critical role of fibroblasts in the development of Crohn’s strictures 15,37,38”

“human fibrocytes represent a transitional stage of differentiation of this monocyte subset into mature fibroblasts and myofibroblasts in tissues”
Figure 1 :fibrocytes→ fibroblast, myofibroblast and adipocytes
“Bone marrow-derived circulating fibrocytes migrate to, and extravasate into, sites of tissue injury, where they differentiate into fibroblasts/myofibroblasts, and participate in the generation of extracellular matrix (ECM) 64,73,80.”

“Toll-like receptor (TLR) 2 agonists inhibit fibrocyte differentiation 83, and TLR-2 stimulation effectively preserves colonic tight junctions against stress-induced damage and susceptibility to inflammation 84.” ¿¿?

“Fibrocyte differentiation from CD14+ precursors into fibroblasts and myofibroblasts is stimulated by profibrotic cytokines (TGF-β1, endothelin-1, IL-4 and IL-13) and inhibited by antifibrotic (IFN-γ and IL-12) cytokines as well as serum amyloid protein (Fig. 1) 60.”</td><td>[285]</td></tr>
<tr><td>IL2</td><td>“Although addition of IL-2 to fibroblast cultures did not significantly alter growth kinetics of these cells, the 11-2R complex displayed by fibroblasts appeared to be functional in that addition of IL-2 to these cells led to enhanced expression of the JE gene coding for the monocyte chemoattractant protein-1 (MCP-1). Enhancement of fibroblast MCP-l/JE gene expression by IL-2 appeared to result from delayed MCP-l/JE mRNA decay rather than as a consequence of an acceleration of the MCP-1/JE gene transcription”</td><td>[286]</td></tr>
<tr><td>MACR AND (IL4 OR IL13)</td><td>60: “we find that when added to human PBMCs cultured in serum-free medium, the profibrotic cytokines IL-4 and IL-13 promote</td><td>[287]</td></tr>
</table>

		AND NOT (IFNg OR IL12)	**fibrocyte differentiation** without inducing fibrocyte or fibrocyte precursor proliferation. We also find that the potent, **antifibrotic cytokines IFN-g and IL-12 inhibit fibrocyte differentiation**."<br>"The profibrocyte activities of IL-4 and IL-13 and the fibrocyte-inhibitory activities of IFN-g and IL-12 counteract each other in a concentration-dependent manner." **Figure 2**	
		AND NOT PGN	**83:** "However, **all TLR2 agonists tested inhibited fibrocyte differentiation without any significant effect on cell survival**. Adding TLR2 agonists to purified monocytes had no effect on fibrocyte differentiation. However, **some TLR2 agonists caused PBMCs to secrete a factor that inhibits the differentiation of purified monocytes into fibrocytes.** This factor is not interferon (IFN)-a, IFN-g, interleukin (IL)-12, aggregated immunoglobulin G (IgG) or serum amyloid P (SAP), factors known to inhibit fibrocyte differentiation. **TLR2 agonist-treated PBMCs secrete low levels of IL-6, TNF-a, IFN-g, granulocyte colony-stimulating factor and tumor growth factor b1, but combinations of these factors had no effect on fibrocyte differentiation from purified monocyte**s."<br><br>"Our results indicate that **TLR2 agonists indirectly inhibit fibrocyte differentiation and that, for some TLR2 agonists**, this inhibition involves other cell types in the PBMC population secreting an unknown factor that inhibits fibrocyte differentiation. Together, **these data suggest that the presence of some bacterial signals can inhibit fibrocyte differentiation and may thus slow wound closure**."	[288]
		**Upregulated in IBD**<br><br>"Hypoxia in the inflammatory area stimulates angiogenesis by up-regulation of vascular endothelial growth factor fibroblast growth factor and tumor necrosis factor-α."<br><br>"Structural changes in vascular endothelium include capillary and venule remodeling and proliferation of endothelial cells[28] . Angiogenesis is a crucial process that sustains chronic inflammation in the gastrointestinal tract. Numerous inflammatory cell types, including macrophages, lymphocytes, mast cells, and fibroblasts, produce angiogenic factors and promote pathological angiogenesis in inflammatory Tissues[16,29] . Hypoxia in the inflamed area stimulates tissues [16,29]. angiogenesis by **upregulating vascular endothelial growth factor (VEGF), fibroblast growth factor,** and . Rutella et al[30] suggested that inflamed TNF?α[29] HIMECs recruit platelets to produce higher levels of pro?angiogenic VEGF?A and CD40 ligand (CD40L) in patients with active ulcerative colitis (UC) and Crohn's disease (CD)[30]"		[289]

			“Intestinal fibrosis is a common and serious complication of the inflammatory bowel disease (IBDs).”<br><br>“Intestinal fibrosis is a major complication of the inflammatory bowel diseases (IBD) and although inflammation is necessary for its development, it would appear that it plays a minor role in its progression as anti-inflammatory treatments in IBD do not prevent fibrosis once it has sta<br><br>“Myofibroblasts secrete ECM proteins and promote an altered cytokine milieu that supports the fibrotic process. **Under normal conditions the fibrotic matrix is degraded by matrix metalloproteinases (MMPs), myofibroblasts apoptosis, or reverts to a non-activated state**, **while the epithelium undergoes repair. Thus intestinal fibrosis is characterized by abnormal ECM deposition by activated myofibroblasts** 10,13–17 and **constitutive activation of collagen secreting myofibroblasts is ultimately responsible for increased tissue stiffness and progressive organ dysfunction.**18 This then is enhanced by the innate and adaptive immune systems, which promote fibrogenesis through the differentiation, recruitment, proliferation and activation of ECM-producing myofibroblast progenitors.11,12”	[284]
			<u>**ADDITIONAL INFORMATION:**</u><br>Node FIBROBLAST includes: FIBROBLAST AND MYOFIBROBLAS<br>Node MACR includes MONOCYTES (MACR precursor).<br><br>Figure 1: monocytes → fibrocytes→ fibroblast, myofibroblast and adipocytes<br>“Bone marrow-derived circulating fibrocytes migrate to, and extravasate into, sites of tissue injury, where they differentiate into fibroblasts/myofibroblasts, and participate in the generation of extracellular matrix (ECM) 64,73,80.”	[285]
**FIBROBLAST** = ((MACR **AND** (IL4 **OR** IL13 **OR** TGFb)) **OR** IL2) **AND NOT** ((IFNg **OR** IL12) **AND** FIBROBLAST))				
41	MMPs<br><br>up-regulated in IBD<br><br>[290,291]	MACR AND TNFa	“**TNF-α** as well, and this is the central cytokine which **induces the differentiation of stromal cells into myofibroblasts and promotes their production of matrix metalloproteinases (MMPs)**, a class of tissue-degrading enzymes which also induce enterocyte apoptosis by digesting the basement membrane **[95]**.”	[150]
			**[95]** **“**Proinflammatory mediators **activate macrophages** and induce them to release interleukin-12 (IL-12), which reduces Th1 cell apoptosis causing lympho accumulation and sustaining the inflammatory process **and tumor necrosis factor-α (TNF-α)** in turn, **promotes the differentiation of lamina propria stromal cells into activated myofibroblasts, which, together with lamina propria mononuclear cells** (LPMCs), start **producing large and unbalanced amounts of MMPs”**	[290]
		FIBROBLAST	“Of note, **IL-21 and IL-17A**, which have both been shown to be up-	[290]

		AND (IL21 OR IL17 OR IL1b OR TNFa)	regulated in inflamed mucosa in Crohn's disease (Monteleone et al. 2005;Rovedatti et al. 2009), **induce a marked increase in MMP production by intestinal myofibroblasts** in synergy with either **TNF-α or IL-1β** (**Monteleone et al. 2006;Bamba et al. 2003).”**<br><br>“Upon activation, **myofibroblasts** express increased amounts of MT1-MMP (MMP-14) and MMP-2, which increase the migration of these cells through the synthetic matrix and might therefore **enhance their wound-healing potential** (Pender et al. 2000)”	
			**Monteleone et al. 2006:**<br>“Moreover, **IL21 synergises with tumour necrosis factor a to increase synthesis of MMP** synthesis”<br>“we have shown that I**L21 increases MMP production by fibroblasts**”<br>“These data suggest that inflammatory cytokines such as **TNFa may transcriptionally increase MMP** production, but this effect is amplified by **IL21 at the post-transcriptional level” Figure**<br><br>**“Indeed, molecules such as IL1b, IL6, IL17, IL22 and TNFa, all of which could regulate MMPs**, are produced in excess in Crohn's disease 2, **5**, 10 18 33 34”	[292]
		FIBROBLAST AND (IL17 OR TNFa OR 1ILb)	**5:** “In the mucosa, MMP-1, -3 and -9 are highly expressed by **myofibroblasts**, which **are activated by mediators such as TNF-a, IL-12 or IL-17** “produced by the nearby T cells and macrophages” **Figure 1**: IL12 do not have reference<br><br>“IL-17, is upregulated in active IBD [42,43]. It stimulates inflammatory responses via nuclear factor-kB and mitogen-activated protein kinase pathways in myofibroblasts [44]. **IL-17 induces a marked increase in the secretion of MMP-3 by colonic subepithelial myofibroblasts in synergy with either IL-1b or TNF-a** [45].”	[293]
			**45:** “We have recently shown M**MP-1 and MMP-2 secretion in response to IL-1b and tumor necrosis factor (TNF)-α** in these cells”<br>“Previous studies have demonstrated that IL-1b and TNF-α are powerful stimulators of MMP-1 and MMP-3.23,27”<br>“In previous reports, MMP-3 secretion was shown to be tightly regulated by IL-1b and/or TNF-α, mainly derived from monocytes/macrophages.23,24,26,27”<br>“IL-17 itself was a weak stimulator of MMP secretion as compared with IL-1b and TNF-α, **IL-17 enhanced the IL-1b- and TNF-α-induced MMP-3”**<br>**Figure**	[294]
**MMPs** = (MACR **AND** TNFa) **OR** (FIBROBLAST **AND** (IL21 **OR** IL17 **OR** IL1b **OR** TNFa))				
42	PERFOR	(IL2 OR IL15	**IL-2 and IL-15** equally **lead to** the **upregulation of perforin and**	[58]

	Altered in CD<br><br>PERF1 mutations<br><br>[7]<br>[295]	) AND NK	**granzyme B** expression as well as cytokine production including IFN-g, TNF-a, and granulocyte-macrophage colony- stimulating factor (GMCSF) **by NK** cells.<br>● Review with no references.<br>● ¿Related to NK induction?	
		CD4_NKG2D<br><br>Th0	"**CD4+CD28- T cells** resemble **NKT** cells as they secrete large amounts of IFNG and **express perforin and granzyme B**, which confer cytotoxic capacity"	[65]
			"Besides classical cytotoxic cells (i.e. CD8+ ab T cells, cd T cells, NK cells), also **CD4+ T** cells develop secretory granules when activated and expanded in vivo or in vitro [24, 25]"	[5]
		CD8_NKG2D	"When **CD8+ T cells encounter their cognate antigenic peptide** presented on MHC I molecules with their T-cell receptors (TCR), the differentiation into cytotoxic effectors is manifested by the synthesis of cytotoxic granules and their constituent proteins [7–9]"<br>"In **T cells, tonic signals set basic threshold levels and the TCR signal is further fine-tuned by a variety of activating or inhibiting costimulatory receptor**s (also **including NKG2D**) and the local cytokine milieu."	[5]
		NK_NKG2D	"These findings support **NK cell activation through NKG2D/NKG2D ligand interaction** as the possible mechanism involved in the **perforin/granzyme-mediated** killing of **myeloma cells**."<br>"Plate-bound NKG2D-specific did stimulate the release of granzymes that participate in the cytotoxicity reaction"	[64,296]
		NK	**"NK cells** and supposedly is associated with the same lysosomal compartment that is used for storage and **release of perforin and granzymes** [8, 15, 29]".	[5]
			"**NK cells kill their target cells** through two major pathways, both requiring close contact between NK cells and the target cells. In the first pathway, **cytoplasmic granule toxins including perforins and granzymes are secreted by exocytosis and together induce apoptosis of the target cells**."	[1]
		"common polymorphism in the perforin (PRF1) gene that encodes moderate-to-severe dysfunction of the perforin protein"<br>"mild changes in perforin activity, as occurs in carriers of hypomorphic PRF1 mutations, might contribute to immune-based pathology over time."		[7]
		"Our data define a global methylation profile characteristic of ileal CD.**PRF1**... Gene ontology analyses implicated immunity -relatedpathways as targets of epigenetic modification"		[295]
**PERFOR =** NK OR NK_NKG2D				
43	GRANZB	NK	"**NK cells kill their target cells** through two major pathways, both requiring close contact between NK cells and the target cells. In the	[1]

	Overexpressed in both CD and UC tissues (19)<br><br>[6]		first pathway, **cytoplasmic granule toxins including perforins and granzymes are secreted by exocytosis and together induce apoptosis of the target cells**."	
		CD8_NKG2D	"GrB is produced not only by **NK cells, CD8+ T** lymphocytes, and B cells, but also by **plasmacytoid dendritic cells**, mast cells, basophils, and CD34 hematopoietic progenitor cells (26)."<br><br>**26:** Refers to B cells	[6]
			In CD8+ cells, exocytosis of perforin- and granzyme-containing cytoplasmic granules appears to account for the cytotoxic activity observed [12].<br>**Figure 5**	[297]
		(DC AND IL10) AND NOT (LPS OR PGN))	"Here we demonstrate that **human pDCs can be an abundant source of GrB** and that such **GrB pDCs potently suppress T-cell proliferation in a GrB- dependent**, **perforin-independent manner,** a process reminiscent of regulatory T cells. we find that the **immunosuppressive cytokine IL-10 enhances, while Toll-like receptor agonists and CD40 ligand strongly inhibit, GrB secretion by pDCs"**<br>**Figure 3**<br><br>**Comment:** we define DC node as being composed by DC, pDC. We do not include and IL10 because it inhibits DC node.	[298]
		IL21	"Is thus conceivable that **IL-21 is one of the positive regulators of GrB**- expressing CD19+ cell differentiation in IBD, as this cytokine is overproduced in both CD and UC tissues (**19**)"<br><br>**Comment:** CD19: Is a protein that in humans is encoded by the CD19 gene. It is found on the surface of B-cells, a type of white blood cell.<br><br>IL21 AND Bcell	[6]
			**19** Nothing related to GrB	[299]
		NK_NKG2D	"These findings support **NK cell activation through NKG2D/NKG2D** ligand interaction as the possible mechanism involved in the **perforin/granzyme-mediated killing of myeloma cells**."<br>"Plate-bound **NKG2D-specific did stimulate the release of granzymes** that participate in the cytotoxicity reaction"	[64,296]
**GRANZB =** CD8_NKG2D OR NK OR NK_NKG2D OR (DC AND NOT (LPS OR PGN))				
--	Network output: MMPs	IFNg, TNFa, IL21, IL17, IL13	IFNg, TNFa, IL21, IL17, IL13	[150]

	CDAI	IL13<br>UC	Cytokines such as IL-13 also increase epithelial claudin 2, which makes the gut leakier thereby allowing molecules to move into the lumen and vice versa a **(Prasad et al. 2005;Heller et al. 2005)**	[290]
			"(**IL-13) is the key effector cytokine in ulcerative colitis stimulating apoptosis** and upregulation of claudin-2 expression."<br><br>"The key **effector cytokine in ulcerative colitis was identified to be IL-13,** which is a potent inductor of apoptosis in our HT-29/B6 cell culture model (Fig. 1).1 These apoptotic events and a significant IL-13 dependent arrest in epithelial restitution resulted in epithelial lesions such as micro erosions, which are common in ulcerative colitis already at early stages of the inflammation."<br><br>"In addition, we could show that the **proinflammatory cytokines TNFα and IL-13 can facilitate bacterial uptake in intestinal epithelium**."	[300]
		GRANZB	"In conclusion, this is, to our knowledge, the first study to show that intestinal plasma cells express **GrB and have the ability to kill epithelial cells**, thus suggesting their involvement in **IBD-associated intestinal epithelial damage"**	[6]
		PERFOR	"Exocytosis of **perforin- and granzyme-**containing cytoplasmic granules appears to account for the **cytotoxic activity observed** [12]."	[297]
		MMPs	**"**Faecal MMP-9 levels were reported to correlate with the overall Mayo and endoscopic scores, serum CRP, and faecal calprotectin levels in UC patients[198]. calprotectin levelsin UC patients[198].As a biomarker, faecal MMP-9 also has potential in recognising severity of pouchitis and, to a lesser extent, CD where correlation with the SES (simple endoscopic score) CD was not statistically significant but overall correlations were better than calprotectin [199]."	[301]
			"However, in intestinal immune-mediated disorders, pro-inflammatory cytokines induce the up-regulation of **proteases**, which become the end stage effectors of mucosal damage by **destroying the epithelium and basement membrane integrity and degrading the extracellular matrix of the lamina propria to produce ulcers**. Protease-mediated barrier disruption in turn results in increased amounts of antigen crossing into the lamina propria, driving further immune responses and sustaining the inflammatory process."<br><br>**"as has been repeatedly and consistently observed, mucosal MMP levels correlate positively with the intensity of gut inflammation (Ravi et al. 2007).**<br>**Dysregulated metalloproteinase expression is a consequence of the inflammatory process and has a functional role in determining mucosal lesions in IBD."**	[290]

			-Epithelium-derived matrilysin (**MMP-7,** (expression is dependent on exposure to gut bacteria is **necessary for the process of reepithelialization after injury**<br>-Colla- genase 1 (MMP-1), MMP-7 and stromelysin 2 (MMP-10) expression by intestinal epithelial cell lines **is up-regulated by epidermal growth factor, TGF-β and IL-1β** (Salmela et al. 2004)<br>-**MMP-7 activates intestinal pro-α- defensins, which are needed for the clearance of pathogenic**<br>-**When produced in excess, MMP-7 can disrupt the gut mucosal barrier**. MMP-7 can degrade the epithelial adherens junction protein E-cadherin (Noë et al. 2001). MMP-7 is also highly overexpressed in inflamed IBD mucosa and its levels correlate with disease activity in ulcerative colitis (Salmela et al. 2004; Matsuno et al. 2003)<br>-the epithelium at the edge of intestinal ulcers is strongly positive for **MMP-7 and MMP-10** and disruption of the basement membrane below the MMP-7-positive epithelial cells has been described in IBD.<br>-**The effect of proteases on the intestinal barrier can switch from protective to disruptive not only because of increased local concentrations but also as a consequence of altered localization within the mucosa attributable to changes in the main producing cell types**<br>**-**Under inflammatory conditions, the up-regulation of a number of proteolytic enzymes produced in the gut lamina propria has been shown to exert a harmful effect on the intestinal barrier (Fig. 3). Protein extracts from both inflamed and ulcerated intestinal mucosa of patients with either Crohn's dis or ulcerative colitis are characterized by an increased expression of MMP-1, MMP-2, MMP-3, macrophage metalloelastase (MMP-12), collagenase 3 (MMP-13) and (MMP-14) compared with normal gut and all these enzymes can be produced by stromal and immune cells in the lamina propria.<br>-Although MMP inhibitors have been unsuccessful in phase III clinical trials in cancer treatment, it is premature to conclude that the modulation of protease activity has no therapeutic potential in inflammatory diseases, especially considering our increasing knowledge about the non-matrix targets of these enzymes and the development of better and more specific inhibitors	
**CDAI**= MMPs				

References

1. Yadav PK, Chen C, Liu Z. Potential role of NK cells in the pathogenesis of inflammatory bowel disease. J Biomed Biotechnol. 2011;2011: 348530.

2. Martín-Fontecha A, Thomsen LL, Brett S, Gerard C, Lipp M, Lanzavecchia A, et al. Induced recruitment of NK cells to lymph nodes provides IFN-γ for TH1 priming. Nat Immunol. Nature Publishing Group; 2004;5: 1260–1265.

3. Sanos SL, Diefenbach A. Isolation of NK cells and NK-like cells from the intestinal lamina propria. Methods Mol Biol. 2010;612: 505–517.

4. Ng SC, Plamondon S, Al-Hassi HO, English N, Gellatly N, Kamm MA, et al. A novel population of human CD56+ human leucocyte antigen D-related (HLA-DR+) colonic lamina propria cells is associated with inflammation in ulcerative colitis. Clin Exp Immunol. 2009;158: 205–218.

5. Lettau M, Kabelitz D, Janssen O. Lysosome-Related Effector Vesicles in T Lymphocytes and NK Cells. Scand J Immunol. 2015;82: 235–243.

6. Cupi ML, Sarra M, Marafini I, Monteleone I, Franzè E, Ortenzi A, et al. Plasma cells in the mucosa of patients with inflammatory bowel disease produce granzyme B and possess cytotoxic activities. J Immunol. 2014;192: 6083–6091.

7. Voskoboinik I, Whisstock JC, Trapani JA. Perforin and granzymes: function, dysfunction and human pathology. Nat Rev Immunol. 2015;15: 388–400.

8. Klag T, Stange EF, Wehkamp J. Defective antibacterial barrier in inflammatory bowel disease. Dig Dis. 2013;31: 310–316.

9. Gersemann M, Wehkamp J, Stange EF. Innate immune dysfunction in inflammatory bowel disease. J Intern Med. 2012;271: 421–428.

10. Buttó LF, Haller D. Dysbiosis in intestinal inflammation: Cause or consequence. Int J Med Microbiol. 2016; doi:10.1016/j.ijmm.2016.02.010

11. Candia E, Díaz-Jiménez D, Langjahr P, Núñez LE, de la Fuente M, Farfán N, et al. Increased production of soluble TLR2 by lamina propria mononuclear cells from ulcerative colitis patients. Immunobiology. 2012;217: 634–642.

12. Cantó E, Ricart E, Monfort D, González-Juan D, Balanzó J, Rodríguez-Sánchez JL, et al. TNFα production to TLR2 ligands in active IBD patients. Clin Immunol. 2006;119: 156–165.

13. Inohara N, Ogura Y, Fontalba A, Gutierrez O, Pons F, Crespo J, et al. Host recognition of bacterial muramyl dipeptide mediated through NOD2. Implications for Crohn's disease. J Biol Chem. 2003;278: 5509–5512.

14. Cario E. Barrier-protective function of intestinal epithelial Toll-like receptor 2. Mucosal Immunol. 2008;1 Suppl 1: S62–6.

15. Korn T, Bettelli E, Oukka M, Kuchroo VK. IL-17 and Th17 Cells. Annu Rev Immunol. Annual Reviews; 2009;27: 485–517.

16. Ranson N, Eri R. The Role of Inflammasomes in Intestinal Inflammation. American Journal of Medical and Biological Research. Science and Education Publishing; 2013;1: 64–76.

17. Fukata M, Abreu MT. What are toll-like receptors and what role may they have in IBD? Inflamm Bowel Dis. 2008;14: S90–S92.

18. Netea MG, Ferwerda G, de Jong DJ, Girardin SE, Kullberg BJ, van der Meer JWM. NOD2 3020insC mutation and the pathogenesis of Crohn’s disease: impaired IL-1beta production points to a loss-of-function phenotype. Neth J Med. 2005;63: 305–308.

19. Wehkamp J, Harder J, Weichenthal M, Schwab M, Schäffeler E, Schlee M, et al. NOD2 (CARD15) mutations in Crohn’s disease are associated with diminished mucosal alpha-defensin expression. Gut. 2004;53: 1658–1664.

20. Davis BK, Philipson C, Hontecillas R, Eden K, Bassaganya-Riera J, Allen IC. Emerging significance of NLRs in inflammatory bowel disease. Inflamm Bowel Dis. 2014;20: 2412–2432.

21. Aguilera M, Darby T, Melgar S. The complex role of inflammasomes in the pathogenesis of Inflammatory Bowel Diseases - lessons learned from experimental models. Cytokine Growth Factor Rev. 2014;25: 715–730.

22. Wang Q, Matsuo Y, Pradipta AR, Inohara N, Fujimoto Y, Fukase K. Synthesis of characteristic Mycobacterium peptidoglycan (PGN) fragments utilizing with chemoenzymatic preparation of meso-diaminopimelic acid (DAP), and their modulation of innate immune responses. Org Biomol Chem. 2016;14: 1013–1023.

23. Caruso R, Warner N, Inohara N, Núñez G. NOD1 and NOD2: Signaling, Host Defense, and Inflammatory Disease. Immunity. 2014;41: 898–908.

24. Davies JM, Abreu MT. The innate immune system and inflammatory bowel disease. Scand J Gastroenterol. 2015;50: 24–33.

25. Jostins L, Ripke S, Weersma RK, Duerr RH, McGovern DP, Hui KY, et al. Host-microbe interactions have shaped the genetic architecture of inflammatory bowel disease. Nature. 2012;491: 119–124.

26. Fukata M, Abreu MT. What are toll-like receptors and what role may they have in IBD? Inflamm Bowel Dis. 2008;14 Suppl 2: S90–2.

27. O’Neill LAJ, Golenbock D, Bowie AG. The history of Toll-like receptors [mdash] redefining innate immunity. Nat Rev Immunol. Nature Publishing Group; 2013;13: 453–460.

28. Brand S, Beigel F, Olszak T, Zitzmann K, Eichhorst ST, Otte J-M, et al. IL-22 is increased in active Crohn’s disease and promotes proinflammatory gene expression and intestinal epithelial cell migration. Am J Physiol Gastrointest Liver Physiol. 2006;290: G827–38.

29. Cildir G, Low KC, Tergaonkar V. Noncanonical NF-κB Signaling in Health and Disease. Trends Mol Med. 2016; doi:10.1016/j.molmed.2016.03.002

30. Sartor RB. Mechanisms of disease: pathogenesis of Crohn’s disease and ulcerative colitis. Nat Clin Pract Gastroenterol Hepatol. 2006;3: 390–407.

31. Marques R, Boneca IG. Expression and functional importance of innate immune receptors by intestinal epithelial cells. Cell Mol Life Sci. 2011;68: 3661–3673.

32. Lenardo MJ, Baltimore D. NF-kappa B: a pleiotropic mediator of inducible and tissue-specific gene control. Cell. 1989;58: 227–229.

33. Osborn L, Kunkel S, Nabel GJ. Tumor necrosis factor alpha and interleukin 1 stimulate the human immunodeficiency virus enhancer by activation of the nuclear factor kappa B. Proc Natl Acad Sci U S A.

1989;86: 2336–2340.

34. Donnelly RP, Crofford LJ, Freeman SL, Buras J, Remmers E, Wilder RL, et al. Tissue-specific regulation of IL-6 production by IL-4. Differential effects of IL-4 on nuclear factor-kappa B activity in monocytes and fibroblasts. J Immunol. 1993;151: 5603–5612.

35. de Waal Malefyt R, Abrams J, Bennett B, Figdor CG, de Vries JE. Interleukin 10(IL-10) inhibits cytokine synthesis by human monocytes: an autoregulatory role of IL-10 produced by monocytes. J Exp Med. 1991;174: 1209–1220.

36. Volpe E, Servant N, Zollinger R, Bogiatzi SI, Hupé P, Barillot E, et al. A critical function for transforming growth factor-β, interleukin 23 and proinflammatory cytokines in driving and modulating human TH-17 responses. Nat Immunol. Nature Publishing Group; 2008;9: 650–657.

37. Yamamoto M, Sato S, Hemmi H, Sanjo H, Uematsu S, Kaisho T, et al. Essential role for TIRAP in activation of the signalling cascade shared by TLR2 and TLR4. Nature. 2002;420: 324–329.

38. Hart AL, Al-Hassi HO, Rigby RJ, Bell SJ, Emmanuel AV, Knight SC, et al. Characteristics of intestinal dendritic cells in inflammatory bowel diseases. Gastroenterology. 2005;129: 50–65.

39. Acosta-Rodriguez EV, Napolitani G, Lanzavecchia A, Sallusto F. Interleukins 1β and 6 but not transforming growth factor-β are essential for the differentiation of interleukin 17–producing human T helper cells. Nat Immunol. Nature Publishing Group; 2007;8: 942–949.

40. Furfaro F, Gilardi D, Allocca M, Cicerone C, Correale C, Fiorino G, et al. IL-23 Blockade for Crohn s disease: next generation of anti-cytokine therapy. Expert Rev Clin Immunol. 2017; 1–11.

41. Cua DJ, Sherlock J, Chen Y, Murphy CA, Joyce B, Seymour B, et al. Interleukin-23 rather than interleukin-12 is the critical cytokine for autoimmune inflammation of the brain. Nature. 2003;421: 744–748.

42. McGovern D, Powrie F. The IL23 axis plays a key role in the pathogenesis of IBD. Gut. 2007;56: 1333–1336.

43. Iwakura Y, Ishigame H. The IL-23/IL-17 axis in inflammation. J Clin Invest. Am Soc Clin Investig; 2006;116: 1218–1222.

44. Sonnenberg GF, Fouser LA, Artis D. Border patrol: regulation of immunity, inflammation and tissue homeostasis at barrier surfaces by IL-22. Nat Immunol. 2011;12: 383–390.

45. Aujla SJ, Chan YR, Zheng M, Fei M, Askew DJ, Pociask DA, et al. IL-22 mediates mucosal host defense against Gram-negative bacterial pneumonia. Nat Med. 2008;14: 275–281.

46. Van Snick J. Interleukin-6: an overview. Annu Rev Immunol. 1990;8: 253–278.

47. Papadakis KA, Targan SR. Tumor necrosis factor: biology and therapeutic inhibitors. Gastroenterology. 2000;119: 1148–1157.

48. Carballo E, Lai WS, Blackshear PJ. Feedback inhibition of macrophage tumor necrosis factor-alpha production by tristetraprolin. Science. 1998;281: 1001–1005.

49. Cairns CB, Panacek EA, Harken AH, Banerjee A. Bench to Bedside Tumor Necrosis Factor-alpha: From Inflammation to Resuscitation. Acad Emerg Med. Wiley Online Library; 2000;7: 930–941.

50. Meldrum DR. Tumor necrosis factor in the heart. Am J Physiol. 1998;274: R577–95.

51. Shakhov AN, Collart MA, Vassalli P, Nedospasov SA, Jongeneel CV. Kappa B-type enhancers are involved in lipopolysaccharide-mediated transcriptional activation of the tumor necrosis factor alpha gene in primary macrophages. J Exp Med. 1990;171: 35–47.

52. Han J, Lee JD, Tobias PS, Ulevitch RJ. Endotoxin induces rapid protein tyrosine phosphorylation in 70Z/3 cells expressing CD14. J Biol Chem. 1993;268: 25009–25014.

53. Reimann T, Büscher D, Hipskind RA, Krautwald S, Lohmann-Matthes ML, Baccarini M. Lipopolysaccharide induces activation of the Raf-1/MAP kinase pathway. A putative role for Raf-1 in the induction of the IL-1 beta and the TNF-alpha genes. J Immunol. 1994;153: 5740–5749.

54. Oeckinghaus A, Ghosh S. The NF-kappaB family of transcription factors and its regulation. Cold Spring Harb Perspect Biol 1: a000034. 2009.

55. Dinarello CA. Immunological and inflammatory functions of the interleukin-1 family. Annu Rev Immunol. 2009;27: 519–550.

56. Lamkanfi M, Vande Walle L, Kanneganti T-D. Deregulated inflammasome signaling in disease. Immunol Rev. 2011;243: 163–173.

57. Shaw PJ, McDermott MF, Kanneganti T-D. Inflammasomes and autoimmunity. Trends Mol Med. 2011;17: 57–64.

58. Sim GC, Radvanyi L. The IL-2 cytokine family in cancer immunotherapy. Cytokine Growth Factor Rev. 2014;25: 377–390.

59. Hu X, Paik PK, Chen J, Yarilina A, Kockeritz L, Lu TT, et al. IFN-gamma suppresses IL-10 production and synergizes with TLR2 by regulating GSK3 and CREB/AP-1 proteins. Immunity. 2006;24: 563–574.

60. Jarry A, Bossard C, Bou-Hanna C, Masson D, Espaze E, Denis MG, et al. Mucosal IL-10 and TGF-beta play crucial roles in preventing LPS-driven, IFN-gamma-mediated epithelial damage in human colon explants. J Clin Invest. 2008;118: 1132–1142.

61. Zelová H, Hošek J. TNF-α signalling and inflammation: interactions between old acquaintances. Inflamm Res. 2013;62: 641–651.

62. Pariente B, Mocan I, Camus M, Dutertre C-A, Ettersperger J, Cattan P, et al. Activation of the receptor NKG2D leads to production of Th17 cytokines in CD4+ T cells of patients with Crohn's disease. Gastroenterology. 2011;141: 217–26, 226.e1–2.

63. Maasho K, Opoku-Anane J, Marusina AI, Coligan JE, Borrego F. Cutting Edge: NKG2D Is a Costimulatory Receptor for Human Naive CD8+ T Cells. The Journal of Immunology. 2005;174: 4480–4484.

64. Raulet DH. Roles of the NKG2D immunoreceptor and its ligands. Nat Rev Immunol. 2003;3: 781–790.

65. Groh V, Bruhl A, El-Gabalawy H, Nelson JL, Spies T. Stimulation of T cell autoreactivity by anomalous expression of NKG2D and its MIC ligands in rheumatoid arthritis. Proc Natl Acad Sci U S A. 2003;100: 9452–9457.

66. Vassalli P. The pathophysiology of tumor necrosis factors. Annu Rev Immunol. 1992;10: 411–452.

67. Economou JS, McBride WH, Essner R, Rhoades K, Golub S, Holmes EC, et al. Tumour necrosis factor production by IL-2-activated macrophages in vitro and in vivo. Immunology. 1989;67: 514–519.

68. Liu Z, Yadav PK, Xu X, Su J, Chen C, Tang M, et al. The increased expression of IL-23 in inflammatory bowel disease promotes intraepithelial and lamina propria lymphocyte inflammatory responses and cytotoxicity. J Leukoc Biol. 2011;89: 597–606.

69. Liu Z, Yadav PK, Xu X, Su J, Chen C, Tang M, et al. The increased expression of IL-23 in inflammatory bowel disease promotes intraepithelial and lamina propria lymphocyte inflammatory responses and cytotoxicity. J Leukoc Biol. 2011;89: 597–606.

70. Pedersen J, Coskun M, Soendergaard C, Salem M, Nielsen OH. Inflammatory pathways of importance for management of inflammatory bowel disease. World J Gastroenterol. 2014;20: 64–77.

71. Coskun M, Salem M, Pedersen J, Nielsen OH. Involvement of JAK/STAT signaling in the pathogenesis of inflammatory bowel disease. Pharmacol Res. 2013;76: 1–8.

72. Ma X. TNF-α and IL-12:a balancing act in macrophage functioning. Microbes Infect. 2001;3: 121–129.

73. Maloy KJ. The Interleukin-23 / Interleukin-17 axis in intestinal inflammation. J Intern Med. 2008;263: 584–590.

74. Uhlig HH, McKenzie BS, Hue S, Thompson C, Joyce-Shaikh B, Stepankova R, et al. Differential activity of IL-12 and IL-23 in mucosal and systemic innate immune pathology. Immunity. 2006;25: 309–318.

75. Langrish CL, McKenzie BS, Wilson NJ, de Waal Malefyt R, Kastelein RA, Cua DJ. IL-12 and IL-23: master regulators of innate and adaptive immunity. Immunol Rev. Wiley Online Library; 2004;202: 96–105.

76. Kikly K, Liu L, Na S, Sedgwick JD. The IL-23/Th17 axis: therapeutic targets for autoimmune inflammation. Curr Opin Immunol. 2006;18: 670–675.

77. Perussia B, Chan SH, D'Andrea A, Tsuji K, Santoli D, Pospisil M, et al. Natural killer (NK) cell stimulatory factor or IL-12 has differential effects on the proliferation of TCR-alpha beta+, TCR-gamma delta+ T lymphocytes, and NK cells. J Immunol. 1992;149: 3495–3502.

78. Carson WE, Giri JG, Lindemann MJ, Linett ML, Ahdieh M, Paxton R, et al. Interleukin (IL) 15 is a novel cytokine that activates human natural killer cells via components of the IL-2 receptor. J Exp Med. 1994;180: 1395–1403.

79. Fauriat C, Long EO, Ljunggren H-G, Bryceson YT. Regulation of human NK-cell cytokine and chemokine production by target cell recognition. Blood. 2010;115: 2167–2176.

80. Marcenaro E, Della Chiesa M, Bellora F, Parolini S, Millo R, Moretta L, et al. IL-12 or IL-4 prime human NK cells to mediate functionally divergent interactions with dendritic cells or tumors. J Immunol. 2005;174: 3992–3998.

81. Gurram B, Salzman NH, Kaldunski ML, Jia S, Li BUK, Stephens M, et al. Plasma-induced signatures reveal an extracellular milieu possessing an immunoregulatory bias in treatment-naive paediatric inflammatory bowel disease. Clin Exp Immunol. 2016;184: 36–49.

82. Yang L, Anderson DE, Baecher-Allan C, Hastings WD, Bettelli E, Oukka M, et al. IL-21 and TGF-&bgr; are required for differentiation of human TH17 cells. Nature. Nature Publishing Group; 2008;454: 350–352.

83. Pandiyan P, Zhu J. Origin and functions of pro-inflammatory cytokine producing Foxp3(+) regulatory T cells. Cytokine. 2015; doi:10.1016/j.cyto.2015.07.005

84. de Kivit S, Tobin MC, Forsyth CB, Keshavarzian A, Landay AL. Regulation of Intestinal Immune Responses

through TLR Activation: Implications for Pro- and Prebiotics. Front Immunol. 2014;5: 60.

85. Zanin-Zhorov A. Heat shock protein 60 enhances CD4+ CD25+ regulatory T cell function via innate TLR2 signaling. J Clin Invest. 2006;116: 2022–2032.

86. Gratchev A. TGF-β signalling in tumour associated macrophages. Immunobiology. 2016; doi:10.1016/j.imbio.2015.11.016

87. Smith PD, Smythies LE, Shen R, Greenwell-Wild T, Gliozzi M, Wahl SM. Intestinal macrophages and response to microbial encroachment. Mucosal Immunol. 2011;4: 31–42.

88. Assoian RK, Fleurdelys BE, Stevenson HC, Miller PJ, Madtes DK, Raines EW, et al. Expression and secretion of type beta transforming growth factor by activated human macrophages. Proc Natl Acad Sci U S A. 1987;84: 6020–6024.

89. Ungefroren H, Hyder A, Schulze M, Fawzy El-Sayed KM, Grage-Griebenow E, Nussler AK, et al. Peripheral Blood Monocytes as Adult Stem Cells: Molecular Characterization and Improvements in Culture Conditions to Enhance Stem Cell Features and Proliferative Potential. Stem Cells Int. 2016;2016: 7132751.

90. Liang L, Tan X, Zhou Q, Zhu Y, Tian Y, Yu H, et al. IL-1β triggered by peptidoglycan and lipopolysaccharide through TLR2/4 and ROS-NLRP3 inflammasome-dependent pathways is involved in ocular Behçet's disease. Invest Ophthalmol Vis Sci. 2013;54: 402–414.

91. Yang C-A, Chiang B-L. Inflammasomes and human autoimmunity: A comprehensive review. J Autoimmun. 2015;61: 1–8.

92. Beynon V, Quintana FJ, Weiner HL. Activated human CD4+CD45RO+ memory T-cells indirectly inhibit NLRP3 inflammasome activation through downregulation of P2X7R signalling. PLoS One. 2012;7: e39576.

93. Zenewicz LA, Antov A, Flavell RA. CD4 T-cell differentiation and inflammatory bowel disease. Trends Mol Med. 2009;15: 199–207.

94. Hedin CR, McCarthy NE, Louis P, Farquharson FM, McCartney S, Taylor K, et al. Altered intestinal microbiota and blood T cell phenotype are shared by patients with Crohn's disease and their unaffected siblings. Gut. 2014;63: 1578–1586.

95. Boyman O, Sprent J. The role of interleukin-2 during homeostasis and activation of the immune system. Nat Rev Immunol. Nature Publishing Group; 2012;12: 180–190.

96. Wilson NJ, Boniface K, Chan JR, McKenzie BS, Blumenschein WM, Mattson JD, et al. Development, cytokine profile and function of human interleukin 17–producing helper T cells. Nat Immunol. Nature Publishing Group; 2007;8: 950–957.

97. Oppmann B, Lesley R, Blom B, Timans JC, Xu Y, Hunte B, et al. Novel p19 protein engages IL-12p40 to form a cytokine, IL-23, with biological activities similar as well as distinct from IL-12. Immunity. 2000;13: 715–725.

98. Ashton-Rickardt PG, Opferman JT. Memory T lymphocytes. Cell Mol Life Sci. Birkhäuser Verlag; 56: 69–77.

99. Magalhaes JG, Fritz JH, Le Bourhis L, Sellge G, Travassos LH, Selvanantham T, et al. Nod2-dependent Th2 polarization of antigen-specific immunity. J Immunol. 2008;181: 7925–7935.

100. Lalor SJ, Dungan LS, Sutton CE, Basdeo SA, Fletcher JM, Mills KHG. Caspase-1–Processed Cytokines IL-1β and IL-18 Promote IL-17 Production by γδ and CD4 T Cells That Mediate Autoimmunity. The Journal of

Immunology. 2011;186: 5738–5748.

101. Nakanishi K, Yoshimoto T, Tsutsui H, Okamura H. Interleukin-18 is a unique cytokine that stimulates both Th1 and Th2 responses depending on its cytokine milieu. Cytokine Growth Factor Rev. 2001;12: 53–72.

102. Stoll S, Jonuleit H, Schmitt E, Müller G, Yamauchi H, Kurimoto M, et al. Production of functional IL-18 by different subtypes of murine and human dendritic cells (DC): DC-derived IL-18 enhances IL-12-dependent Th1 development. Eur J Immunol. 1998;28: 3231–3239.

103. Gerdes N, Sukhova GK, Libby P, Reynolds RS, Young JL, Schönbeck U. Expression of interleukin (IL)-18 and functional IL-18 receptor on human vascular endothelial cells, smooth muscle cells, and macrophages: implications for atherogenesis. J Exp Med. 2002;195: 245–257.

104. Dinarello CA. IL-18: A TH1 -inducing, proinflammatory cytokine and new member of the IL-1 family. J Allergy Clin Immunol. 1999;103: 11–24.

105. Dinarello CA. Immunological and inflammatory functions of the interleukin-1 family. Annu Rev Immunol. 2009;27: 519–550.

106. Hu M, Li W, Yan Q, Geng S, Pan Z, Cui G, et al. [Inflammasome responses in macrophages induced by C-terminal peptides of Escherichia coli EscI protein]. Wei Sheng Wu Xue Bao. 2015;55: 1350–1355.

107. D'Andrea A, Aste-Amezaga M, Valiante NM, Ma X, Kubin M, Trinchieri G. Interleukin 10 (IL-10) inhibits human lymphocyte interferon gamma-production by suppressing natural killer cell stimulatory factor/IL-12 synthesis in accessory cells. J Exp Med. 1993;178: 1041–1048.

108. Simon A, van der Meer JWM. Pathogenesis of familial periodic fever syndromes or hereditary autoinflammatory syndromes. Am J Physiol Regul Integr Comp Physiol. 2007;292: R86–98.

109. Rovedatti L, Kudo T, Biancheri P, Sarra M, Knowles CH, Rampton DS, et al. Differential regulation of interleukin 17 and interferon gamma production in inflammatory bowel disease. Gut. 2009;58: 1629–1636.

110. Parham C, Chirica M, Timans J, Vaisberg E, Travis M, Cheung J, et al. A Receptor for the Heterodimeric Cytokine IL-23 Is Composed of IL-12Rβ1 and a Novel Cytokine Receptor Subunit, IL-23R. The Journal of Immunology. 2002;168: 5699–5708.

111. Nakae S, Iwakura Y, Suto H, Galli SJ. Phenotypic differences between Th1 and Th17 cells and negative regulation of Th1 cell differentiation by IL-17. J Leukoc Biol. 2007;81: 1258–1268.

112. Silva MA, Menezes J, Deslandres C, Seidman EG. Anti-inflammatory role of interleukin-15 in Crohn's disease. Inflamm Bowel Dis. Wiley Online Library; 2005;11: 219–230.

113. Strober W, Fuss IJ. Proinflammatory cytokines in the pathogenesis of inflammatory bowel diseases. Gastroenterology. 2011;140: 1756–1767.

114. Barbulescu K, Becker C, Schlaak JF, Schmitt E, Meyer zum Büschenfelde K-H, Neurath MF. Cutting Edge: IL-12 and IL-18 Differentially Regulate the Transcriptional Activity of the Human IFN-γ Promoter in Primary CD4+ T Lymphocytes. The Journal of Immunology. 1998;160: 3642–3647.

115. Tominaga K, Yoshimoto T, Torigoe K, Kurimoto M, Matsui K, Hada T, et al. IL-12 synergizes with IL-18 or IL-1β for IFN-γ production from human T cells. Int Immunol. Jpn Soc Immunol; 2000;12: 151–160.

116. Gálvez J. Role of Th17 Cells in the Pathogenesis of Human IBD. ISRN Inflamm. 2014;2014: 928461.

117. Annunziato F, Cosmi L, Santarlasci V, Maggi L, Liotta F, Mazzinghi B, et al. Phenotypic and functional features of human Th17 cells. J Exp Med. 2007;204: 1849–1861.

118. Biedermann T, Röcken M, Carballido JM. TH1 and TH2 Lymphocyte Development and Regulation of TH Cell–Mediated Immune Responses of the Skin. J Investig Dermatol Symp Proc. Nature Publishing Group; 2004;9: 5–14.

119. Hovhannisyan Z, Treatman J, Littman DR, Mayer L. Characterization of Interleukin-17–Producing Regulatory T Cells in Inflamed Intestinal Mucosa From Patients With Inflammatory Bowel Diseases. Gastroenterology. 2011;140: 957–965.

120. Kubin M, Cassiano L, Chalupny J, Chin W, Cosman D, Fanslow W, et al. ULBP1, 2, 3: novel MHC class I-related molecules that bind to human cytomegalovirus glycoprotein UL16, activate NK cells. Eur J Immunol. 2001;31: 1428–1437.

121. Harris KM, Fasano A, Mann DL. Cutting edge: IL-1 controls the IL-23 response induced by gliadin, the etiologic agent in celiac disease. J Immunol. 2008;181: 4457–4460.

122. Ermann J, Staton T, Glickman JN, de Waal Malefyt R, Glimcher LH. Nod/Ripk2 signaling in dendritic cells activates IL-17A-secreting innate lymphoid cells and drives colitis in T-bet-/-.Rag2-/- (TRUC) mice. Proc Natl Acad Sci U S A. 2014;111: E2559–66.

123. Ermann J, Staton T, Glickman JN, de Waal Malefyt R, Glimcher LH. Nod/Ripk2 signaling in dendritic cells activates IL-17A--secreting innate lymphoid cells and drives colitis in T-bet-/-. Rag2-/-(TRUC) mice. Proceedings of the National Academy of Sciences. National Acad Sciences; 2014;111: E2559–E2566.

124. Ma X, Sun J, Papasavvas E, Riemann H, Robertson S, Marshall J, et al. Inhibition of IL-12 production in human monocyte-derived macrophages by TNF. J Immunol. 2000;164: 1722–1729.

125. Neurath MF. IL-23: a master regulator in Crohn disease. Nat Med. 2007;13: 26–28.

126. Brand S, Beigel F, Olszak T, Zitzmann K, Eichhorst ST, Otte J-M, et al. IL-22 is increased in active Crohn's disease and promotes proinflammatory gene expression and intestinal epithelial cell migration. Am J Physiol Gastrointest Liver Physiol. 2006;290: G827–38.

127. Aujla SJ, Kolls JK. IL-22: a critical mediator in mucosal host defense. J Mol Med . 2009;87: 451–454.

128. Cella M, Fuchs A, Vermi W, Facchetti F, Otero K, Lennerz JKM, et al. A human natural killer cell subset provides an innate source of IL-22 for mucosal immunity. Nature. 2009;457: 722–725.

129. Zenewicz LA, Flavell RA. Recent advances in IL-22 biology. Int Immunol. 2011;23: 159–163.

130. Zenewicz LA, Yancopoulos GD, Valenzuela DM, Murphy AJ, Stevens S, Flavell RA. Innate and adaptive interleukin-22 protects mice from inflammatory bowel disease. Immunity. 2008;29: 947–957.

131. Wolk K, Kunz S, Asadullah K, Sabat R. Cutting edge: immune cells as sources and targets of the IL-10 family members? J Immunol. 2002;168: 5397–5402.

132. Yang L, Anderson DE, Baecher-Allan C, Hastings WD, Bettelli E, Oukka M, et al. IL-21 and TGF-&bgr; are required for differentiation of human TH17 cells. Nature. Nature Publishing Group; 2008;454: 350–352.

133. Gharibi T, Majidi J, Kazemi T, Dehghanzadeh R, Motallebnezhad M, Babaloo Z. Biological effects of IL-21 on different immune cells and its role in autoimmune diseases. Immunobiology. 2016;221: 357–367.

134. Korn T, Bettelli E, Oukka M, Kuchroo VK. IL-17 and Th17 Cells. Annu Rev Immunol. Annual Reviews; 2009;27: 485–517.

135. Nurieva R, Yang XO, Martinez G, Zhang Y, Panopoulos AD, Ma L, et al. Essential autocrine regulation by IL-21 in the generation of inflammatory T cells. Nature. 2007;448: 480–483.

136. Korn T, Bettelli E, Gao W, Awasthi A, Jäger A, Strom TB, et al. IL-21 initiates an alternative pathway to induce proinflammatory TH17 cells. Nature. Nature Publishing Group; 2007;448: 484–487.

137. Zhou L, Ivanov II, Spolski R, Min R, Shenderov K, Egawa T, et al. IL-6 programs TH-17 cell differentiation by promoting sequential engagement of the IL-21 and IL-23 pathways. Nat Immunol. Nature Publishing Group; 2007;8: 967–974.

138. Wei L, Laurence A, Elias KM, O'Shea JJ. IL-21 is produced by Th17 cells and drives IL-17 production in a STAT3-dependent manner. J Biol Chem. ASBMB; 2007;282: 34605–34610.

139. Leonard WJ, Wan C-K. IL-21 Signaling in Immunity. F1000Res. 2016;5. doi:10.12688/f1000research.7634.1

140. Suto A, Kashiwakuma D, Kagami S-I, Hirose K, Watanabe N, Yokote K, et al. Development and characterization of IL-21-producing CD4+ T cells. J Exp Med. 2008;205: 1369–1379.

141. Dolff S, Bijl M, Huitema MG, Limburg PC, Kallenberg CGM, Abdulahad WH. Disturbed Th1, Th2, Th17 and Treg balance in patients with systemic lupus erythematosus. Clin Immunol. 2011;141: 197–204.

142. Zambrano-Zaragoza JF, Romo-Martínez EJ, Durán-Avelar M de J, García-Magallanes N, Vibanco-Pérez N. Th17 cells in autoimmune and infectious diseases. Int J Inflam. 2014;2014: 651503.

143. Miossec P, Kolls JK. Targeting IL-17 and TH17 cells in chronic inflammation. Nat Rev Drug Discov. 2012;11: 763–776.

144. Jin W, Dong C. IL-17 cytokines in immunity and inflammation. Emerg Microbes Infect. Nature Publishing Group; 2013;2: e60.

145. Reynolds JM, Angkasekwinai P, Dong C. IL-17 family member cytokines: regulation and function in innate immunity. Cytokine Growth Factor Rev. 2010;21: 413–423.

146. Newcomb DC, Boswell MG, Zhou W, Huckabee MM, Goleniewska K, Sevin CM, et al. Human TH17 cells express a functional IL-13 receptor and IL-13 attenuates IL-17A production. J Allergy Clin Immunol. 2011;127: 1006–13.e1–4.

147. Marlow GJ. Why interleukin-10 supplementation does not work in Crohn's disease patients. World J Gastroenterol. 2013;19: 3931.

148. Bai AHC, Wu WKK, Xu L, Wong SH, Go MY, Chan AWH, et al. Dysregulated Lysine Acetyltransferase 2B Promotes Inflammatory Bowel Disease Pathogenesis Through Transcriptional Repression of Interleukin-10. J Crohns Colitis. 2016;10: 726–734.

149. Hu X, Paik PK, Chen J, Yarilina A, Kockeritz L, Lu TT, et al. IFN-gamma suppresses IL-10 production and synergizes with TLR2 by regulating GSK3 and CREB/AP-1 proteins. Immunity. 2006;24: 563–574.

150. Geremia A, Biancheri P, Allan P, Corazza GR, Di Sabatino A. Innate and adaptive immunity in inflammatory bowel disease. Autoimmun Rev. 2014;13: 3–10.

151. Valencia X, Stephens G, Goldbach-Mansky R, Wilson M, Shevach EM, Lipsky PE. TNF downmodulates the function of human CD4+ CD25hi T-regulatory cells. Blood. Am Soc Hematology; 2006;108: 253–261.

152. Liu Z, Feng B-S, Yang S-B, Chen X, Su J, Yang P-C. Interleukin (IL)-23 suppresses IL-10 in inflammatory bowel disease. J Biol Chem. 2012;287: 3591–3597.

153. McGeachy MJ, Bak-Jensen KS, Chen YI, Tato CM, Blumenschein W, McClanahan T, et al. TGF-β and IL-6 drive the production of IL-17 and IL-10 by T cells and restrain TH-17 cell--mediated pathology. Nat Immunol. Nature Publishing Group; 2007;8: 1390–1397.

154. Tsuji-Takayama K, Suzuki M, Yamamoto M, Harashima A, Okochi A, Otani T, et al. The production of IL-10 by human regulatory T cells is enhanced by IL-2 through a STAT5-responsive intronic enhancer in the IL-10 locus. J Immunol. 2008;181: 3897–3905.

155. Bros M, Montermann E, Cholaszczyńska A, Reske-Kunz AB. The phosphodiesterase 4 inhibitor roflumilast augments the Th17-promoting capability of dendritic cells by enhancing IL-23 production, and impairs their T cell stimulatory activity due to elevated IL-10. Int Immunopharmacol. 2016;35: 174–184.

156. Li J, Ueno A, Fort Gasia M, Luider J, Wang T, Hirota C, et al. Profiles of Lamina Propria T Helper Cell Subsets Discriminate Between Ulcerative Colitis and Crohn's Disease. Inflamm Bowel Dis. 2016; 1.

157. Calderón-Gómez E, Bassolas-Molina H, Mora-Buch R, Dotti I, Planell N, Esteller M, et al. Commensal-specific CD4+ Cells From Patients With Crohn's Disease Have a T-helper 17 Inflammatory Profile. Gastroenterology. 2016; doi:10.1053/j.gastro.2016.05.050

158. Laurence A, O'Shea JJ. TH-17 differentiation: of mice and men. Nat Immunol. Nature Publishing Group; 2007;8: 903–905.

159. Park H, Li Z, Yang XO, Chang SH, Nurieva R, Wang Y-H, et al. A distinct lineage of CD4 T cells regulates tissue inflammation by producing interleukin 17. Nat Immunol. 2005;6: 1133–1141.

160. Harrington LE, Hatton RD, Mangan PR, Turner H, Murphy TL, Murphy KM, et al. Interleukin 17–producing CD4+ effector T cells develop via a lineage distinct from the T helper type 1 and 2 lineages. Nat Immunol. Nature Publishing Group; 2005;6: 1123–1132.

161. Hoeve MA, Savage NDL, de Boer T, Langenberg DML, de Waal Malefyt R, Ottenhoff THM, et al. Divergent effects of IL-12 and IL-23 on the production of IL-17 by human T cells. Eur J Immunol. 2006;36: 661–670.

162. Kimura A, Kishimoto T. IL-6: regulator of Treg/Th17 balance. Eur J Immunol. 2010;40: 1830–1835.

163. Wilson NJ, Boniface K, Chan JR, McKenzie BS, Blumenschein WM, Mattson JD, et al. Development, cytokine profile and function of human interleukin 17-producing helper T cells. Nat Immunol. 2007;8: 950–957.

164. Zhou L, Littman DR. Transcriptional regulatory networks in Th17 cell differentiation. Curr Opin Immunol. 2009;21: 146–152.

165. Aggarwal S, Ghilardi N, Xie M-H, de Sauvage FJ, Gurney AL. Interleukin-23 promotes a distinct CD4 T cell activation state characterized by the production of interleukin-17. J Biol Chem. 2003;278: 1910–1914.

166. Langrish CL, Chen Y, Blumenschein WM, Mattson J, Basham B, Sedgwick JD, et al. IL-23 drives a pathogenic T cell population that induces autoimmune inflammation. J Exp Med. 2005;201: 233–240.

167. Tesmer LA, Lundy SK, Sarkar S, Fox DA. Th17 cells in human disease. Immunol Rev. 2008;223: 87–113.

168. Annunziato F, Cosmi L, Santarlasci V, Maggi L, Liotta F, Mazzinghi B, et al. Phenotypic and functional features of human Th17 cells. J Exp Med. 2007;204: 1849–1861.

169. Wilson NJ, Boniface K, Chan JR, McKenzie BS, Blumenschein WM, Mattson JD, et al. Development, cytokine profile and function of human interleukin 17-producing helper T cells. Nat Immunol. 2007;8: 950–957.

170. Amadi-Obi A, Yu C-R, Liu X, Mahdi RM, Clarke GL, Nussenblatt RB, et al. TH17 cells contribute to uveitis and scleritis and are expanded by IL-2 and inhibited by IL-27/STAT1. Nat Med. Nature Publishing Group; 2007;13: 711–718.

171. Liu Z, Geboes K, Heremans H, Overbergh L, Mathieu C, Rutgeerts P, et al. Role of interleukin-12 in the induction of mucosal inflammation and abrogation of regulatory T cell function in chronic experimental colitis. Eur J Immunol. 2001;31: 1550–1560.

172. Collier SP, Henderson MA, Tossberg JT, Aune TM. Regulation of the Th1 genomic locus from Ifng through Tmevpg1 by T-bet. J Immunol. 2014;193: 3959–3965.

173. Zygmunt B, Veldhoen M. T helper cell differentiation more than just cytokines. Adv Immunol. 2011;109: 159–196.

174. Chen M, Wang H, Chen W, Meng G. Regulation of adaptive immunity by the NLRP3 inflammasome. Int Immunopharmacol. 2011;11: 549–554.

175. Yoshimoto T, Takeda K, Tanaka T, Ohkusu K, Kashiwamura S-I, Okamura H, et al. IL-12 up-regulates IL-18 receptor expression on T cells, Th1 cells, and B cells: synergism with IL-18 for IFN-γ production. The Journal of Immunology. Am Assoc Immnol; 1998;161: 3400–3407.

176. Peng H, Wang W, Zhou M, Li R, Pan H-F, Ye D-Q. Role of interleukin-10 and interleukin-10 receptor in systemic lupus erythematosus. Clin Rheumatol. 2013;32: 1255–1266.

177. Moore KW, de Waal Malefyt R, Coffman RL, O'Garra A. Interleukin-10 and the interleukin-10 receptor. Annu Rev Immunol. Annual Reviews 4139 El Camino Way, PO Box 10139, Palo Alto, CA 94303-0139, USA; 2001;19: 683–765.

178. O'Connor W Jr, Kamanaka M, Booth CJ, Town T, Nakae S, Iwakura Y, et al. A protective function for interleukin 17A in T cell–mediated intestinal inflammation. Nat Immunol. Nature Publishing Group; 2009;10: 603–609.

179. Wan YY, Flavell RA. "Yin-Yang" functions of transforming growth factor-beta and T regulatory cells in immune regulation. Immunol Rev. 2007;220: 199–213.

180. Plata-Salamán CR, Ilyin SE, Gayle D, Flynn MC. Gram-negative and gram-positive bacterial products induce differential cytokine profiles in the brain: analysis using an integrative molecular-behavioral in vivo model. Int J Mol Med. 1998;1: 387–397.

181. Smits HH, van Beelen AJ, Hessle C, Westland R, de Jong E, Soeteman E, et al. Commensal Gram-negative bacteria prime human dendritic cells for enhanced IL-23 and IL-27 expression and enhanced Th1 development. Eur J Immunol. 2004;34: 1371–1380.

182. Sartor RB. Mechanisms of disease: pathogenesis of Crohn's disease and ulcerative colitis. Nat Clin Pract Gastroenterol Hepatol. 2006;3: 390–407.

183. de Sa Pinheiro AA, Morrot A, Chakravarty S, Overstreet M, Bream JH, Irusta PM, et al. IL-4 induces a wide-spectrum intracellular signaling cascade in CD8+ T cells. J Leukoc Biol. Soc Leukocyte Biology; 2007;81: 1102–

1110.

184. Nelms K, Keegan AD, Zamorano J, Ryan JJ, Paul WE. The IL-4 receptor: signaling mechanisms and biologic functions. Annu Rev Immunol. 1999;17: 701–738.

185. Cuneo AA, Autieri MV. Expression and function of anti-inflammatory interleukins: the other side of the vascular response to injury. Curr Vasc Pharmacol. 2009;7: 267–276.

186. Paul WE. Interleukin-4: a prototypic immunoregulatory lymphokine. Blood. 1991;77: 1859–1870.

187. Gallagher G, Eskdale J, Jordan W, Peat J, Campbell J, Boniotto M, et al. Human interleukin-19 and its receptor: a potential role in the induction of Th2 responses. Int Immunopharmacol. 2004;4: 615–626.

188. Takeda K, Tanaka T, Shi W, Matsumoto M, Minami M, Kashiwamura S, et al. Essential role of Stat6 in IL-4 signalling. Nature. 1996;380: 627–630.

189. Ho IC, Hodge MR, Rooney JW, Glimcher LH. The proto-oncogene c-maf is responsible for tissue-specific expression of interleukin-4. Cell. 1996;85: 973–983.

190. Zheng W, Flavell RA. The transcription factor GATA-3 is necessary and sufficient for Th2 cytokine gene expression in CD4 T cells. Cell. 1997;89: 587–596.

191. Gorelik L, Flavell RA. Transforming growth factor-β in T-cell biology. Nat Rev Immunol. Nature Publishing Group; 2002;2: 46–53.

192. Gorelik L, Flavell RA. Abrogation of TGFβ Signaling in T Cells Leads to Spontaneous T Cell Differentiation and Autoimmune Disease. Immunity. 2000;12: 171–181.

193. Pulendran B, Kumar P, Cutler CW, Mohamadzadeh M, Van Dyke T, Banchereau J. Lipopolysaccharides from distinct pathogens induce different classes of immune responses in vivo. J Immunol. 2001;167: 5067–5076.

194. Dillon S, Agrawal A, Van Dyke T, Landreth G, McCauley L, Koh A, et al. A Toll-like receptor 2 ligand stimulates Th2 responses in vivo, via induction of extracellular signal-regulated kinase mitogen-activated protein kinase and c-Fos in dendritic cells. J Immunol. 2004;172: 4733–4743.

195. Agrawal S, Agrawal A, Doughty B, Gerwitz A, Blenis J, Van Dyke T, et al. Cutting edge: different Toll-like receptor agonists instruct dendritic cells to induce distinct Th responses via differential modulation of extracellular signal-regulated kinase-mitogen-activated protein kinase and c-Fos. The Journal of Immunology. Am Assoc Immnol; 2003;171: 4984–4989.

196. Redecke V, Häcker H, Datta SK, Fermin A, Pitha PM, Broide DH, et al. Cutting edge: activation of Toll-like receptor 2 induces a Th2 immune response and promotes experimental asthma. J Immunol. 2004;172: 2739–2743.

197. Elenkov IJ, Webster E, Papanicolaou DA, Fleisher TA, Chrousos GP, Wilder RL. Histamine potently suppresses human IL-12 and stimulates IL-10 production via H2 receptors. J Immunol. 1998;161: 2586–2593.

198. Yamane H, Zhu J, Paul WE. Independent roles for IL-2 and GATA-3 in stimulating naive CD4+ T cells to generate a Th2-inducing cytokine environment. J Exp Med. 2005;202: 793–804.

199. Sad S, Mosmann TR. Single IL-2-secreting precursor CD4 T cell can develop into either Th1 or Th2 cytokine secretion phenotype. J Immunol. 1994;153: 3514–3522.

200. Hong J, Leung E, Fraser AG, Merriman TR, Vishnu P, Krissansen GW. IL4, IL10, IL16, and TNF polymorphisms in New Zealand Caucasian Crohn's disease patients. Int J Colorectal Dis. 2008;23: 335–337.

201. Amsen D, Spilianakis CG, Flavell RA. How are TH1 and TH2 effector cells made? Curr Opin Immunol. 2009;21: 153–160.

202. Ansel KM, Djuretic I, Tanasa B, Rao A. Regulation of Th2 differentiation and Il4 locus accessibility. Annu Rev Immunol. 2006;24: 607–656.

203. Jankovic D, Kullberg MC, Noben-Trauth N, Caspar P, Paul WE, Sher A. Single cell analysis reveals that IL-4 receptor/Stat6 signaling is not required for the in vivo or in vitro development of CD4+ lymphocytes with a Th2 cytokine profile. J Immunol. 2000;164: 3047–3055.

204. Paul WE, Zhu J. How are TH2-type immune responses initiated and amplified? Nat Rev Immunol. Nature Publishing Group; 2010;10: 225–235.

205. Koller FL, Hwang DG, Dozier EA, Fingleton B. Epithelial interleukin-4 receptor expression promotes colon tumor growth. Carcinogenesis. 2010;31: 1010–1017.

206. Kelly-Welch AE, Hanson EM, Boothby MR, Keegan AD. Interleukin-4 and interleukin-13 signaling connections maps. Science. 2003;300: 1527–1528.

207. Bettelli E, Carrier Y, Gao W, Korn T, Strom TB, Oukka M, et al. Reciprocal developmental pathways for the generation of pathogenic effector TH17 and regulatory T cells. Nature. 2006;441: 235–238.

208. Yadav V, Varum F, Bravo R, Furrer E, Bojic D, Basit AW. Inflammatory bowel disease: exploring gut pathophysiology for novel therapeutic targets. Transl Res. 2016; doi:10.1016/j.trsl.2016.04.009

209. DePaolo RW, Abadie V, Tang F, Fehlner-Peach H, Hall JA, Wang W, et al. Co-adjuvant effects of retinoic acid and IL-15 induce inflammatory immunity to dietary antigens. Nature. 2011;471: 220–224.

210. Liu Z, Geboes K, Colpaert S, D'Haens GR, Rutgeerts P, Ceuppens JL. IL-15 is highly expressed in inflammatory bowel disease and regulates local T cell-dependent cytokine production. J Immunol. 2000;164: 3608–3615.

211. Tagaya Y, Bamford RN, DeFilippis AP, Waldmann TA. IL-15: a pleiotropic cytokine with diverse receptor/signaling pathways whose expression is controlled at multiple levels. Immunity. 1996;4: 329–336.

212. Yang X-K, Xu W-D, Leng R-X, Liang Y, Liu Y-Y, Fang X-Y, et al. Therapeutic potential of IL-15 in rheumatoid arthritis. Hum Immunol. 2015; doi:10.1016/j.humimm.2015.09.041

213. Klimiuk PA, Yang H, Goronzy JJ, Weyand CM. Production of cytokines and metalloproteinases in rheumatoid synovitis is T cell dependent. Clin Immunol. 1999;90: 65–78.

214. Badolato R, Ponzi AN, Millesimo M, Notarangelo LD, Musso T. Interleukin-15 (IL-15) induces IL-8 and monocyte chemotactic protein 1 production in human monocytes. Blood. 1997;90: 2804–2809.

215. Ma X, Sun J, Papasavvas E, Riemann H, Robertson S, Marshall J, et al. Inhibition of IL-12 production in human monocyte-derived macrophages by TNF. J Immunol. 2000;164: 1722–1729.

216. Ma X, Yan W, Zheng H, Du Q, Zhang L, Ban Y, et al. Regulation of IL-10 and IL-12 production and function in macrophages and dendritic cells. F1000Res. 2015;4. doi:10.12688/f1000research.7010.1

217. Re F, Strominger JL. IL-10 released by concomitant TLR2 stimulation blocks the induction of a subset of Th1

cytokines that are specifically induced by TLR4 or TLR3 in human dendritic cells. J Immunol. 2004;173: 7548–7555.

218. D'Andrea A, Ma X, Aste-Amezaga M, Paganin C, Trinchieri G. Stimulatory and inhibitory effects of interleukin (IL)-4 and IL-13 on the production of cytokines by human peripheral blood mononuclear cells: priming for IL-12 and tumor necrosis factor alpha production. J Exp Med. Rockefeller University Press; 1995;181: 537–546.

219. Aksoy E, Amraoui Z, Goriely S, Goldman M, Willems F. Critical role of protein kinase C ϵ for lipopolysaccharide-induced IL-12 synthesis in monocyte-derived dendritic cells. Eur J Immunol. Wiley Online Library; 2002;32: 3040–3049.

220. Wesa AK, Galy A. IL-1 beta induces dendritic cells to produce IL-12. Int Immunol. 2001;13: 1053–1061.

221. Becker C, Wirtz S, Ma X, Blessing M, Galle PR, Neurath MF. Regulation of IL-12 p40 promoter activity in primary human monocytes: roles of NF-κB, CCAAT/enhancer-binding protein β, and PU. 1 and identification of a novel repressor element (GA-12) that responds to IL-4 and prostaglandin E2. The Journal of Immunology. Am Assoc Immnol; 2001;167: 2608–2618.

222. Murphy TL, Cleveland MG, Kulesza P, Magram J, Murphy KM. Regulation of interleukin 12 p40 expression through an NF-kappa B half-site. Mol Cell Biol. 1995;15: 5258–5267.

223. Verreck FAW, de Boer T, Langenberg DML, Hoeve MA, Kramer M, Vaisberg E, et al. Human IL-23-producing type 1 macrophages promote but IL-10-producing type 2 macrophages subvert immunity to (myco) bacteria. Proc Natl Acad Sci U S A. National Acad Sciences; 2004;101: 4560–4565.

224. Ma X, Chow JM, Gri G, Carra G, Gerosa F, Wolf SF, et al. The interleukin 12 p40 gene promoter is primed by interferon gamma in monocytic cells. J Exp Med. 1996;183: 147–157.

225. Belladonna ML, Renauld J-C, Bianchi R, Vacca C, Fallarino F, Orabona C, et al. IL-23 and IL-12 have overlapping, but distinct, effects on murine dendritic cells. J Immunol. 2002;168: 5448–5454.

226. Seyfizadeh N, Seyfizadeh N, Gharibi T, Babaloo Z. Interleukin-13 as an important cytokine: A review on its roles in some human diseases. Acta Microbiol Immunol Hung. 2015;62: 341–378.

227. Rengarajan J, Szabo SJ, Glimcher LH. Transcriptional regulation of Th1/Th2 polarization. Immunol Today. 2000;21: 479–483.

228. Minty A, Chalon P, Derocq J-M, Dumont X, Guillemot J-C, Kaghad M, et al. lnterleukin-13 is a new human lymphokine regulating inflammatory and immune responses. Nature Publishing Group; 1993;362: 248–250.

229. Newcomb DC, Boswell MG, Huckabee MM, Goleniewska K, Dulek DE, Reiss S, et al. IL-13 regulates Th17 secretion of IL-17A in an IL-10-dependent manner. J Immunol. 2012;188: 1027–1035.

230. Mannon PJ, Hornung RL, Yang Z, Yi C, Groden C, Friend J, et al. Suppression of inflammation in ulcerative colitis by interferon-β-1a is accompanied by inhibition of IL-13 production. Gut. 2011;60: 449–455.

231. Maul J, Loddenkemper C, Mundt P, Berg E, Giese T, Stallmach A, et al. Peripheral and intestinal regulatory CD4+ CD25(high) T cells in inflammatory bowel disease. Gastroenterology. 2005;128: 1868–1878.

232. Yang XO, Pappu BP, Nurieva R, Akimzhanov A, Kang HS, Chung Y, et al. TH17 Lineage differentiation is programmed by orphan nuclear receptors ROR and ROR. Immunity. 2008;28: 2939.

233. Chamouard P, Monneaux F, Richert Z, Voegeli A-C, Lavaux T, Gaub MP, et al. Diminution of Circulating CD4+

CD25high T Cells in Naïve Crohn's Disease. Dig Dis Sci. Springer; 2009;54: 2084–2093.

234. Mattei F, Schiavoni G, Belardelli F, Tough DF. IL-15 is expressed by dendritic cells in response to type I IFN, double-stranded RNA, or lipopolysaccharide and promotes dendritic cell activation. J Immunol. 2001;167: 1179–1187.

235. González-Álvaro I, Ortiz AM, Alvaro-Gracia JM, Castañeda S, Díaz-Sánchez B, Carvajal I, et al. Interleukin 15 levels in serum may predict a severe disease course in patients with early arthritis. PLoS One. 2011;6: e29492.

236. Issekutz AC, Derfalvi B, Käsermann F, Rowter D. Potentiation of cytokine-induced proliferation of human Natural Killer cells by intravenous immunoglobulin G. Clin Immunol. 2015;161: 373–383.

237. Li Y, Meng F-D, Tian X, Sui C-G, Liu Y-P, Jiang Y-H. Impact of IL-2 and IL-2R SNPs on proliferation and tumor-killing activity of lymphokine-activated killer cells from healthy chinese blood donors. Asian Pac J Cancer Prev. 2014;15: 7965–7970.

238. Waldmann TA. The biology of interleukin-2 and interleukin-15: implications for cancer therapy and vaccine design. Nat Rev Immunol. 2006;6: 595–601.

239. Carson WE, Fehniger TA, Haldar S, Eckhert K, Lindemann MJ, Lai CF, et al. A potential role for interleukin-15 in the regulation of human natural killer cell survival. J Clin Invest. 1997;99: 937–943.

240. Cai G, Kastelein RA, Hunter CA. IL-10 enhances NK cell proliferation, cytotoxicity and production of IFN-gamma when combined with IL-18. Eur J Immunol. 1999;29: 2658–2665.

241. Mackall CL, Fry TJ, Gress RE. Harnessing the biology of IL-7 for therapeutic application. Nat Rev Immunol. 2011;11: 330–342.

242. Fewkes NM, Mackall CL. Novel gamma-chain cytokines as candidate immune modulators in immune therapies for cancer. Cancer J. 2010;16: 392–398.

243. Fehniger TA, Cooper MA, Nuovo GJ, Cella M, Facchetti F, Colonna M, et al. CD56bright natural killer cells are present in human lymph nodes and are activated by T cell--derived IL-2: a potential new link between adaptive and innate immunity. Blood. Am Soc Hematology; 2003;101: 3052–3057.

244. Benson JM, Sachs CW, Treacy G, Zhou H, Pendley CE, Brodmerkel CM, et al. Therapeutic targeting of the IL-12/23 pathways: generation and characterization of ustekinumab. Nat Biotechnol. Nature Research; 2011;29: 615–624.

245. Trinchieri G. Interleukin-12 and the regulation of innate resistance and adaptive immunity. Nat Rev Immunol. 2003;3: 133–146.

246. Trinchieri G. Interleukin-12: a cytokine produced by antigen-presenting cells with immunoregulatory functions in the generation of T-helper cells type 1 and cytotoxic lymphocytes. Blood. 1994;84: 4008–4027.

247. Murphy K, Travers P, Walport M. Janeway's Immunobiology. 2008. p. 887.

248. Biswas A, Petnicki-Ocwieja T, Kobayashi KS. Nod2: a key regulator linking microbiota to intestinal mucosal immunity. J Mol Med . 2012;90: 15–24.

249. Ramasundara M, Leach ST, Lemberg DA, Day AS. Defensins and inflammation: the role of defensins in inflammatory bowel disease. J Gastroenterol Hepatol. 2009;24: 202–208.

250. Keshav S. Paneth cells: leukocyte-like mediators of innate immunity in the intestine. J Leukoc Biol. 2006;80: 500–508.

251. Voss E, Wehkamp J, Wehkamp K, Stange EF, Schröder JM, Harder J. NOD2/CARD15 mediates induction of the antimicrobial peptide human beta-defensin-2. J Biol Chem. 2006;281: 2005–2011.

252. Katz LH, Kopylov U, Fudim E, Yavzori M, Picard O, Ungar B, et al. Expression of IL-2, IL-17 and TNF-alpha in patients with Crohn's disease treated with anti-TNF antibodies. Clin Res Hepatol Gastroenterol. 2014;38: 491–498.

253. Smith KA. Interleukin-2: inception, impact, and implications. Science. 1988;240: 1169–1176.

254. Ponce LF, García-Martínez K, León K. Quantitative Contribution of IL2Rγ to the Dynamic Formation of IL2-IL2R Complexes. PLoS One. 2016;11: e0155684.

255. Setoguchi R, Hori S, Takahashi T, Sakaguchi S. Homeostatic maintenance of natural Foxp3(+) CD25(+) CD4(+) regulatory T cells by interleukin (IL)-2 and induction of autoimmune disease by IL-2 neutralization. J Exp Med. 2005;201: 723–735.

256. Malek TR. The biology of interleukin-2. Annu Rev Immunol. Annual Reviews; 2008;26: 453–479.

257. Granucci F, Vizzardelli C, Pavelka N, Feau S, Persico M, Virzi E, et al. Inducible IL-2 production by dendritic cells revealed by global gene expression analysis. Nat Immunol. 2001;2: 882–888.

258. León F, Roldán E, Sanchez L, Camarero C, Bootello A, Roy G. Human small-intestinal epithelium contains functional natural killer lymphocytes. Gastroenterology. 2003;125: 345–356.

259. Farber DL, Yudanin NA, Restifo NP. Human memory T cells: generation, compartmentalization and homeostasis. Nat Rev Immunol. 2014;14: 24–35.

260. Mann ER, Li X. Intestinal antigen-presenting cells in mucosal immune homeostasis: crosstalk between dendritic cells, macrophages and B-cells. World J Gastroenterol. 2014;20: 9653–9664.

261. Sharif O, Bolshakov VN, Raines S, Newham P, Perkins ND. Transcriptional profiling of the LPS induced NF-kappaB response in macrophages. BMC Immunol. 2007;8: 1.

262. Chanput W, Mes J, Vreeburg RAM, Savelkoul HFJ, Wichers HJ. Transcription profiles of LPS-stimulated THP-1 monocytes and macrophages: a tool to study inflammation modulating effects of food-derived compounds. Food Funct. 2010;1: 254–261.

263. Caldwell AB, Cheng Z, Vargas JD, Birnbaum HA, Hoffmann A. Network dynamics determine the autocrine and paracrine signaling functions of TNF. Genes Dev. 2014;28: 2120–2133.

264. Cooney R, Baker J, Brain O, Danis B, Pichulik T, Allan P, et al. NOD2 stimulation induces autophagy in dendritic cells influencing bacterial handling and antigen presentation. Nat Med. 2010;16: 90–97.

265. Ade N, Antonios D, Kerdine-Romer S, Boisleve F, Rousset F, Pallardy M. NF-κB Plays a Major Role in the Maturation of Human Dendritic Cells Induced by NiSO4 but not by DNCB. Toxicol Sci. 2007;99: 488–501.

266. Kaisho T, Tanaka T. Turning NF-κB and IRFs on and off in DC. Trends Immunol. Elsevier; 2008;29: 329–336.

267. Hernandez A, Burger M, Blomberg BB, Ross WA, Gaynor JJ, Lindner I, et al. Inhibition of NF-κB during human dendritic cell differentiation generates anergy and regulatory T-cell activity for one but not two human

leukocyte antigen DR mismatches. Hum Immunol. 2007/9;68: 715–729.

268. Creusot RJ. NF-κB in DCs: it takes effort to be immature. Nat Med. 2011;17: 1554–1556.

269. Tassi I, Claudio E, Wang H, Tang W, Ha H-L, Saret S, et al. The NF-κB regulator Bcl-3 governs dendritic cell antigen presentation functions in adaptive immunity. J Immunol. 2014;193: 4303–4311.

270. Carapito R, Bahram S. Genetics, genomics, and evolutionary biology of NKG2D ligands. Immunol Rev. 2015;267: 88–116.

271. Friese MA, Wischhusen J, Wick W, Weiler M, Eisele G, Steinle A, et al. RNA interference targeting transforming growth factor-beta enhances NKG2D-mediated antiglioma immune response, inhibits glioma cell migration and invasiveness, and abrogates tumorigenicity in vivo. Cancer Res. 2004;64: 7596–7603.

272. López-Hernández R, Valdés M, Lucas D, Campillo JA, Martínez-Garcia P, Salama H, et al. Association analysis of MICA gene polymorphism and MICA-129 dimorphism with inflammatory bowel disease susceptibility in a Spanish population. Hum Immunol. 2010;71: 512–514.

273. Gannagé M, Buzyn A, Bogiatzi SI, Lambert M, Soumelis V, Dal Cortivo L, et al. Induction of NKG2D ligands by gamma radiation and tumor necrosis factor-alpha may participate in the tissue damage during acute graft-versus-host disease. Transplantation. 2008;85: 911–915.

274. Allez M, Tieng V, Nakazawa A, Treton X, Pacault V, Dulphy N, et al. CD4+NKG2D+ T cells in Crohn's disease mediate inflammatory and cytotoxic responses through MICA interactions. Gastroenterology. 2007;132: 2346–2358.

275. Burgess SJ, Maasho K, Masilamani M, Narayanan S, Borrego F, Coligan JE. The NKG2D receptor: immunobiology and clinical implications. Immunol Res. 2008;40: 18–34.

276. Burgess SJ, Marusina AI, Pathmanathan I, Borrego F, Coligan JE. IL-21 down-regulates NKG2D/DAP10 expression on human NK and CD8+ T cells. J Immunol. 2006;176: 1490–1497.

277. Castriconi R, Cantoni C, Della Chiesa M, Vitale M, Marcenaro E, Conte R, et al. Transforming growth factor beta 1 inhibits expression of NKp30 and NKG2D receptors: consequences for the NK-mediated killing of dendritic cells. Proc Natl Acad Sci U S A. 2003;100: 4120–4125.

278. Song H, Hur DY, Kim K-E, Park H, Kim T, Kim C-W, et al. IL-2/IL-18 prevent the down-modulation of NKG2D by TGF-β in NK cells via the c-Jun N-terminal kinase (JNK) pathway. Cell Immunol. Elsevier; 2006;242: 39–45.

279. Groh V, Wu J, Yee C, Spies T. Tumour-derived soluble MIC ligands impair expression of NKG2D and T-cell activation. Nature. 2002;419: 734–738.

280. Spear P, Wu M-R, Sentman M-L, Sentman CL. NKG2D ligands as therapeutic targets. Cancer Immun. 2013;13: 8.

281. Garrity D, Call ME, Feng J, Wucherpfennig KW. The activating NKG2D receptor assembles in the membrane with two signaling dimers into a hexameric structure. Proc Natl Acad Sci U S A. 2005;102: 7641–7646.

282. Burgess SJ, Maasho K, Masilamani M, Narayanan S, Borrego F, Coligan JE. The NKG2D receptor: immunobiology and clinical implications. Immunol Res. 2008;40: 18–34.

283. Matricon J, Barnich N, Ardid D. Immunopathogenesis of inflammatory bowel disease. Self Nonself. 2010;1: 299–309.

284. Lawrance IC, Rogler G, Bamias G, Breynaert C, Florholmen J, Pellino G, et al. Cellular and Molecular Mediators of Intestinal Fibrosis. J Crohns Colitis. 2015; doi:10.1016/j.crohns.2014.09.008

285. Sahebally SM, Burke JP, Chang KH, Kiernan MG, O'Connell PR, Coffey JC. Circulating fibrocytes and Crohn's disease. Br J Surg. 2013;100: 1549–1556.

286. Gruss HJ, Scott C, Rollins BJ, Brach MA. Human fibroblasts express functional IL-2 receptors formed by the IL-2R alpha-and beta-chain subunits: association of IL-2 binding with secretion of the monocyte …. The Journal of. Am Assoc Immnol; 1996; Available: http://www.jimmunol.org/content/157/2/851.short

287. Shao DD, Suresh R, Vakil V, Gomer RH, Pilling D. Pivotal Advance: Th-1 cytokines inhibit, and Th-2 cytokines promote fibrocyte differentiation. J Leukoc Biol. 2008;83: 1323–1333.

288. Maharjan AS, Pilling D, Gomer RH. Toll-like receptor 2 agonists inhibit human fibrocyte differentiation. Fibrogenesis Tissue Repair. 2010;3: 23.

289. Cibor D, Domagala-Rodacka R, Rodacki T, Jurczyszyn A, Mach T, Owczarek D. Endothelial dysfunction in inflammatory bowel diseases: Pathogenesis, assessment and implications. World J Gastroenterol. 2016;22: 1067–1077.

290. Biancheri P, Di Sabatino A, Corazza GR, MacDonald TT. Proteases and the gut barrier. Cell Tissue Res. 2013;351: 269–280.

291. Jakubowska K, Pryczynicz A, Iwanowicz P, Niewiński A, Maciorkowska E, Hapanowicz J, et al. Expressions of Matrix Metalloproteinases (MMP-2, MMP-7, and MMP-9) and Their Inhibitors (TIMP-1, TIMP-2) in Inflammatory Bowel Diseases. Gastroenterol Res Pract. 2016;2016: 2456179.

292. Monteleone G, Caruso R, Fina D, Peluso I, Gioia V, Stolfi C, et al. Control of matrix metalloproteinase production in human intestinal fibroblasts by interleukin 21. Gut. 2006;55: 1774–1780.

293. Pender SLF, MacDonald TT. Matrix metalloproteinases and the gut - new roles for old enzymes. Curr Opin Pharmacol. 2004;4: 546–550.

294. Bamba S, Andoh A, Yasui H, Araki Y, Bamba T, Fujiyama Y. Matrix metalloproteinase-3 secretion from human colonic subepithelial myofibroblasts: role of interleukin-17. J Gastroenterol. 2003;38: 548–554.

295. Nimmo ER, Prendergast JG, Aldhous MC, Kennedy NA, Henderson P, Drummond HE, et al. Genome-wide methylation profiling in Crohn's disease identifies altered epigenetic regulation of key host defense mechanisms including the Th17 pathway. Inflamm Bowel Dis. 2012;18: 889–899.

296. Wu X, Tao Y, Hou J, Meng X, Shi J. Valproic acid upregulates NKG2D ligand expression through an ERK-dependent mechanism and potentially enhances NK cell-mediated lysis of myeloma. Neoplasia. 2012;14: 1178–1189.

297. Mitomi H, Ohkura Y, Yokoyama K, Sada M, Kobayashi K, Tanabe S, et al. Contribution of TIA-1+ and granzyme B+ cytotoxic T lymphocytes to cryptal apoptosis and ulceration in active inflammatory bowel disease. Pathol Res Pract. 2007;203: 717–723.

298. Jahrsdörfer B, Vollmer A, Blackwell SE, Maier J, Sontheimer K, Beyer T, et al. Granzyme B produced by human plasmacytoid dendritic cells suppresses T-cell expansion. Blood. 2010;115: 1156–1165.

299. Monteleone G, Monteleone I, Fina D, Vavassori P, Del Vecchio Blanco G, Caruso R, et al. Interleukin-21 enhances T-helper cell type I signaling and interferon-gamma production in Crohn's disease.

Gastroenterology. 2005;128: 687–694.

300. Schulzke JD, Ploeger S, Amasheh M, Fromm A, Zeissig S, Troeger H, et al. Epithelial tight junctions in intestinal inflammation. Ann N Y Acad Sci. 2009;1165: 294–300.

301. Ravi A, Garg P, Sitaraman SV. Matrix metalloproteinases in inflammatory bowel disease: boon or a bane? Inflamm Bowel Dis. 2007;13: 97–107.
