# Supplementary material for: A systems pharmacology model for inflammatory bowel disease
Source: PLoS One. 2018 Mar 7;13(3):e0192949. doi: 10.1371/journal.pone.0192949 (PMC5841748; doi:10.1371/journal.pone.0192949)
Supplement: S3 Table — Table of alterations in patients of IBD network nodes supported by references. (PDF) [file pone.0192949.s003.pdf]

S3_Table. IBD validation.

NODE	ALTERATION	REFERENCE
PGN	Altered (dysbiosis increasing pathogen bacteria)<br>CD<br>UC	[1]
MDP		
LPS		
TLR2	Upregulated<br>UC<br>CD	[2,3]
TLR4	Upregulated<br>CD<br>UC	[4]
NOD2	Altered	[5–7]
NFkB	Altered (TRAF3IP2):<br>CD<br>UC	[8]
IL6	Upregulated<br>CD<br>UC	[9]
TNFa	Upregulated<br>CD<br>UC	[9]
TGFb	Upregulated<br>CD<br>UC	[10]
Th0	-	-
Th0_M	Upregulated<br>CD	[11]
IL18	Upregulated<br>CD<br>UC	[9]
IL1b	Upregulated<br>CD<br>UC	[9]
IFNg	Upregulated<br>CD<br>UC	[9,12]
IL23	Upregulated	[9]

	CD	
IL22	Upregulated<br>CD	[13]
IL21	Upregulated<br>CD<br>UC	[9,14]
IL17	Upregulated<br>CD<br>UC	[9,12]
IL10	Upregulated<br>CD	[15,16]
	Upregulated<br>CD<br>UC	[17]
	Upregulated<br>UC	[18]
	Not altered	[19]
	KAT2B down-regulation in IBD:<br>Downregulated<br>CD<br>UC	[20]
Th17	Upregulated<br>CD<br>UC	[21,22]
Th17_M	Upregulated<br>CD	[11]
Th1	Downregulated<br>CD<br>UC	[21]
	Upregulated-equal<br>CD<br>UC	[22]
Th2	Upregulated<br>UC	[21]
IL4	Altered	[23]
	Downregulated<br>CD	[24]

	UC	
IL15	Upregulated<br>CD<br>UC	[25]
IL12	Upregulated<br>CD	[9]
IL13	Upregulated<br>UC	[9]
Treg	Downregulated<br>CD<br>UC	[26]
	Downregulated in Blood, upregulated in inflamed IBD mucosa<br>CD<br>UC	[27]
NK	Upregulated<br>CD<br>UC	[28]
DEF	Upregulated<br>CD<br>UC	[7,29]
	Downregulated<br>CD	[30]
IL2	Upregulated (but cell cultures)<br>CD	[31]
MACR	Unknown	-
	Upregulated (no primary information, just review with no reference)<br>CD<br>UC	[9]
DC	Upregulated in tissue<br>Downregulated in blood<br>CD<br>UC	[9,32]
IEC_MICA_B	Upregulated<br>CD	[33]
IEC_ULPB1_6	Upregulated<br>CD	[33]

CD8_NKG2D	Upregulated<br>CD	[24]
NK_NKG2D	Unknown	-
CD4_NKG2D	Upregulated<br>CD	[34]
FIBROBLAST	Upregulated<br>CD<br>UC	[35]
MMPs	Upregulated<br>CD<br>UC	[36,37]
PERFOR	Altered (PERF1 mutation)<br>CD	[38,39]
GRANZB	Upregulated<br>CD<br>UC	[40]

Node expression and alterations in IBD patients. The table shows the alterations as “upregulated” when the levels are higher in CD, UC or both (IBD) patients than in healthy subjects, “downregulated” when the levels are smaller or “altered” when different reports from literature are inconclusive or contradictory**.**

REFERENCES

1. Buttó LF, Haller D. Dysbiosis in intestinal inflammation: Cause or consequence. Int J Med Microbiol. 2016; doi:10.1016/j.ijmm.2016.02.010

2. Candia E, Díaz-Jiménez D, Langjahr P, Núñez LE, de la Fuente M, Farfán N, et al. Increased production of soluble TLR2 by lamina propria mononuclear cells from ulcerative colitis patients. Immunobiology. 2012;217: 634–642.

3. Cantó E, Ricart E, Monfort D, González-Juan D, Balanzó J, Rodríguez-Sánchez JL, et al. TNFα production to TLR2 ligands in active IBD patients. Clin Immunol. 2006;119: 156–165.

4. Fukata M, Abreu MT. What are toll-like receptors and what role may they have in IBD? Inflamm Bowel Dis. 2008;14: S90–S92.

5. Netea MG, Ferwerda G, de Jong DJ, Girardin SE, Kullberg BJ, van der Meer JWM. NOD2 3020insC mutation and the pathogenesis of Crohn's disease: impaired IL-1beta production points to a loss-of-function phenotype. Neth J Med. 2005;63: 305–308.

6. Wehkamp J, Harder J, Weichenthal M, Schwab M, Schäffeler E, Schlee M, et al. NOD2 (CARD15) mutations in Crohn's disease are associated with diminished mucosal alpha-defensin expression. Gut. 2004;53: 1658–1664.

7. Klag T, Stange EF, Wehkamp J. Defective antibacterial barrier in inflammatory bowel disease. Dig Dis. 2013;31: 310–316.

8. Jostins L, Ripke S, Weersma RK, Duerr RH, McGovern DP, Hui KY, et al. Host-microbe interactions have shaped the genetic architecture of inflammatory bowel disease. Nature. 2012;491: 119–124.

9. Sartor RB. Mechanisms of disease: pathogenesis of Crohn's disease and ulcerative colitis. Nat Clin Pract Gastroenterol Hepatol. 2006;3: 390–407.

10. Gurram B, Salzman NH, Kaldunski ML, Jia S, Li BUK, Stephens M, et al. Plasma-induced signatures reveal an extracellular milieu possessing an immunoregulatory bias in treatment-naive paediatric inflammatory bowel disease. Clin Exp Immunol. 2016;184: 36–49.

11. Hedin CR, McCarthy NE, Louis P, Farquharson FM, McCartney S, Taylor K, et al. Altered intestinal microbiota and blood T cell phenotype are shared by patients with Crohn's disease and their unaffected siblings. Gut. 2014;63: 1578–1586.

12. Rovedatti L, Kudo T, Biancheri P, Sarra M, Knowles CH, Rampton DS, et al. Differential regulation of interleukin 17 and interferon gamma production in inflammatory bowel disease. Gut. 2009;58: 1629–1636.

13. Brand S, Beigel F, Olszak T, Zitzmann K, Eichhorst ST, Otte J-M, et al. IL-22 is increased in active Crohn's disease and promotes proinflammatory gene expression and intestinal epithelial cell migration. Am J Physiol Gastrointest Liver Physiol. 2006;290: G827–38.

14. Gharibi T, Majidi J, Kazemi T, Dehghanzadeh R, Motallebnezhad M, Babaloo Z. Biological effects of IL-21 on different immune cells and its role in autoimmune diseases. Immunobiology. 2016;221: 357–367.

15. Marlow GJ. Why interleukin-10 supplementation does not work in Crohn's disease patients. World J Gastroenterol. 2013;19: 3931.

16. Wang AH, Lam W-J, Han D-Y, Ding Y, Hu R, Fraser AG, et al. The effect of IL-10 genetic variation and interleukin 10 serum levels on Crohn's disease susceptibility in a New Zealand population. Hum Immunol. 2011;72: 431–435.

17. Kucharzik T, Stoll R, Lügering N. Circulating antiinflammatory cytokine IL-10 in patients with inflammatory bowel disease (IBD). Clinical &. Wiley Online Library; 1995; Available: http://onlinelibrary.wiley.com/doi/10.1111/j.1365-2249.1995.tb03721.x/abstract

18. Mitsuyama K, Tomiyasu N, Takaki K, Masuda J, Yamasaki H, Kuwaki K, et al. Interleukin-10 in the pathophysiology of inflammatory bowel disease: increased serum concentrations during the recovery phase. Mediators Inflamm. 2006;2006: 26875.

19. Nielsen OH, Køppen T, Rüdiger N, Horn T, Eriksen J, Kirman I. Involvement of interleukin-4 and -10 in inflammatory bowel disease. Dig Dis Sci. 1996;41: 1786–1793.

20. Bai AHC, Wu WKK, Xu L, Wong SH, Go MY, Chan AWH, et al. Dysregulated Lysine Acetyltransferase 2B Promotes Inflammatory Bowel Disease Pathogenesis Through Transcriptional Repression of Interleukin-10. J Crohns Colitis. 2016;10: 726–734.

21. Li J, Ueno A, Fort Gasia M, Luider J, Wang T, Hirota C, et al. Profiles of Lamina Propria T Helper Cell Subsets Discriminate Between Ulcerative Colitis and Crohn's Disease. Inflamm Bowel Dis. 2016; 1.

22. Calderón-Gómez E, Bassolas-Molina H, Mora-Buch R, Dotti I, Planell N, Esteller M, et al. Commensal-specific CD4+ Cells From Patients With Crohn's Disease Have a T-helper 17 Inflammatory Profile. Gastroenterology. 2016; doi:10.1053/j.gastro.2016.05.050

23. Hong J, Leung E, Fraser AG, Merriman TR, Vishnu P, Krissansen GW. IL4, IL10, IL16, and TNF polymorphisms in New Zealand Caucasian Crohn's disease patients. Int J Colorectal Dis. 2008;23: 335–337.

24. Yadav V, Varum F, Bravo R, Furrer E, Bojic D, Basit AW. Inflammatory bowel disease: exploring gut pathophysiology for novel therapeutic targets. Transl Res. 2016; doi:10.1016/j.trsl.2016.04.009

25. DePaolo RW, Abadie V, Tang F, Fehlner-Peach H, Hall JA, Wang W, et al. Co-adjuvant effects of retinoic acid and IL-15 induce inflammatory immunity to dietary antigens. Nature. 2011;471: 220–224.

26. Chamouard P, Monneaux F, Richert Z, Voegeli A-C, Lavaux T, Gaub MP, et al. Diminution of Circulating CD4+ CD25high T Cells in Naïve Crohn's Disease. Dig Dis Sci. Springer; 2009;54: 2084–2093.

27. Maul J, Loddenkemper C, Mundt P, Berg E, Giese T, Stallmach A, et al. Peripheral and intestinal regulatory CD4+ CD25(high) T cells in inflammatory bowel disease. Gastroenterology. 2005;128: 1868–1878.

28. Yadav PK, Chen C, Liu Z. Potential role of NK cells in the pathogenesis of inflammatory bowel disease. J Biomed Biotechnol. 2011;2011: 348530.

29. Biswas A, Petnicki-Ocwieja T, Kobayashi KS. Nod2: a key regulator linking microbiota to intestinal mucosal immunity. J Mol Med . 2012;90: 15–24.

30. Courth LF, Ostaff MJ, Mailänder-Sánchez D, Malek NP, Stange EF, Wehkamp J. Crohn's disease-derived monocytes fail to induce Paneth cell defensins. Proc Natl Acad Sci U S A. 2015;112: 14000–14005.

31. Katz LH, Kopylov U, Fudim E, Yavzori M, Picard O, Ungar B, et al. Expression of IL-2, IL-17 and TNF-alpha in patients with Crohn's disease treated with anti-TNF antibodies. Clin Res Hepatol Gastroenterol. 2014;38: 491–498.

32. Ng SC, Plamondon S, Al-Hassi HO, English N, Gellatly N, Kamm MA, et al. A novel population of human CD56+ human leucocyte antigen D-related (HLA-DR+) colonic lamina propria cells is associated with inflammation in ulcerative colitis. Clin Exp Immunol. 2009;158: 205–218.

33. Pariente B, Mocan I, Camus M, Dutertre C-A, Ettersperger J, Cattan P, et al. Activation of the receptor NKG2D leads to production of Th17 cytokines in CD4+ T cells of patients with Crohn's disease. Gastroenterology. 2011;141: 217–26, 226.e1–2.

34. Allez M, Tieng V, Nakazawa A, Treton X, Pacault V, Dulphy N, et al. CD4+NKG2D+ T cells in Crohn's disease mediate inflammatory and cytotoxic responses through MICA interactions. Gastroenterology. 2007;132: 2346–2358.

35. Lawrance IC, Rogler G, Bamias G, Breynaert C, Florholmen J, Pellino G, et al. Cellular and Molecular Mediators of Intestinal Fibrosis. J Crohns Colitis. 2015; doi:10.1016/j.crohns.2014.09.008

36. Biancheri P, Di Sabatino A, Corazza GR, MacDonald TT. Proteases and the gut barrier. Cell Tissue Res. 2013;351: 269–280.

37. Jakubowska K, Pryczynicz A, Iwanowicz P, Niewiński A, Maciorkowska E, Hapanowicz J, et al. Expressions of Matrix Metalloproteinases (MMP-2, MMP-7, and MMP-9) and Their Inhibitors (TIMP-1, TIMP-2) in Inflammatory Bowel Diseases. Gastroenterol Res Pract. 2016;2016: 2456179.

38. Voskoboinik I, Whisstock JC, Trapani JA. Perforin and granzymes: function, dysfunction and human pathology. Nat Rev Immunol. 2015;15: 388–400.

39. Nimmo ER, Prendergast JG, Aldhous MC, Kennedy NA, Henderson P, Drummond HE, et al. Genome-wide methylation profiling in Crohn's disease identifies altered epigenetic regulation of key host defense mechanisms including the Th17 pathway. Inflamm Bowel Dis. 2012;18: 889–899.

40. Cupi ML, Sarra M, Marafini I, Monteleone I, Franzè E, Ortenzi A, et al. Plasma cells in the mucosa of patients with inflammatory bowel disease produce granzyme B and possess cytotoxic activities. J Immunol. 2014;192: 6083–6091.
