# Supplementary material for: A systems pharmacology model for inflammatory bowel disease
Source: PLoS One. 2018 Mar 7;13(3):e0192949. doi: 10.1371/journal.pone.0192949 (PMC5841748; doi:10.1371/journal.pone.0192949)
Supplement: S1 File — Document with detailed description of the methodology. (DOCX) [file pone.0192949.s004.docx]

# Supporting information: METHODS

**2.3. BOOLEAN NETWORK BUILDING AND R IMPLEMENTATION**

Activation or inhibition signals between nodes are described as combinations of the logic operators: AND (&), OR (|) and NOT (!) condensed in a mathematical expression called boolean function for each node. To obtain more realistic simulation results, we also introduced activation/inhibition thresholds, positive modulators and negative modulators.

Threshold operator[[1]](https://paperpile.com/c/9DXRk6/ISTqS) is represented with the U and $\bigcap$ notation in the boolean functions (Table 1 from results). The U and $\bigcap$ operators require a duration argument which indicates the number of previous iteration that must be evaluated for a regulator node[[2]](https://paperpile.com/c/9DXRk6/2FHJ).For example, as equation (1) shows, the nodes Perforin (PERFOR), Granzyme B (GRANZB) and Defensins (DEF) inhibits Peptidoglycan (PGN) activation when any of these three nodes have been activated for 6 consecutive iterations (AG_elim=6).

$PGN= ! (\bigcap_{i=1}^{AG_{-}elim=6} {PERFOR}^{t-i} |\bigcap_{i=1}^{AG_{-}elim=6} {GRANZB}^{t-i} | \bigcap_{i=1}^{AG_{-}elim=6} {DEF}^{t-i} )$

(1)

Furthermore, there are many cases in the literature in which a node A is not able to activate another node B, but A can increase or prolong B expression if B is activated by other signals. We considered this relationship as a positive modulation of node B by node A and we expressed it with the following combination of Boolean operators: B* = Activators OR (B AND A)[[2]](https://paperpile.com/c/9DXRk6/2FHJ). Positive modulators in IBD network can be used for cell, cytokine or receptor nodes. Interleukin 22 (IL22) boolean function includes an example of positive modulator for cytokines (equation 2). The Boolean function of this modulation is:

$IL22=Activators |((IL22 \& Th0 \& IL21) \&!(\bigcap_{i=1}^{upreg\_cyt=3} {IL22}^{t-i}\&\bigcap_{i=1}^{upreg\_cyt=3} {Th0}^{t-i}\&\bigcap_{i=1}^{upreg\_cyt=3} {IL21}^{t-i}$*))*

(2)

Meaning that once IL22 has been activated, Th0 and IL21 can prolong the activation, but this prolongation lasts three iterations (upreg_cyt=3).

Similarly, if node A cannot directly inhibit node B but it can decrease or shorten its expression it was considered as a negative modulation, expressed as *B*=Activators AND NOT (B AND A)* [[2]](https://paperpile.com/c/9DXRk6/2FHJ). Negative modulators in IBD network can be used for cell or cytokine nodes. Interleukin 1 beta (IL1b) boolean function includes an example of negative modulator for cytokines (equation 3). IL10 node, act as negative modulator for IL1b node[[3,4]](https://paperpile.com/c/9DXRk6/FkzLC+n1vT6). The Boolean function of this modulation would be:

$IL1b=Activators \&! (IL1b \&\bigcup_{i=1}^{downreg\_cyt=4} {IL10}^{t-i})$

(3)

Meaning that once IL1b has been activated, IL10 can inhibit IL1b activation, but it is necessary for IL10 to be activated during 4 consecutive iterations (downreg_cyt=4).

**2.4 SIMULATIONS**

IBD scenarios were recreated by simulating chronic exposure to bacterial antigens. Muramyl dipeptide (MDP), Peptidoglycan (PGN) and Lipopolysaccharide (LPS) antigens were chosen because these antigens seem to play a critical role in the development and pathophysiology of IBD[[5–7]](https://paperpile.com/c/9DXRk6/rsK62+N3YKb+uf9Qs). These three antigens were considered the input nodes of the model and their boolean functions were identical. The IBD network can be used to simulate different scenarios, such as chronic infection in non IBD individual or IBD individual. Table S1 shows the boolean equation and the value for each threshold parameter to simulate these scenarios:

***S1 Table.* Ag boolean function for the simulation of different scenarios**. Chronic infection in non IBD individual and IBD individual. Ag means antigen, which are any of the network initial conditions (PGN, MDP or LPS).

| **BOOLEAN FUNCTION** | **SIMULATED CONDITION** |
| --- | --- |
| $Ag=!(\bigcap_{i=1}^{AG_{-}elim=1} {PERFOR}^{t-i}\vert\bigcap_{i=1}^{AG_{-}elim=1} {GRANZB}^{t-i} \vert\bigcap_{i=1}^{AG_{-}elim=1} {DEF}^{t-i} )$ | Chronic infection in non IBD individual |
| $Ag=!(\bigcap_{i=1}^{AG_{-}elim=6} {PERFOR}^{t-i}\vert\bigcap_{i=1}^{AG_{-}elim=6} {GRANZB}^{t-i} \vert\bigcap_{i=1}^{AG_{-}elim=6} {DEF}^{t-i} )$ | IBD individual |

As table S1 shows, IBD individuals were simulated with: (i) Chronic antigen exposure, which is in line with intestinal dysbiosis in IBD patients according to the literature (ii) Impairment in antigen elimination, with the threshold operator Ag_elim=6 [[4–6]](https://paperpile.com/c/5MxVsQ/vMxf+iCzu+Qmqe).

We also aimed to incorporate existing knowledge on the relative duration of the processes. These processes include ligand–receptor binding, signal transduction, cytokine production or cell differentiation. The final value for each positive, negative or threshold operator was found through fine tuning to the desired output, an attractor state for each node in sense with a chronic inflammatory process. Regarding threshold operators for cell differentiation or ligand–receptor binding, we used as guide the work developed by Thakar et al.[[1]](https://paperpile.com/c/9DXRk6/ISTqS). Table S2 summarizes the selected value for all the IBD network operators.

***S2 Table.* IBD network simulation conditions.** Positive modulator operators are used in cell (upreg_cell), cytokine (upreg_cytokines) or receptor (upreg_rec) nodes. The negative modulator operators are used in cell (downreg_cell) or cytokine (downreg_cytokines) nodes. Threshold operators represent a delay in cell differentiation from Activated  CD4+ T cell to T regulatory cell (THR_Th0_Treg and UTh0) or in ligand–receptor binding (THR_LIGANDS_NKG2D and THR_NOD2_DEF).

| **POSITIVE AND NEGATIVE OPERATORS** | | **THRESHOLD OPERATORS** | |
| --- | --- | --- | --- |
| NAME | TIME DELAY | NAME | TIME DELAY |
| upreg_cell  upreg_cyt  upreg_rec  downreg_cell  downreg_cyt | *2*  *3*  *2*  *2*  *4* | THR_Th0_Treg  THR_Th0  THR_LIGANDS_NKG2D  THR_NOD2_DEF  AG_elim | *3*  *3*  *3*  *5*  *6 For IBD simulation* |

**REFERENCES**

1. T[hakar J, Pilione M, Kirimanjeswara G, Harvill ET, Albert R. Modeling systems-level regulation of host immune responses. PLoS Comput Biol. 2007;3: e109.](http://paperpile.com/b/9DXRk6/ISTqS)

2. [Irurzun-Arana I, Pastor JM, Trocóniz IF, Gómez-Mantilla JD. Advanced Boolean modeling of biological networks applied to systems pharmacology. Bioinformatics. 2017; doi:](http://paperpile.com/b/9DXRk6/2FHJ)[10.1093/bioinformatics/btw747](http://dx.doi.org/10.1093/bioinformatics/btw747)

3. [D’Andrea A, Aste-Amezaga M, Valiante NM, Ma X, Kubin M, Trinchieri G. Interleukin 10 (IL-10) inhibits human lymphocyte interferon gamma-production by suppressing natural killer cell stimulatory factor/IL-12 synthesis in accessory cells. J Exp Med. 1993;178: 1041–1048.](http://paperpile.com/b/9DXRk6/FkzLC)

4. [de Waal Malefyt R, Abrams J, Bennett B, Figdor CG, de Vries JE. Interleukin 10(IL-10) inhibits cytokine synthesis by human monocytes: an autoregulatory role of IL-10 produced by monocytes. J Exp Med. 1991;174: 1209–1220.](http://paperpile.com/b/9DXRk6/n1vT6)

5. [Buttó LF, Haller D. Dysbiosis in intestinal inflammation: Cause or consequence. Int J Med Microbiol. 2016; doi:](http://paperpile.com/b/9DXRk6/rsK62)[10.1016/j.ijmm.2016.02.010](http://dx.doi.org/10.1016/j.ijmm.2016.02.010)

6. [Glasser A-L, Darfeuille-Michaud A. Abnormalities in the handling of intracellular bacteria in Crohn’s disease: a link between infectious etiology and host genetic susceptibility. Arch Immunol Ther Exp . 2008;56: 237–244.](http://paperpile.com/b/9DXRk6/N3YKb)

7. [Wehkamp J, Götz M, Herrlinger K, Steurer W, Stange EF. Inflammatory Bowel Disease. Dtsch Arztebl Int. 2016;113: 72–82.](http://paperpile.com/b/9DXRk6/uf9Qs)
