# Supplementary material for: A systems pharmacology model for inflammatory bowel disease
Source: PLoS One. 2018 Mar 7;13(3):e0192949. doi: 10.1371/journal.pone.0192949 (PMC5841748; doi:10.1371/journal.pone.0192949)
Supplement: S3 File — Html tutorial about how to reproduce the results from the present manuscript with the SPIDDOR package. (HTML) [file pone.0192949.s006.html]

A Systems Pharmacology Model for IBD


# A Systems Pharmacology Model for IBD

## R Package: SPIDDOR

Hereby all the steps to simulate the presented model and reproduce the results from the manuscript “A Systems Pharmacology Model for Inflammatory Bowel Disease” using SPIDDOR R package (Irurzun-Arana et al., 2017) are found.

**If you work with Windows:**

First of all, install Rtools and load it. You can do it from https://cran.r-project.org/bin/windows/Rtools/ or by running the following commands:

```
# install.packages("installr") 
library(installr)
# install.Rtools()
```

Afterwards, you have to install SPIDDOR package from github. You may be asked for additional packages. In that case, please install them:

```
# install.packages("devtools")
library(devtools)
# install_github("SPIDDOR/SPIDDOR")
```

Connect to Rtools to use the simulation algorithm in C++:

```
Connect2Rtools(path="C:\\Rtools") #Write the path to your Rtools folder (where it was previously installed)
```

**If you work with MAC or Linux:**

Please, follow the intructions from https://github.com/SPIDDOR/SPIDDOR/blob/master/README.md
Load SPIDDOR:

```
library(SPIDDOR)
```

## Read model file: IBD.txt

SPIDDOR needs to read the txt where the boolean equations have been defined.
Use: read.Boolean.functions
The working directory may be needed to be set to the directory where the IBD.txt provided in supplementary material has been placed:

```
# setwd("C/...")
BN <- read.Boolean.functions("IBD.txt")
```

For seeing more reading options, please run:

```
?read.Boolean.functions
```

Print IBD Network that has just been read:

```
print(BN)
```

```
## $nodes.names
##  [1] "PGN"         "MDP"         "LPS"         "TLR2"        "TLR4"       
##  [6] "NOD2"        "NFkB"        "IL6"         "TNFa"        "TGFb"       
## [11] "Th0"         "Th0_M"       "IL18"        "IL1b"        "IFNg"       
## [16] "IL23"        "IL22"        "IL21"        "IL17"        "IL10"       
## [21] "Th17"        "Th17_M"      "Th1"         "Th2"         "IL4"        
## [26] "IL15"        "IL12"        "IL13"        "Treg"        "NK"         
## [31] "DEF"         "IL2"         "MACR"        "DC"          "IEC_MICA_B" 
## [36] "IEC_ULPB1_6" "CD8_NKG2D"   "NK_NKG2D"    "CD4_NKG2D"   "FIBROBLAST" 
## [41] "PERFOR"      "GRANZB"      "MMPs"       
## 
## $Initial_conditions
## NULL
## 
## $Modulator
##       downreg_cyt         upreg_cyt        upreg_cell      downreg_cell 
##                 3                 3                 3                 3 
##         upreg_rec Thr_LIGANDS_NKG2D 
##                 3                 3 
## 
## $Arguments
##      AG_elim      Thr_Th0 Thr_Th0_Treg Thr_NOD2_DEF 
##            3            3            3            3 
## 
## $Polymorphism
##         PGN         MDP         LPS        TLR2        TLR4        NOD2 
##           1           1           1           1           1           1 
##        NFkB         IL6        TNFa        TGFb         Th0       Th0_M 
##           1           1           1           1           1           1 
##        IL18        IL1b        IFNg        IL23        IL22        IL21 
##           1           1           1           1           1           1 
##        IL17        IL10        Th17      Th17_M         Th1         Th2 
##           1           1           1           1           1           1 
##         IL4        IL15        IL12        IL13        Treg          NK 
##           1           1           1           1           1           1 
##         DEF         IL2        MACR          DC  IEC_MICA_B IEC_ULPB1_6 
##           1           1           1           1           1           1 
##   CD8_NKG2D    NK_NKG2D   CD4_NKG2D  FIBROBLAST      PERFOR      GRANZB 
##           1           1           1           1           1           1 
##        MMPs 
##           1
```

A default value for the modulators and threshold operators by SPIDDOR will be given. Please write the correct values for the modulators and threshold operators defined in the model (more information can be found in supplementary material “Supporting\_Infromation\_Methods.docx”, and in Irurzun-Arana et al., 2017):

```
BN$Modulator["downreg_cyt"]<-4
BN$Modulator["downreg_cell"]<-2
BN$Modulator["upreg_cyt"]<-3
BN$Modulator["upreg_cell"]<-2
BN$Modulator["upreg_rec"]<-2
BN$Modulator["Thr_LIGANDS_NKG2D"]<-3
BN$Arguments["Thr_Th0_Treg"]<-3
BN$Arguments["Thr_Th0"]<-3
BN$Arguments["Thr_NOD2_DEF"]<-5
BN$Arguments["AG_elim"]<-6
```

## INITIAL CONDITIONS: CHRONIC EXPOSURE

The input nodes (bacterial antigens) will be activated from the beginning of the simulation. An infection will be reproduced.

```
BN$Initial_conditions<-c("PGN","MDP","LPS")
```

## ATTRACTORS

Few repetitions, many iterations.

Attractor with antigen elimination= 6 (IBD patients):

```
BN$Arguments["AG_elim"]=6
Attr_6<-Get_Attractor.f(BN,time.steps=5000,repetitions=25)
print(Attr_6)
```

```
##         PGN         MDP         LPS        TLR2        TLR4        NOD2 
##   0.5689621   0.5697563   0.5682320   0.5697884   0.5684406   0.5693632 
##        NFkB         IL6        TNFa        TGFb         Th0       Th0_M 
##   0.6682071   0.4823533   0.5805233   0.4425831   0.4492182   1.0000000 
##        IL18        IL1b        IFNg        IL23        IL22        IL21 
##   0.3480556   0.2200560   0.3755105   0.4144543   0.6565015   0.2337834 
##        IL17        IL10        Th17      Th17_M         Th1         Th2 
##   0.6696914   0.5939779   0.2262739   1.0000000   0.2187402   0.2448151 
##         IL4        IL15        IL12        IL13        Treg          NK 
##   0.2406110   0.4554200   0.1838721   0.2403222   0.1445832   0.6486710 
##         DEF         IL2        MACR          DC  IEC_MICA_B IEC_ULPB1_6 
##   0.7924760   0.7688401   0.3735609   0.3700067   0.3669659   0.4233599 
##   CD8_NKG2D    NK_NKG2D   CD4_NKG2D  FIBROBLAST      PERFOR      GRANZB 
##   0.4439550   0.3922465   0.3804928   0.4879293   0.7546714   0.8251137 
##        MMPs 
##   0.5407530
```

Attractor with antigen elimination 1 (Healthy subjects):

```
BN$Arguments["AG_elim"]=1
Attr_1<-Get_Attractor.f(BN,time.steps=5000,repetitions=25)
print(Attr_1)
```

```
##          PGN          MDP          LPS         TLR2         TLR4 
## 0.1667107319 0.1657973801 0.1677362496 0.1680246765 0.1695228939 
##         NOD2         NFkB          IL6         TNFa         TGFb 
## 0.1670472299 0.3212033810 0.2502343468 0.4503385010 0.2768657613 
##          Th0        Th0_M         IL18         IL1b         IFNg 
## 0.0018907984 0.8858710892 0.0405319873 0.0367584024 0.2777630894 
##         IL23         IL22         IL21         IL17         IL10 
## 0.2633337339 0.4812322237 0.0010175059 0.3532908705 0.1040499940 
##         Th17       Th17_M          Th1          Th2          IL4 
## 0.0010575652 0.8851259865 0.0011617193 0.0010415415 0.0010575652 
##         IL15         IL12         IL13         Treg           NK 
## 0.2097344069 0.0434883628 0.0009694348 0.0000000000 0.5494051196 
##          DEF          IL2         MACR           DC   IEC_MICA_B 
## 0.5965789368 0.4605696431 0.2804230261 0.2572126748 0.3093298081 
##  IEC_ULPB1_6    CD8_NKG2D     NK_NKG2D    CD4_NKG2D   FIBROBLAST 
## 0.1805952810 0.2760565637 0.2575972439 0.2958298281 0.3591475384 
##       PERFOR       GRANZB         MMPs 
## 0.6622761687 0.7140327685 0.3612626687
```

Plot and compare every network node expression for IBD patients and healthy subjects under Antigen expossure:

```
#Bind the two vectors:
data<-rbind(Attr_1,Attr_6)
rownames(data)<-c("Healthy", "IBD")
#Take only the nodes that you are interested in. In this case, we would like to plot every network node:
data2= data[,c("PGN", "MDP", "LPS", "TLR2", "TLR4","NOD2", "MACR", "DC",
               "NFkB", "IL6", "TNFa", "TGFb", "Th0", "Th0_M", "IL18", "IL1b", 
               "IFNg","IL23", "IL22", "IL21", "IL17", "IL10","Th17", "Th17_M", "Th1", 
               "Th2", "IL4", "IL15", "IL12", "IL13", "Treg", "NK", "IL2", "DEF",
               "IEC_MICA_B", "IEC_ULPB1_6", "CD8_NKG2D", "NK_NKG2D", "FIBROBLAST", 
               "MMPs", "PERFOR", "GRANZB", "CD4_NKG2D")]
#Melt data
# install.packages(reshape) #in case you do not have reshape R package
library(reshape)
mdata<-melt(data2)
colnames(mdata)<-c("Chronic","node.name","value")

#Plot with ggplot2
# install.packages("ggplot2") #in case you do not have ggplot2 R package
library(ggplot2)
ggplot(mdata, aes(x = Chronic, y = value, fill=Chronic)) + 
  facet_wrap(~node.name)+
  ggtitle("CHRONIC ANTIGEN EXPOSSURE: HEALTHY VS IBD")+
  theme(plot.title = element_text(size = 18, face = "bold"))+
  geom_bar(stat = "identity",width=0.6)+
  ylab("% of activation")+ xlab(" ")+
  ylim(0, 1)+
  theme(strip.text = element_text(size=9))+
  theme(axis.text.x = element_blank())+ 
  theme(axis.title.y = element_text(size = rel(1), angle = 90))
```

## THERAPY SIMULATION FOR IBD PATIENTS

Get the attractor for the simulated therapies. Be sure that you are simulating the therapies in IBD Patient (antigen elimination = 6):

```
# BN$Arguments["AG_elim"]=6 # antigen elimination = 6
```

Knock Out of IFNg (simulated Fontolizumab):

```
BN$Arguments["AG_elim"]=6
Attr_6_KO_IFNg<-Get_Attractor.f(BN,time.steps=5000,Knockouts = "IFNg",repetitions=25)
# print(Attr_6_KO_IFNg)
```

Knock Out of TNFa (simulated Infliximab):

```
BN$Arguments["AG_elim"]=6
Attr_6_KO_TNFa<-Get_Attractor.f(BN,time.steps=5000,Knockouts = "TNFa",repetitions=25)
# print(Attr_6_KO_TNFa)
```

Knock Out of MACR (simulated Granulocyte and Monocyte Apheresis):

```
BN$Arguments["AG_elim"]=6
Attr_6_KO_MACR<-Get_Attractor.f(BN,time.steps=5000,Knockouts = "MACR",repetitions=25)
# print(Attr_6_KO_MACR)
```

Knock Out of IL17 (simulated Secukinumab):

```
BN$Arguments["AG_elim"]=6
Attr_6_KO_IL17<-Get_Attractor.f(BN,time.steps=5000,Knockouts = "IL17",repetitions=25)
# print(Attr_6_KO_IL17)
```

Overexpression of IL10 (simulated rhuIL-10):

```
BN$Arguments["AG_elim"]=6
Attr_6_OE_IL10<-Get_Attractor.f(BN,time.steps=5000,Over_expr="IL10",repetitions=25)
# print(Attr_6_OE_IL10)
```

Knock Out of IL12 and IL23 (Ustekinumab):

```
BN$Arguments["AG_elim"]=6
Attr_6_KO_IL12_IL23<-Get_Attractor.f(BN,time.steps=5000,Knockouts = c("IL12", "IL23"),repetitions=25)
# print(Attr_6_KO_IL12_IL23)
```

Knock Out of IL2 (Basiliximab or Daclizumab):

```
BN$Arguments["AG_elim"]=6
Attr_KO_IL2<-Get_Attractor.f(BN,time.steps=5000,Knockouts = "IL2",repetitions=25)
# print(Attr_KO_IL2)
```

Compare the output node (MMPs relative expression) with antigen elimination = 6 (IBD patient) for the different simulated therapies:

```
data<-rbind(Attr_6_KO_IFNg, Attr_6_KO_IL17, Attr_6_KO_TNFa, Attr_6_OE_IL10, Attr_6_KO_MACR, Attr_6, Attr_6_KO_IL2, Attr_6_KO_IL12_IL23)
rownames(data)<-c("IFNg mAb", "IL17 mAb", "TNFa mAb", "rhulL-10","GMA", "Untreated", "IL2 mAb", "IL12-IL23 mAb")
#Take only the nodes that you are interested in In this case, we are interested in our output node, MMPs:
data2= data[,"MMPs"]
#Melt data
library(reshape)
mdata<-melt(data2)
#Plot with ggplot2

ggplot(mdata, aes(x=factor(rownames(mdata), levels=c("Untreated","TNFa mAb","GMA","IL17 mAb","rhulL-10","IFNg mAb","IL2 mAb","IL12-IL23 mAb" )),y = value,fill=rownames(mdata))) + 
  geom_bar(stat = "identity",width=0.6)+
  scale_fill_manual(values = c("Untreated"="#00BFC4","rhulL-10"="#fed976", "IFNg mAb"="#F8766D","IL17 mAb"="#fdae6b", "GMA"="#addd8e", "TNFa mAb"="#31a354", "IL2 mAb"="#fa9fb5", "IL12-IL23 mAb"="#bdbdbd"))+
  ylab("% of activation")+ xlab(" ")+
  ggtitle("MMPs")+
  theme(plot.title = element_text(size = 14, face = "bold",hjust = 0.5))+
  ylim(0, 1)+
  guides(fill = "none")+
  theme(strip.text = element_text(size=50))+
  theme(axis.title.y = element_text(size = rel(0.8), angle = 90)) + 
  theme(axis.text.x = element_text(angle = 90, hjust = 1))+
  theme(text = element_text(size=10))
```

## PERTURBATION ANALYSIS AND CLUSTERING

Knock-out matrix:

```
BN$Arguments["AG_elim"]=6
# KO
KO.m<-KO_matrix.f(BN,time.steps=999,repetitions=24,asynchronous=TRUE)
```

```
## [1] 0.02325581
## [1] 0.04651163
## [1] 0.06976744
## [1] 0.09302326
## [1] 0.1162791
## [1] 0.1395349
## [1] 0.1627907
## [1] 0.1860465
## [1] 0.2093023
## [1] 0.2325581
## [1] 0.255814
## [1] 0.2790698
## [1] 0.3023256
## [1] 0.3255814
## [1] 0.3488372
## [1] 0.372093
## [1] 0.3953488
## [1] 0.4186047
## [1] 0.4418605
## [1] 0.4651163
## [1] 0.4883721
## [1] 0.5116279
## [1] 0.5348837
## [1] 0.5581395
## [1] 0.5813953
## [1] 0.6046512
## [1] 0.627907
## [1] 0.6511628
## [1] 0.6744186
## [1] 0.6976744
## [1] 0.7209302
## [1] 0.744186
## [1] 0.7674419
## [1] 0.7906977
## [1] 0.8139535
## [1] 0.8372093
## [1] 0.8604651
## [1] 0.8837209
## [1] 0.9069767
## [1] 0.9302326
## [1] 0.9534884
## [1] 0.9767442
## [1] 1
```

By default, the function Create\_heatmap colour the heatmap when changes in the attractor states are greater than 20%. To change this value, use the sensitivity parameter:

```
Create_heatmap(KO.m)
```

There is no need to repeat the calculation of the KO\_matrix. For example:

```
# Create_heatmap(KO.m,sensitivity = 0.1)
```
